# Supplementary material for: Olefin-accelerated solid-state C–N cross-coupling reactions using mechanochemistry
Source: Nat Commun. 2019 Jan 10;10:111. doi: 10.1038/s41467-018-08017-9 (PMC6328594; doi:10.1038/s41467-018-08017-9)
Supplement: Supplementary file 1 — Supplementary Information [file 41467_2018_8017_MOESM1_ESM.pdf]

Supplementary Information

Olefin-Accelerated Solid-State C–N Cross-Coupling Reactions Using Mechanochemistry

*Koji Kubota**[a], *Tamae Seo*[a], *Katsumasa Koide*[a], *Yasuchika Hasegawa*[a,b], *Hajime Ito**[a,b]

[a]*Division of Applied Chemistry, Graduate School of Engineering, Hokkaido University, Sapporo, Hokkaido 060-8628, Japan.*

[b]*Institute for Chemical Reaction Design and Discovery (WPI-ICReDD), Hokkaido University, Sapporo, Hokkaido 060-8628, Japan.*

Supplementary Methods

Chemicals and Instrumentation

The starting materials expect for 4-bromo-3',4',5',6'-tetraphenyl-1,1':2',1''-terphenyl (**1z**), 5,15-dibromo-10,20-bis(3,5-dimethylphenyl)porphyrin (**1aa**) and (10-bromoanthracen-9-yl)dimesitylborane (**1ab**) were obtained from commercial suppliers and used as received. **1z**[1], **1aa**[2,3] and **1ab**[4] were prepared according to the reported procedures, respectively. Solvents for substrate preparation were purchased from commercial suppliers, and further dried over molecular sieve (MS 4Å). All mechanochemical reactions were carried out using grinding vessels in a Retsch MM400 mill. Both bowls (1.5 mL or 25 mL) and balls are made of stainless. NMR spectra were recorded on JEOL JNM-ECX400P and JNM-ECS400 spectrometers (^{1}H: 392 or 396 or 399 or 401 MHz, ^{13}C: 99 or 100 MHz). Tetramethylsilane (^{1}H), $CDCl_3$ (^{13}C) was employed as external standards, respectively. Multiplicity was recorded as follows: s = singlet, brs = broad singlet, d = doublet, t = triplet, q = quartet, quint = quintet, sept = septet, o = octet, m = multiplet. Dibromomethane was used as an internal standard to determine NMR yields. GLC analyses were conducted with a Shimazu GC-2014 or GC-2025 equipped with ULBON HR-1 glass capillary column (Shinwa Chemical Industries) and a FID detector. High-resolution mass spectra were recorded at the Global Facility Center, Hokkaido University. MALDI-TOF mass spectra were recorded at the Open Facility Center, Hokkaido University. Powder diffraction data were recorded on a Rigaku SmartLab diffractometer with Cu-K_α radiation and D/teX Ultra detector covering 5-60° (2θ). Thermography was recorded with an NEC Avio Thermo GEAR G120. Transmission electron microscopy (TEM) analysis was carried out at "Joint-Use Facilities: Laboratory of Nano-Micro Material Analysis", Hokkaido University.

General Procedure for Solid-State C–N Coupling Using a Ball Mill

Aryl halide **1** (0.5 mmol), diarylamine **2** (0.5 mmol, 1.0 equiv), $Pd(OAc)_2$ (0.025 mmol, 5 mol %) were placed in a ball milling vessel (stainless, 1.5 mL) loaded with one grinding ball (stainless, diameter: 3 mm). The ball milling vessel was then transferred to a glovebox. In a glovebox, *t*-Bu_3P (0.025 mmol, 5 mol %) and Na(O-*t*-Bu) (0.75 mmol, 1.5 equiv) were placed in the milling vessel. After the milling vessel was removed from glovebox, the vessel was opened under air, then 1,5-cod (0.20 μL mg^{-1}) was added via syringe. After the vessel was closed in air without the purge with inert gas, the vessel was placed in the ball mill (Retch MM400, 99 min at 30Hz). After 99 min, the mixture was passed through a short silica gel column eluting with EtOAc to remove inorganic salts. The crude mixture was then purified by flash column chromatography (SiO_2, CH_2Cl_2/hexane, typically 0-15:85) to give the corresponding arylamines **3**.

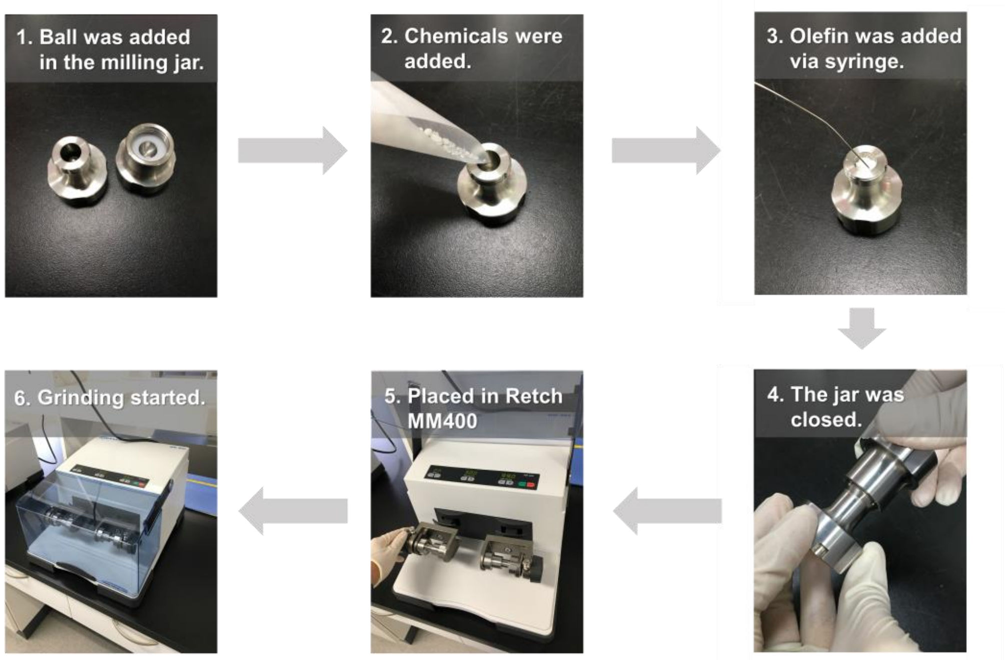

Supplementary Figure 1. How to set up the mechanochemical reactions.

Procedure for Solid-State C–N Coupling on a Gram Scale

1b (7.0 mmol, 1.970 g), **2c** (7.0 mmol, 1.381 g, 1.0 equiv), $Pd(OAc)_2$ (0.14 mmol, 31.4 mg, 2 mol %) were placed in a ball milling vessel (stainless, 25 mL) loaded with 4 grinding balls (stainless, diameter: 10 mm). The ball milling vessel was then transferred to a glovebox. In a glovebox, *t*-Bu_3P (0.14 mmol, 28.4 mg, 2 mol %) and Na(O-*t*-Bu) (10.5 mmol, 1.010 g, 1.5 equiv) were placed in the milling vessel. After the milling vessel was removed from glovebox, the vessel was opened under air, then 1,5-cod (840 μL, 0.20 μL mg^{-1}) was added quickly. After the vessel was closed without the purge with inert gas, the vessel was placed in the ball mill (Retch MM400, 99 min at 30Hz). After 99 min, the mixture was passed through a short silica gel column eluting with EtOAc to remove inorganic salts. The crude mixture was then purified by reprecipitation from CH_2Cl_2/MeOH to give the arylamine **3c** as a yellow solid (2.567 g, 92% yield).

Additional Results of Optimization Study

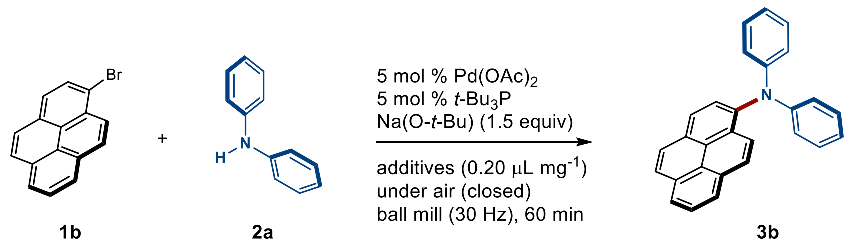

amount of 1,5-cod	conversion fo **1a** (%)[a]	yield (%)[a]
1,5-cod (40 μl) (0.13 μL mg^{-1})	78	77
1,5-cod (40 μl) (0.13 μL mg^{-1})[b]	91	90
1,5-cod (60 μl) (0.20 μL mg^{-1})	85	81
**1,5-cod (60 μl) (0.20 μL mg^{-1})[b]**	**>99**	**99**
1,5-cod (80 μl) (0.26 μL mg^{-1})	85	83
1,5-cod (80 μl) (0.26 μL mg^{-1})[b]	>99	99
1,5-cod (100 μl)((0.33 μL mg^{-1})	81	75
1,5-cod (150 μl)((0.50 μL mg^{-1})	71	66

[a]Determined by NMR analysis with an internal standard.
[b]Reaction time was 99 min.

Supplementary Figure 2. Investigation on the amount of 1,5-cod.

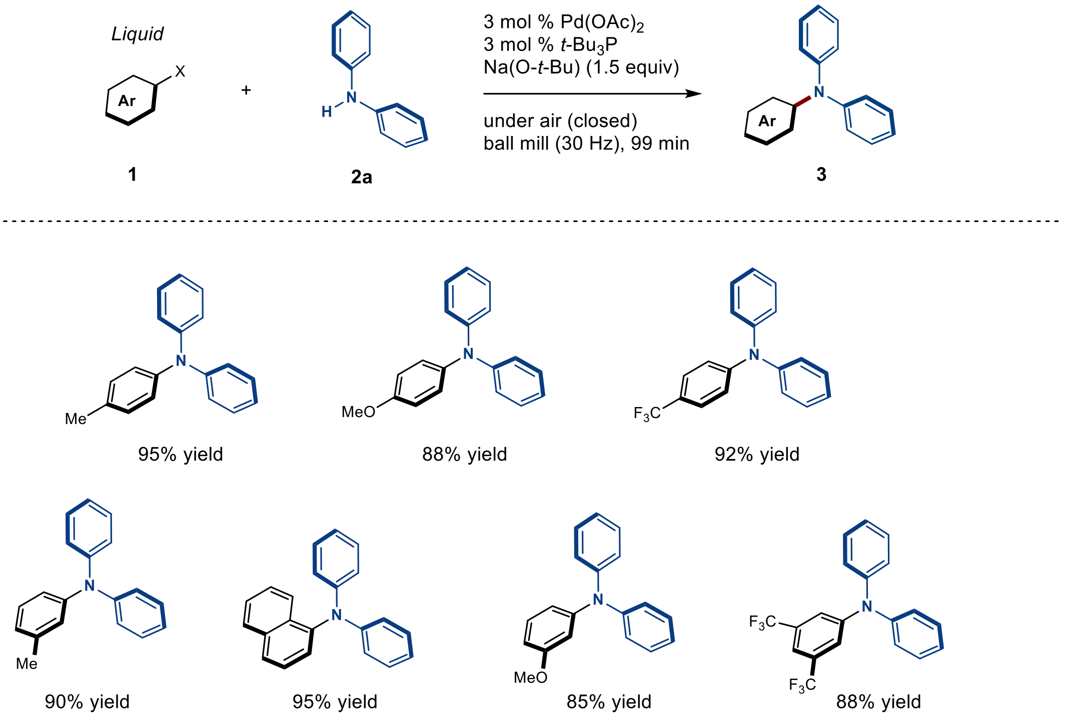

Supplementary Figure 3. Scope of liquid aryl halides without olefin additives.

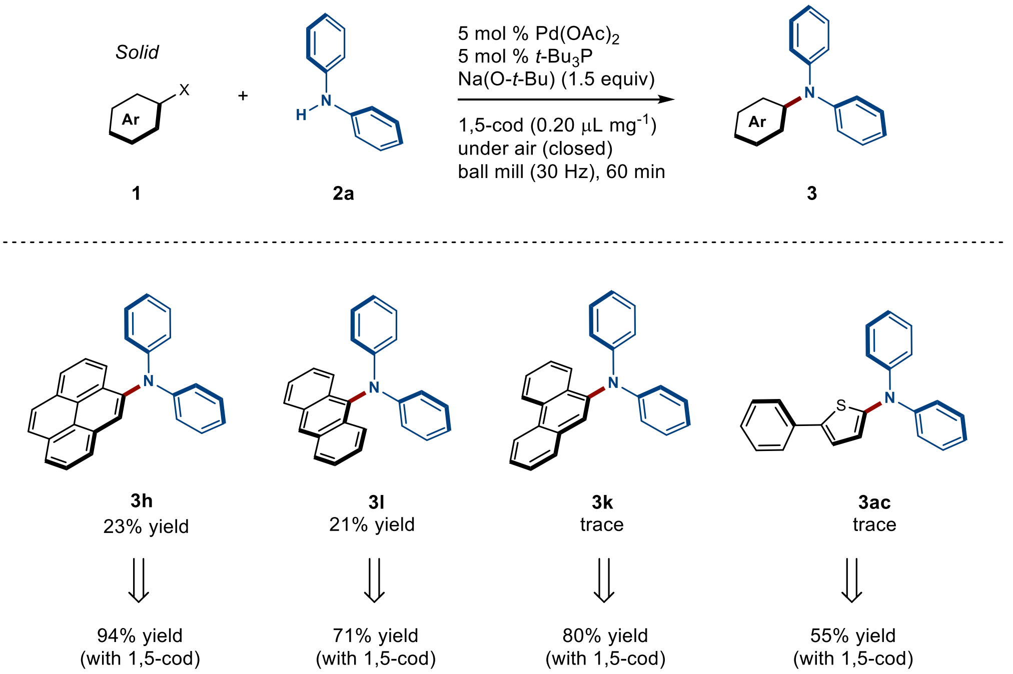

Supplementary Figure 4. Preliminary investigation on scope of solid aryl halides without olefin.

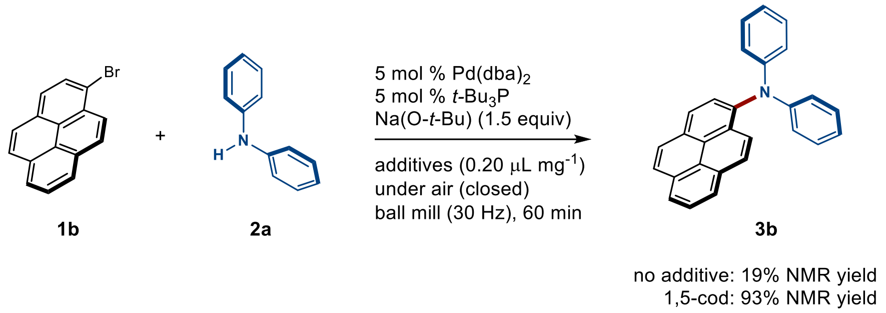

Supplementary Figure 5. The use of Pd(dba)$_2$ instead of Pd(OAc)$_2$.

Reaction Temperature Confirmed by Thermography

The temperature inside the milling jar after the solid-state coupling reaction was confirmed by thermography. The crude mixtures were prepared by the following conditions: 0.5 mmol of **1b**; 0.5 mmol of **2a**; 0.025 mmol of Pd(OAc)$_2$; 0.025 mmol of ligand; 0.75 mmol of Na(O-t-Bu); 1.5-cod (60 μl) in a stainless-steel ball-milling jar (1.5 mL) with a stainless-steel ball (3 mm); 30 Hz; 60 min. The obtained image showed that the temperature was around 30 °C, indicating that this reaction proceeded at round room temperature.

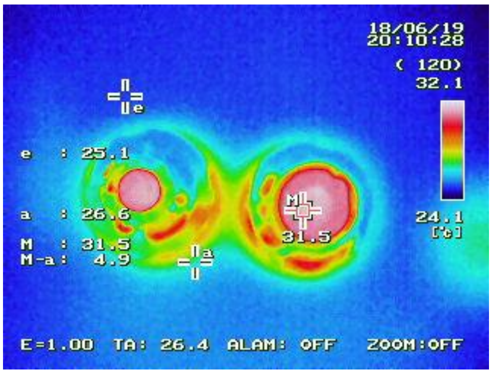

Supplementary Figure 6. Thermography image of the reaction mixture after the grinding with 30 Hz for 60 min.

Characterization of Palladium Nanoparticles by Transmission Electron Microscopy

The crude mixtures were prepared by the following conditions: 0.5 mmol of **1b**; 0.5 mmol of **2a**; 0.025 mmol of $Pd(OAc)_2$; 0.025 mmol of ligand; 0.75 mmol of Na(O-*t*-Bu); 1,5-cod (62 μl) or cyclooctane (62 μl) in a stainless-steel ball-milling jar (1.5 mL) with a stainless-steel ball (3 mm); 30 Hz; 99 min. The samples for the characterization by transmission electron microscopy (TEM) were prepared by dropping the colloidal solution of MeOH onto a copper grid covered with thin carbon film. Additional images of the reaction mixtures upon grinding for 99 min were shown in Supplementary Figure 7–9. The timescales for the aggregation of palladium particles in the reaction mixtures that contain cyclooctane or that are free of additives were shown in Supplementary Figure 10 and 11.

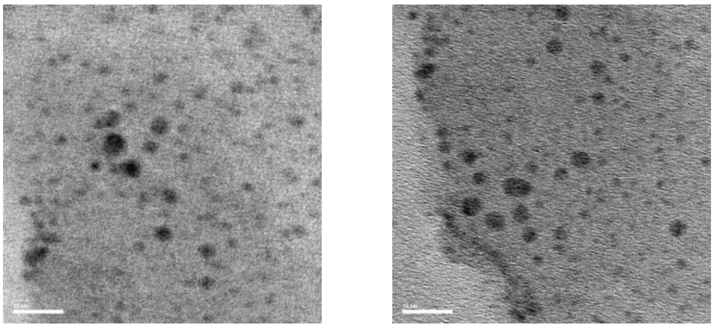

Supplementary Figure 7. TEM images of reaction mixture upon grinding with 1,5-cod for 99 min. Scale bars in TEM images (bottom left) indicate 10 nm.

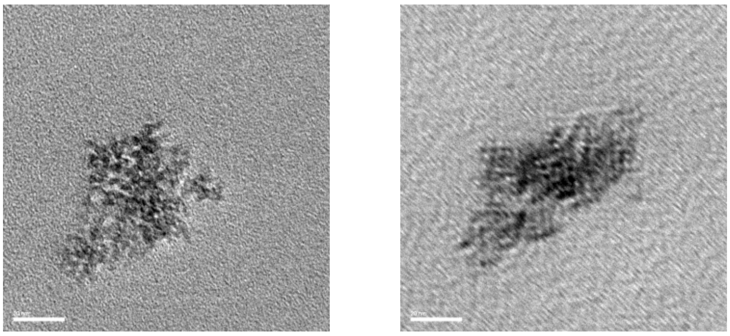

Supplementary Figure 8. TEM images of reaction mixture upon grinding with cyclooctane for 99 min. Scale bars in TEM images (bottom left) indicate 20 nm.

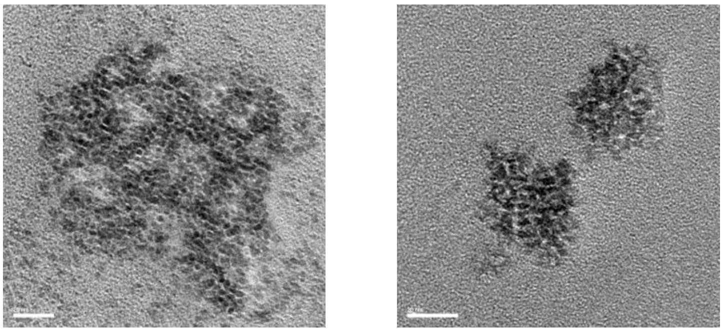

Supplementary Figure 9. TEM images of reaction mixture upon grinding without additives for 99 min. Scale bars in TEM images (bottom left) indicate 20 nm.

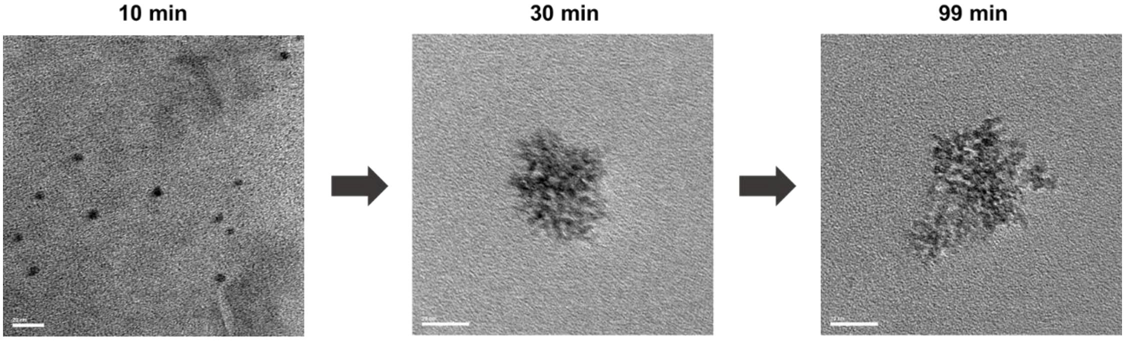

Supplementary Figure 10. Aggregation of palladium particles as a function of time in the reaction mixture containing cyclooctane. Scale bars in TEM images (bottom left) indicate 20 nm.

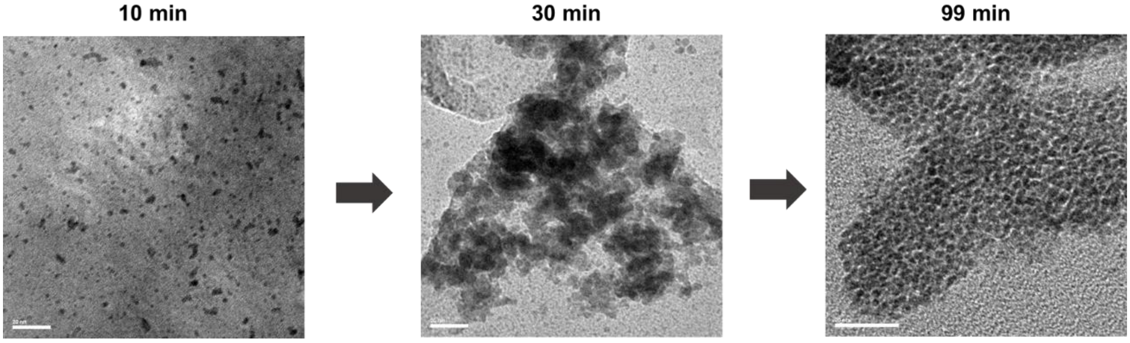

Supplementary Figure 11. Aggregation of palladium particles as a function of time in the reaction mixture without additives. Scale bars in TEM images (bottom left) indicate 20 nm.

_N,N_-Diphenylnaphthalen-1-amine (3a)

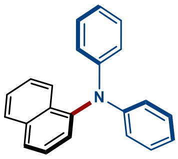

3a

The reaction was carried out with 207.1 mg (1.0 mmol) of **1a** and 169.2 mg (1.0 mmol) of **2a**. The product **3a** was obtained as a white powder (280.6 mg, 0.95 mmol, 95% yield). ^{1}H and ^{13}C NMR were in agreement with the literature.[5]

^{1}H NMR (392 MHz, $CDCl_3$, δ): 6.92 (t, *J* = 7.2 Hz, 2H), 6.99–7.06 (m, 4H), 7.18 (t, *J* = 8.1 Hz, 4H), 7.30–7.39 (m, 2H), 7.45 (q, *J* = 7.2 Hz, 2H), 7.76 (d, *J* = 8.5 Hz, 1H), 7.88 (d, *J* = 8.1 Hz, 1H), 7.94 (d, *J* = 8.5 Hz, 1H). ^{13}C NMR (99 MHz, $CDCl_3$, δ): 121.6 (*C*H), 121.8 (*C*H), 124.2 (*C*H), 126.1 (*C*H), 126.34 (*C*H), 126.36 (*C*H), 126.40 (*C*H), 127.2 (*C*H), 128.3 (*C*H), 129.0 (*C*H), 131.1 (*C*), 135.2 (*C*), 143.5 (*C*), 148.4 (*C*). HRMS-EI (*m*/*z*): $[M]^+$ calcd for $C_{22}H_{17}N$, 295.1361; found, 295.1369.

_N,N_-Diphenylpyren-1-amine (3b)

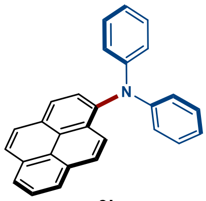

3b

The reaction was carried out with 140.6 mg (0.50 mmol) of **1b** and 84.6 mg (0.50 mmol) of **2a**. The product **3b** was obtained as a yellow powder (171.8 mg, 0.465 mmol, 93% yield). ^{1}H and ^{13}C NMR were in agreement with the literature.[6]

^{1}H NMR (392 MHz, $CDCl_3$, δ): 6.95 (t, *J* = 7.4 Hz, 2H), 7.07 (d, *J* = 7.6 Hz, 4H), 7.20 (t, *J* = 7.9 Hz, 4H), 7.83 (d, *J* = 8.1 Hz, 1H), 7.93 (d, *J* = 9.4 Hz, 1H), 7.98 (t, *J* = 7.6 Hz, 1H), 8.06 (br, s, 2H), 8.09–8.19 (m, 4H). ^{13}C NMR (99 MHz, $CDCl_3$, δ): 121.7 (*C*H), 122.0 (*C*H), 123.3 (*C*H), 124.8 (*C*), 125.1 (*C*H), 125.2 (*C*H), 126.0 (*C*H), 126.2 (*C*H), 126.3 (*C*), 127.1 (*C*H), 127.2 (*C*H), 127.6 (*C*H), 127.9 (*C*H), 128.2 (*C*), 129.1 (*C*H), 129.5 (*C*), 131.0 (*C*), 131.2 (*C*), 140.9 (*C*), 148.6 (*C*). HRMS-EI (*m*/*z*): $[M]^+$ calcd for $C_{28}H_{19}N$, 369.1518; found, 369.1521.

N,N-Di-p-tolylpyren-1-amine (3c)

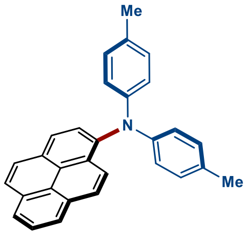

3c

The reaction was carried out with 140.6 mg (0.50 mmol) of **1b** and 98.6 mg (0.50 mmol) of **2c**. The product **3c** was obtained as a yellow powder (174.9 mg, 0.44 mmol, 88% yield). ^{1}H and ^{13}C NMR were in agreement with the literature.[6]

^{1}H NMR (392 MHz, $CDCl_3$, δ): 2.28 (s, 6H), 6.92–7.03 (m, 8H), 7.80 (d, J = 8.1 Hz, 1H), 7.90–7.99 (m, 2H), 8.03 (s, 2H), 8.09 (d, J = 7.2 Hz, 1H), 8.11–8.18 (m, 3H). ^{13}C NMR (99 MHz, $CDCl_3$, δ): 20.7 (CH$_3$), 122.1 (CH), 123.5 (CH), 124.8 (C), 124.9 (CH), 125.0 (CH), 125.9 (CH), 126.1 (CH), 126.3 (C), 126.8 (CH), 127.2 (CH), 127.3 (CH), 127.6 (CH), 127.9 (C), 129.2 (C), 129.7 (CH), 130.98 (C), 131.04 (C), 131.2 (C), 141.4 (C), 146.5 (C). HRMS-EI (m/z): $[M]^+$ calcd for $C_{30}H_{23}N$, 397.1831; found, 397.1829.

N,N-Bis(4-methoxyphenyl)pyren-1-amine (3d)

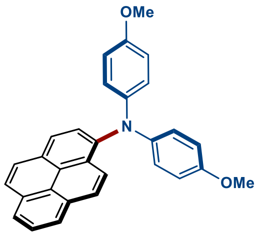

3d

The reaction was carried out with 140.6 mg (0.50 mmol) of **1b** and 114.7 mg (0.50 mmol) of **2d**. The product **3d** was obtained as a yellow powder (146.0 mg, 0.34 mmol, 68% yield). ^{1}H and ^{13}C NMR were in agreement with the literature.[7]

^{1}H NMR (392 MHz, $CDCl_3$, δ): 3.76 (s, 6H), 6.73–6.80 (m, 4H), 6.97 (d, J = 9.0 Hz, 4H), 7.75 (d, J = 8.1 Hz, 1H), 7.91 (d, J = 9.4 Hz, 1H), 7.96 (t, J = 7.6 Hz, 1H), 8.02 (brs, 2H), 8.05–8.18 (m, 4H). ^{13}C NMR (99 MHz, $CDCl_3$, δ): 55.4 (CH$_3$), 114.5 (CH), 123.5 (CH), 123.6 (CH), 124.80 (CH), 124.84 (C), 124.9 (CH), 125.8 (CH), 126.1 (CH), 126.3 (C), 126.6 (CH), 126.7 (CH), 127.2 (CH), 127.28 (C), 127.35 (CH), 128.8 (C), 131.0 (C), 131.3 (C), 142.0 (C), 143.0 (C), 154.5 (C). HRMS-EI (m/z): $[M]^+$ calcd for $C_{30}H_{23}NO_2$, 429.1729; found, 429.1715.

***N*,*N*-Di(naphthalen-2-yl)pyren-1-amine (3e)**

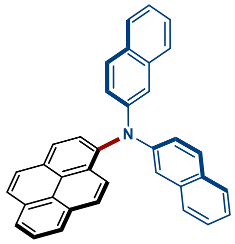

3e

The reaction was carried out with 140.6 mg (0.50 mmol) of **1b** and 134.7 mg (0.50 mmol) of **2e**. The product **3e** was obtained as a yellow powder (169.1 mg, 0.36 mmol, 72% yield).

^{1}H NMR (392 MHz, $CDCl_3$, δ): 7.29–7.41 (m, 8H), 7.45–7.50 (m, 2H), 7.69–7.78 (m, 4H), 7.88 (d, *J* = 3.6 Hz, 1H), 7.90 (d, *J* = 2.7 Hz, 1H), 7.98 (t, *J* = 7.6 Hz, 1H), 8.07–8.11 (m, 3H), 8.16–8.23 (m, 3H). ^{13}C NMR (99 MHz, $CDCl_3$, δ): 118.3 (*C*H), 123.1 (*C*H), 123.2 (*C*H), 124.2 (*C*H), 124.8 (*C*), 125.1 (*C*H), 125.2 (*C*H), 126.0 (*C*H), 126.2 (*C*H), 126.3 (*C*H), 126.4 (*C*), 126.9 (*C*H), 127.2 (*C*H), 127.5 (*C*H), 127.6 (*C*H), 127.98 (*C*), 128.03 (*C*H), 129.0 (*C*H), 129.6 (*C*), 129.7 (*C*), 131.0 (*C*), 131.2 (*C*), 134.4 (*C*), 140.8 (*C*), 146.3 (*C*). HRMS-EI (*m*/*z*): $[M]^+$ calcd for $C_{36}H_{23}N$, 469.1831; found, 469.1821.

***N*-(Naphthalen-1-yl)-*N*-phenylpyren-1-amine (3f)**

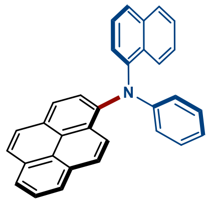

3f

The reaction was carried out with 140.6 mg (0.50 mmol) of **1b** and 109.6 mg (0.50 mmol) of **2f**. The product **3f** was obtained as a yellow powder (169.9 mg, 0.405 mmol, 81% yield).

^{1}H NMR (392 MHz, $CDCl_3$, δ): 6.77 (d, *J* = 7.6 Hz, 2H), 6.89 (t, *J* = 7.4 Hz, 1H), 7.13 (t, *J* = 7.6 Hz, 2H), 7.23–7.27 (m, 1H), 7.29–7.39 (m, 2H), 7.43–7.49 (m, 1H), 7.71 (d, *J* = 8.1 Hz, 1H), 7.77 (d, *J* = 8.1 Hz, 1H), 7.89 (d, *J* = 4.9 Hz, 1H), 7.91 (d, *J* = 5.8 Hz, 1H), 7.97 (t, *J* = 7.6 Hz, 1H), 8.02 (brs, 2H), 8.03–8.17 (m, 4H), 8.25 (d, *J* = 9.4 Hz, 1H). ^{13}C NMR (99 MHz, $CDCl_3$, δ): 120.3 (*C*H), 120.7 (*C*H), 123.4 (*C*H), 124.5 (*C*H), 124.86 (*C*), 124.93 (*C*H), 125.1 (*C*H), 125.5 (*C*H), 125.6 (*C*H), 125.7 (*C*H), 125.8 (*C*H), 126.0 (*C*H), 126.1 (*C*H), 126.3 (*C*H), 126.4 (*C*), 126.7 (*C*H), 126.8 (*C*), 127.2 (*C*H), 127.7 (*C*H), 128.5 (*C*H), 128.9 (*C*), 129.0 (*C*H), 130.2 (*C*), 131.0 (*C*), 131.3 (*C*), 135.2 (*C*), 142.4 (*C*), 144.9 (*C*), 150.7 (*C*). HRMS-EI (*m*/*z*): $[M]^+$ calcd for $C_{32}H_{21}N$, 419.1674; found, 419.1663.

***N*,*N*-Diphenylpyren-4-amine (3h)**

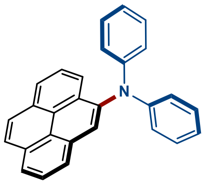

3h

The reaction was carried out with 140.6 mg (0.50 mmol) of **1h** and 84.6 mg (0.50 mmol) of **2a**. The product **3h** was obtained as a yellow powder (173.6 mg, 0.47 mmol, 94% yield).

^{1}H NMR (392 MHz, $CDCl_3$, δ): 6.91–6.98 (m, 2H), 7.04–7.10 (m, 4H), 7.20 (t, *J* = 8.1 Hz, 4H), 7.83 (d, *J* = 8.5 Hz, 1H), 7.93 (d, *J* = 9.0 Hz, 1H), 7.98 (t, *J* = 7.6 Hz, 1H), 8.06 (brs, 2H), 8.09–8.19 (m, 4H). ^{13}C NMR (100 MHz, $CDCl_3$, δ): 121.7 (*C*H), 122.0 (*C*H), 123.3 (*C*H), 124.8 (*C*), 125.0 (*C*H), 125.1 (*C*H), 126.0 (*C*H), 126.1 (*C*H), 126.3 (*C*), 127.05 (*C*H), 127.15 (*C*H), 127.6 (*C*H), 127.9 (*C*H), 128.1 (*C*), 129.1 (*C*H), 129.5 (*C*), 131.0 (*C*), 131.2 (*C*), 140.8 (*C*), 148.6 (*C*). HRMS-EI (*m*/*z*): $[M]^+$ calcd for $C_{28}H_{19}N$, 369.1518; found, 369.1506.

7-(*tert*-Butyl)-*N*,*N*-diphenylpyren-2-amine (3i)

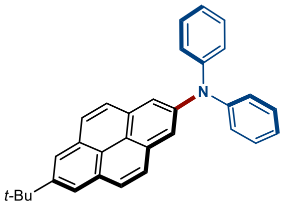

3i

The reaction was carried out with 168.2 mg (0.50 mmol) of **1i** and 85.4 mg (0.50 mmol) of **2a**. The product **3i** was obtained as a yellow powder (147.9 mg, 0.35 mmol, 70% yield).

^{1}H NMR (401 MHz, $CDCl_3$, δ): 1.57 (s, 9H), 7.03–7.09 (m, 2H), 7.17–7.22 (m, 4H), 7.26–7.32 (m, 4H), 7.82 (d, *J* = 8.4 Hz, 2H), 7.86 (s, 2H), 7.97 (d, *J* = 8.4 Hz, 2H), 8.16 (s, 2H). ^{13}C NMR (99 MHz, $CDCl_3$, δ): 32.1 (CH_3), 35.3 (*C*), 120.7 (*C*H), 121.4 (*C*), 122.6 (*C*H), 122.9 (*C*H), 123.0 (*C*), 124.4 (*C*H), 126.9 (*C*H), 128.1 (*C*H), 129.5 (*C*H), 130.5 (*C*), 132.3 (*C*), 145.7 (*C*), 148.4 (*C*), 148.5 (*C*). HRMS-EI (*m*/*z*): $[M]^+$ calcd for $C_{32}H_{27}N$, 425.2144; found, 425.2146.

***N*,*N*-Diphenylnaphthalen-2-amine (3j)**

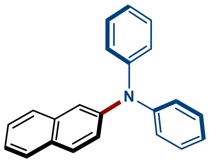

3j

The reaction was carried out with 103.4 mg (0.50 mmol) of **1j** and 85.4 mg (0.50 mmol) of **2a**. The

product **3j** was obtained as a white powder (132.3 mg, 0.45 mmol, 90% yield). ^{1}H and ^{13}C NMR were in agreement with the literature.[8]

^{1}H NMR (401 MHz, $CDCl_3$, δ): 7.01–7.07 (m, 2H), 7.11–7.16 (m, 4H), 7.23–7.30 (m, 5H), 7.31–7.41 (m, 2H), 7.42 (d, *J* = 2.4 Hz, 1H), 7.58 (d, *J* = 8.0 Hz, 1H), 7.71 (d, *J* = 8.8 Hz, 1H), 7.75 (d, *J* = 8.4 Hz, 1H). ^{13}C NMR (100 MHz, $CDCl_3$, δ): 120.3 (*C*H), 123.0 (*C*H), 124.52 (*C*H), 124.56 (*C*H), 124.6 (*C*H), 126.4 (*C*H), 127.0 (*C*H), 127.7 (*C*H), 129.0 (*C*H), 129.4 (*C*H), 130.1 (*C*), 134.5 (*C*), 145.6 (*C*), 147.9 (*C*). HRMS-ESI (*m*/*z*): [M+H]$^{+}$ calcd for $C_{22}H_{18}N$, 296.1434; found, 296.1437.

_N,N_-Diphenylphenanthren-9-amine (3k)

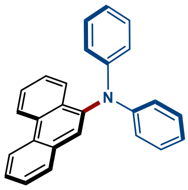

3k

The reaction was carried out with 128.6 mg (0.50 mmol) of **1k** and 84.6 mg (0.50 mmol) of **2a**. The product **3k** was obtained as a white powder (138.2 mg, 0.40 mmol, 80% yield).

^{1}H NMR (392 MHz, $CDCl_3$, δ): 6.91–6.97 (m, 2H), 7.06–7.12 (m, 4H), 7.15–7.23 (m, 4H), 7.44–7.49 (m, 1H), 7.53–7.58 (m, 1H), 7.60 (brs, 1H), 7.61–7.66 (m, 2H), 7.75 (dd, *J* = 1.3, 8.1 Hz, 1H), 8.04 (d, *J* = 8.1 Hz, 1H), 8.69 (d, *J* = 8.1 Hz, 1H), 8.73 (d, *J* = 8.1 Hz, 1H). ^{13}C NMR (99 MHz, $CDCl_3$, δ): 121.7 (*C*H), 121.8 (*C*H), 122.5 (*C*H), 123.0 (*C*H), 125.0 (*C*H), 126.5 (*C*H), 126.77 (*C*H), 126.81 (*C*H), 126.9 (*C*H), 127.6 (*C*H), 128.2 (*C*H), 129.1 (*C*H), 129.3 (*C*), 130.4 (*C*), 132.1 (*C*), 132.4 (*C*), 142.0 (*C*), 148.2 (*C*). HRMS-EI (*m*/*z*): [M]$^{+}$ calcd for $C_{26}H_{19}N$, 345.1518; found, 345.1501.

_N,N_-Diphenylanthracen-9-amine (3l)

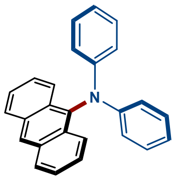

3l

The reaction was carried out with 128.6 mg (0.50 mmol) of **1l** and 84.6 mg (0.50 mmol) of **2a**. The product **3l** was obtained as a yellow powder (122.6 mg, 0.355 mmol, 71% yield). ^{1}H and ^{13}C NMR were in agreement with the literature.[9]

^{1}H NMR (392 MHz, $CDCl_3$, δ): 6.86 (dt, *J* = 1.2, 7.3 Hz, 2H), 7.03–7.18 (m, 8H), 7.34–7.47 (m, 4H), 8.05 (d, *J* = 8.5 Hz, 2H), 8.11 (d, *J* = 8.5 Hz, 2H), 8.50 (brs, 1H). ^{13}C NMR (100 MHz, $CDCl_3$, δ): 120.2 (*C*H), 121.1 (*C*H), 124.3 (*C*H), 125.5 (*C*H), 126.7 (*C*H), 126.8 (*C*H), 128.8 (*C*H), 129.1 (*C*H), 130.6 (*C*), 132.7 (*C*), 137.2 (*C*), 147.7 (*C*). HRMS-EI (*m*/*z*): [M]$^{+}$ calcd for $C_{26}H_{19}N$, 345.1518; found,

45.1512.

_N,N,_10-Triphenylanthracen-9-amine (3m)

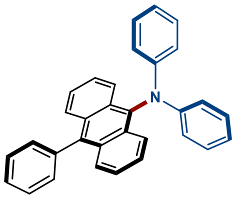

3m

The reaction was carried out with 166.6 mg (0.50 mmol) of **1m** and 87.3 mg (0.52 mmol) of **2a**. The product **3m** was obtained as a yellow powder (116.7 mg, 0.275 mmol, 55% yield).

^{1}H NMR (401 MHz, $CDCl_3$, δ): 6.86–6.92 (m, 2H), 7.10–7.21 (m, 8H), 7.29–7.42 (m, 4H), 7.46–7.51 (m, 2H), 7.51–7.64 (m, 3H), 7.71 (d, *J* = 8.8 Hz, 2H), 8.19 (d, *J* = 8.8 Hz, 2H). ^{13}C NMR (99 MHz, $CDCl_3$, δ): 120.4 (*C*H), 121.3 (*C*H), 124.5 (*C*H), 125.5 (*C*H), 126.6 (*C*H), 127.7 (*C*H), 128.5 (*C*H), 129.3 (*C*H), 130.5 (*C*), 131.4 (*C*H), 131.5 (*C*), 137.2 (*C*), 137.8 (*C*), 138.8 (*C*), 147.9 (*C*). HRMS-EI (*m*/*z*): $[M]^+$ calcd for $C_{32}H_{23}N$, 421.1831; found, 421.1813.

_N,N,_4-Triphenylnaphthalen-1-amine (3n)

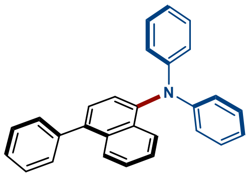

3n

The reaction was carried out with 141.4 mg (0.50 mmol) of **1n** and 87.4 mg (0.52 mmol) of **2a**. The product **3n** was obtained as a yellow powder (177.6 mg, 0.479 mmol, 96% yield).

^{1}H NMR (399 MHz, $CDCl_3$, δ): 6.95 (t, *J* = 7.2 Hz, 2H), 7.08 (d, *J* = 7.6 Hz, 4H), 7.21 (t, *J* = 8.2 Hz, 4H), 7.33–7.47 (m, 5H), 7.47–7.55 (m, 4H), 7.93 (d, *J* = 6.4 Hz, 1H), 8.04 (d, *J* = 8.0 Hz, 1H). ^{13}C NMR (99 MHz, $CDCl_3$, δ): 121.8 (*C*H), 122.0 (*C*H), 124.6 (*C*H), 126.31 (*C*H), 126.35 (*C*H), 126.8 (*C*H), 126.9 (*C*H), 127.4 (*C*H), 127.6 (*C*H), 128.4 (*C*H), 129.2 (*C*H), 130.2 (*C*H), 131.5 (*C*), 133.4 (*C*), 138.7 (*C*), 140.6 (*C*), 143.1 (*C*), 148.5 (*C*). HRMS-ESI (*m*/*z*): $[M+H]^+$ calcd for $C_{28}H_{22}N$, 372.1752; found, 372.1744.

N,N-Diphenyl-(1,1'-biphenyl)-4-amine (3o)

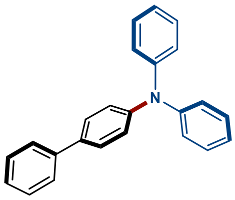

3o

The reaction was carried out with 116.4 mg (0.50 mmol) of **1o** and 84.5 mg (0.50 mmol) of **2a**. The product **3o** was obtained as a white powder (142.7 mg, 0.445 mmol, 89% yield). ^{1}H and ^{13}C NMR were in agreement with the literature.[10]

^{1}H NMR (401 MHz, $CDCl_3$, δ): 7.03 (td, J = 1.2, 7.2 Hz, 2H), 7.13 (d, J = 8.0 Hz, 6H), 7.23–7.27 (m, 3H), 7.28–7.33 (m, 2H), 7.42 (t, J = 7.6 Hz, 2H), 7.45–7.50 (m, 2H), 7.57 (d, J = 8.0 Hz, 2H). ^{13}C NMR (99 MHz, $CDCl_3$, δ): 123.0 (*C*H), 124.0 (*C*H), 124.5 (*C*H), 126.7 (*C*H), 126.9 (*C*H), 127.9 (*C*H), 128.8 (*C*H), 129.4 (*C*H), 135.2 (*C*), 140.7 (*C*), 147.2 (*C*), 147.7 (*C*). HRMS-ESI (m/z): $[M+H]^+$ calcd for $C_{24}H_{20}N$, 322.1596; found, 322.1581.

N,N-Diphenyl-(1,1':4',1''-terphenyl)-4-amine (3p)

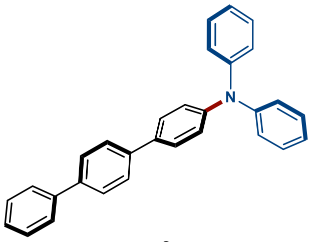

3p

The reaction was carried out with 154.4 mg (0.50 mmol) of **1p** and 86.2 mg (0.51 mmol) of **2a**. The product **3p** was obtained as a white powder (160.3 mg, 0.403 mmol, 81% yield).

^{1}H NMR (401 MHz, $CDCl_3$, δ): 7.01–7.07 (m, 2H), 7.12–7.18 (m, 6H), 7.24–7.31 (m, 4H), 7.33–7.38 (m, 1H), 7.43–7.48 (m, 2H), 7.50–7.55 (m, 2H), 7.62–7.67 (m, 6H). ^{13}C NMR (99 MHz, $CDCl_3$, δ): 123.1 (*C*H), 124.0 (*C*H), 124.6 (*C*H), 127.1 (*C*H), 127.4 (*C*H), 127.6 (*C*H), 127.8 (*C*H), 128.9 (*C*H), 129.4 (*C*H), 134.6 (*C*), 139.7 (*C*), 140.8 (*C*), 147.4 (*C*), 147.8 (*C*). HRMS-EI (m/z): $[M]^+$ calcd for $C_{30}H_{23}N$, 397.1831; found, 397.1818.

N,N-Diphenyl-1,2-dihydroacenaphthylen-5-amine (3q)

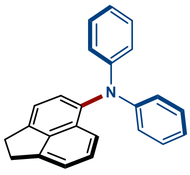

3q

The reaction was carried out with 119.0 mg (0.51 mmol) of **1q** and 85.5 mg (0.505 mmol) of **2a**. The product **3q** was obtained as a white powder (121.5 mg, 0.378 mmol, 75% yield).

^{1}H NMR (399 MHz, $CDCl_3$, δ): 3.33–3.50 (m, 4H), 6.91 (t, *J* = 7.2 Hz, 2H), 7.04 (d, *J* = 8.4 Hz, 4H), 7.18 (t, *J* = 7.6 Hz, 4H), 7.22–7.32 (m, 4H), 7.41 (d, *J* = 7.6 Hz, 1H). ^{13}C NMR (99 MHz, $CDCl_3$, δ): 30.1 (CH_2), 30.9 (CH_2), 119.6 (*C*H), 119.8 (*C*H), 119.9 (*C*H), 121.5 (*C*H), 121.9 (*C*H), 128.1 (*C*H), 128.4 (*C*H), 129.1 (*C*H), 129.4 (*C*), 139.7 (*C*), 141.1 (*C*), 144.2 (*C*), 146.4 (*C*), 148.5 (*C*). HRMS-ESI (*m*/*z*): $[M+H]^+$ calcd for $C_{24}H_{20}N$, 322.1590; found, 322.1596.

***N*,*N*-Diphenylfluoranthen-3-amine (3r)**

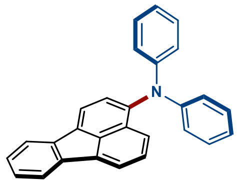

3r

The reaction was carried out with 141.7 mg (0.50 mmol) of **1r** and 85.3 mg (0.50 mmol) of **2a**. The product **3r** was obtained as a yellow powder (185.7 mg, 0.378 mmol, 99% yield).

^{1}H NMR (396 MHz, $CDCl_3$, δ): 6.94–7.01 (m, 2H), 7.07–7.13 (m, 4H), 7.18–7.26 (m, 4H), 7.28–7.42 (m, 4H), 7.62 (d, *J* = 8.7 Hz, 1H), 7.80–7.88 (m, 4H). ^{13}C NMR (99 MHz, $CDCl_3$, δ): 120.2 (*C*H), 121.2 (*C*H), 121.3 (*C*H), 121.6 (*C*H), 122.3 (*C*H), 123.0 (*C*H), 124.5 (*C*H), 127.2 (*C*H), 127.3 (*C*H), 127.7 (*C*H), 127.79 (*C*H), 127.83 (*C*), 129.3 (*C*H), 134.2 (*C*), 134.3 (*C*), 137.4 (*C*), 139.1 (*C*), 139.6 (*C*), 144.8 (*C*), 149.0 (*C*). HRMS-ESI (*m*/*z*): $[M+H]^+$ calcd for $C_{28}H_{20}N$, 370.1596; found, 370.1592.

***N*,*N*-Diphenyltriphenylen-2-amine (3s)**

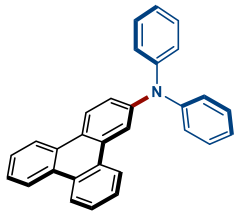

3s

The reaction was carried out with 153.6 mg (0.50 mmol) of **1s** and 84.6 mg (0.50 mmol) of **2a**. The product **3s** was obtained as a white powder (168.1 mg, 0.425 mmol, 85% yield).

^{1}H NMR (392 MHz, $CDCl_3$, δ): 7.08 (t, *J* = 7.4 Hz, 2H), 7.18–7.24 (m, 4H), 7.27–7.34 (m, 4H), 7.39 (dd, *J* = 2.3, 8.5 Hz, 1H), 7.53 (dt, *J* = 1.3, 8.6 Hz, 1H), 7.57–7.67 (m, 3H), 8.29 (d, *J* = 7.6 Hz, 1H), 8.33 (d, *J* = 2.2 Hz, 1H), 8.48–8.56 (m, 2H), 8.59–8.64 (m, 2H). ^{13}C NMR (99 MHz, $CDCl_3$, δ): 117.1 (*C*H), 122.9 (*C*H), 123.1 (*C*H), 123.2 (*C*H), 123.4 (*C*H), 123.8 (*C*H), 124.4 (*C*H), 125.0 (*C*), 126.4 (*C*H), 127.0 (*C*H), 127.2 (*C*H), 129.0 (*C*), 129.3 (*C*), 129.4 (*C*H), 129.7 (*C*), 130.0 (*C*), 130.9 (*C*),

146.9 (*C*), 147.7 (*C*). HRMS-EI (*m*/*z*): $[M]^+$ calcd for $C_{30}H_{21}N$, 395.1674; found, 395.1661.

9,9-Dimethyl-*N*,*N*-diphenyl-9*H*-fluoren-2-amine (3t)

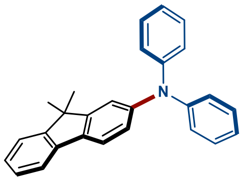

3t

The reaction was carried out with 137.1 mg (0.50 mmol) of **1t** and 85.0 mg (0.50 mmol) of **2a**. The product **3t** was obtained as a white powder (163.9 mg, 0.45 mmol, 90% yield).

^{1}H NMR (401 MHz, $CDCl_3$, δ): 1.41 (s, 6H), 7.01 (t, *J* = 7.2 Hz, 3H), 7.13 (d, *J* = 8.4 Hz, 4H), 7.19 (d, *J* = 2.0 Hz, 1H), 7.22–7.33 (m, 6H), 7.38 (d, *J* = 7.2 Hz, 1H), 7.57 (d, *J* = 8.0 Hz, 1H), 7.63 (d, *J* = 7.2 Hz, 1H). ^{13}C NMR (99 MHz, $CDCl_3$, δ): 27.2 (CH_3), 46.9 (*C*), 118.7 (*C*H), 119.5 (*C*H), 120.7 (*C*H), 122.6 (*C*H), 122.7 (*C*H), 123.4 (*C*H), 124.2 (*C*H), 126.6 (*C*H), 127.1 (*C*H), 129.3 (*C*H), 134.2 (*C*), 139.1 (*C*), 147.4 (*C*), 148.1 (*C*), 153.6 (*C*), 155.1 (*C*). HRMS-EI (*m*/*z*): $[M]^+$ calcd for $C_{27}H_{23}N$, 361.1831; found, 361.1825.

(*E*)-*N*,*N*-Diphenyl-4-styrylaniline (3u)

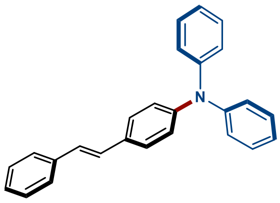

3u

The reaction was carried out with 129.6 mg (0.50 mmol) of **1u** and 84.6 mg (0.50 mmol) of **2a**. The product **3u** was obtained as a white powder (165.0 mg, 0.45 mmol, 95% yield). ^{1}H and ^{13}C NMR were in agreement with the literature.[11]

^{1}H NMR (392 MHz, $CDCl_3$, δ): 7.01–7.08 (m, 5H), 7.09–7.14 (m, 4H), 7.23–7.30 (m, 6H), 7.32–7.41 (m, 4H), 7.50 (d, *J* = 7.6 Hz, 2H). ^{13}C NMR (99 MHz, $CDCl_3$, δ): 123.0 (*C*H), 123.6 (*C*H), 124.4 (*C*H), 126.3 (*C*H), 127.0 (*C*H), 127.2 (*C*H), 127.3 (*C*H), 128.1 (*C*H), 128.6 (*C*H), 129.2 (*C*H), 131.4 (*C*), 137.6 (*C*), 147.3 (*C*), 147.5 (*C*). HRMS-EI (*m*/*z*): $[M]^+$ calcd for $C_{26}H_{21}N$, 347.1674; found, 347.1670.

N,N-Diphenyl-4-(phenylethynyl)aniline (3v)

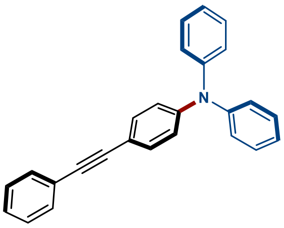

3v

The reaction was carried out with 128.6 mg (0.50 mmol) of **1v** and 84.6 mg (0.50 mmol) of **2a**. The product **3v** was obtained as a white powder (158.9 mg, 0.46 mmol, 92% yield). ^{1}H and ^{13}C NMR were in agreement with the literature.[12]

^{1}H NMR (392 MHz, $CDCl_3$, δ): 6.98–7.03 (m, 2H), 7.04–7.08 (m, 2H), 7.09–7.15 (m, 4H), 7.24–7.40 (m, 9H), 7.48–7.53 (m, 2H). ^{13}C NMR (99 MHz, $CDCl_3$, δ): 88.6 (*C*), 89.6 (*C*), 116.0 (*C*), 122.3 (*C*H), 123.46 (*C*H), 123.52 (*C*), 124.9 (*C*H), 127.9 (*C*H), 128.2 (*C*H), 129.3 (*C*H), 131.4 (*C*H), 132.5 (*C*H), 147.1 (*C*), 147.8 (*C*). HRMS-EI (*m*/*z*): $[M]^+$ calcd for $C_{26}H_{19}N$, 345.1518; found, 345.1508.

(*E*)-N,N-Diphenyl-4-styrylaniline (3w)

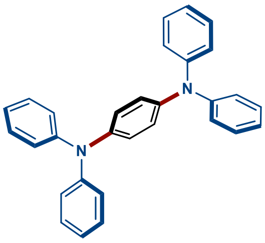

3w

The reaction was carried out with 70.8 mg (0.30 mmol) of **1w** and 101.5 mg (0.60 mmol) of **2a**. The product **3w** was obtained as a white powder (112.6 mg, 0.273 mmol, 91% yield). ^{1}H and ^{13}C NMR were in agreement with the literature.[13]

^{1}H NMR (392 MHz, $CDCl_3$, δ): 6.90–7.04 (m, 8H), 7.10 (d, J = 7.2 Hz, 8H), 7.19–7.31 (m, 8H). ^{13}C NMR (99 MHz, $CDCl_3$, δ): 122.4 (*C*H), 123.7 (*C*H), 125.4 (*C*H), 129.1 (*C*H), 142.8 (*C*), 147.8 (*C*). HRMS-EI (*m*/*z*): $[M]^+$ calcd for $C_{30}H_{24}N_2$, 412.1940; found, 412.1929.

N,N-Diphenyl-4-(1,2,2-triphenylvinyl)aniline (3x)

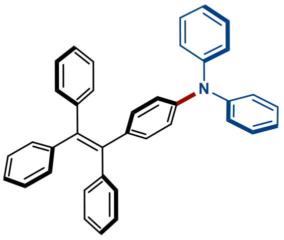

3x

The reaction was carried out with 205.7 mg (0.50 mmol) of **1x** and 84.6 mg (0.50 mmol) of **2a**. The product **3x** was obtained as a white powder (167.4 mg, 0.335 mmol, 67% yield). ^{1}H and ^{13}C NMR were in agreement with the literature.[14]

^{1}H NMR (392 MHz, $CDCl_3$, δ): 6.78 (d, J = 8.5 Hz, 2H), 6.85 (d, J = 8.5 Hz, 2H), 6.94–7.17 (m, 21H), 7.21 (t, J = 8.1 Hz, 4H). ^{13}C NMR (99 MHz, $CDCl_3$, δ): 122.6 (*C*H), 122.7 (*C*H), 124.2 (*C*H), 126.25 (*C*H), 126.30 (*C*H), 126.4 (*C*H), 127.55 (*C*H), 127.57 (*C*H), 127.58 (*C*H), 129.1 (*C*H), 131.3 (*C*H), 132.1 (*C*H), 137.9 (*C*), 140.4 (*C*), 140.7 (*C*), 143.5 (*C*), 143.8 (*C*), 144.0 (*C*), 145.9 (*C*), 147.5 (*C*). HRMS-EI (m/z): $[M]^+$ calcd for $C_{38}H_{29}N$, 499.2300; found, 499.2290.

_N_,_N_-Bis(4-methoxyphenyl)-9,10-di(naphthalen-2-yl)anthracen-2-amine (3y)

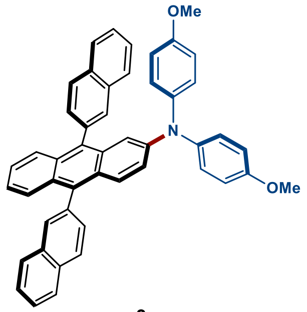

3y

The reaction was carried out with 254.7 mg (0.50 mmol) of **1y** and 114.7 mg (0.50 mmol) of **2d**. The product **3y** was obtained as an orange powder (246.7 mg, 0.375 mmol, 75% yield).

^{1}H NMR (392 MHz, $CDCl_3$, δ): 3.67 (s, 6H), 6.65–6.70 (m, 4H), 6.94–7.00 (m, 5H), 7.08 (dd, J = 2.2, 9.4 Hz, 1H), 7.18–7.28 (m, 2H), 7.48 (d, J = 8.5 Hz, 1H), 7.50–7.67 (m, 7H), 7.71 (d, J = 8.1 Hz, 1H), 7.79–7.85 (m, 2H), 7.86–7.96 (m, 3H), 7.97–8.03 (m, 2H), 8.06 (d, J = 8.5 Hz, 1H). ^{13}C NMR (99 MHz, $CDCl_3$, δ): 55.2 (*C*H_3), 113.0 (*C*H), 114.3 (*C*H), 122.5 (*C*H), 123.9 (*C*H), 125.2 (*C*H), 125.7 (*C*H), 125.9 (*C*H), 126.1 (*C*H), 126.3 (*C*H), 126.4 (*C*H), 126.6 (*C*), 127.1 (*C*H), 127.6 (*C*H), 127.78 (*C*H), 127.83 (*C*H), 127.9 (*C*H), 128.0 (*C*H), 128.8 (*C*), 129.4 (*C*H), 129.6 (*C*H), 130.0 (*C*H), 130.2 (*C*H), 130.4 (*C*), 131.2 (*C*), 132.4 (*C*), 132.6 (*C*), 133.2 (*C*), 133.3 (*C*), 134.1 (*C*), 136.3 (*C*), 136.6 (*C*), 136.7 (*C*), 140.1 (*C*), 145.3 (*C*), 155.8 (*C*). HRMS-ESI (m/z): $[M+Na]^+$ calcd for $C_{48}H_{35}O_2NNa$, 680.2560; found, 680.2572.

_N,N_-Bis(4-methoxyphenyl)-3',4',5',6'-tetraphenyl-(1,1':2',1''-terphenyl)-4-amine (3z)

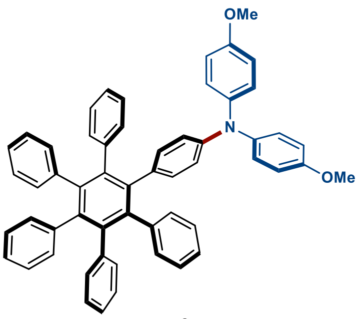

3z

The reaction was carried out with 306.8 mg (0.50 mmol) of **1z** and 114.7 mg (0.50 mmol) of **2d**. The product **3z** was obtained as a white powder (156.2 mg, 0.205 mmol, 41% yield).

^{1}H NMR (392 MHz, $CDCl_3$, δ): 3.75 (s, 6H), 6.48 (d, J = 8.1 Hz, 2H), 6.61 (d, J = 6.6 Hz, 2H), 6.69–6.74 (m, 4H), 6.75–6.79 (m, 4H), 6.80–6.95 (m, 25H). ^{13}C NMR (99 MHz, $CDCl_3$, δ): 55.4 (CH$_3$), 114.3 (CH), 120.9 (CH), 125.0 (CH), 125.1 (CH), 125.3 (CH), 126.5 (CH), 131.4 (CH), 131.5 (CH), 132.0 (CH), 134.0 (C), 140.05 (C), 140.13 (C), 140.3 (C), 140.4 (C), 140.6 (C), 140.8 (C), 141.4 (C), 145.6 (C), 155.0 (C). HRMS-ESI (m/z): [M+Na]$^+$ calcd for $C_{56}H_{43}O_2NNa$, 784.3186; found, 784.3196.

10,20-Bis(3,5-dimethylphenyl)-_N_5,_N_5,_N_15,_N_15-tetrakis(4-methoxyphenyl)porphyrin-5,15-diamine (3aa)

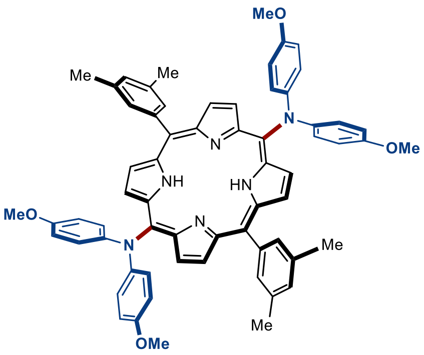

3aa

The reaction was carried out with 135.4 mg (0.20 mmol) of **1aa** and 114.8 mg (0.50 mmol) of **2d**. The product **3aa** was obtained as a dark purple powder (107.0 mg, 0.11 mmol, 55% yield).

^{1}H NMR (392 MHz, $CDCl_3$, δ): 2.54 (s, 12H), 3.71 (s, 12H), 6.73 (d, J = 8.5 Hz, 9H), 7.16–7.27 (m, 11H), 7.71 (s, 4H), 8.66 (d, J = 4.5 Hz, 4H), 9.13 (d, J = 4.9 Hz, 4H). ^{13}C NMR (99 MHz, $CDCl_3$, δ): 21.4 (CH$_3$), 55.4 (CH$_3$), 114.4 (CH), 120.3 (C), 122.6 (C), 123.2 (CH), 129.3 (CH), 132.5 (CH), 135.9 (C), 141.1 (C), 146.8 (C), 153.5 (C). HRMS-ESI (m/z): [M]$^+$ calcd for $C_{64}H_{57}O_4N_6$, 973.4436; found, 973.4460.

10-(Dimesitylboraneyl)-*N*,*N*-diphenylanthracen-9-amine (3ab)

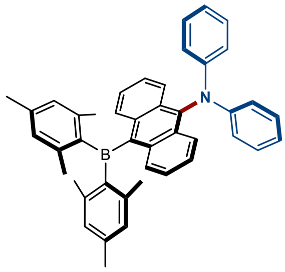

3ab

The reaction was carried out with 252.7 mg (0.50 mmol) of **1ab** and 84.5 mg (0.50 mmol) of **2a**. The product **3ab** was obtained as a yellow powder (184.0 mg, 0.31 mmol, 62% yield).

^{1}H NMR (392 MHz, $CDCl_3$, δ): 1.68 (brs, 6H), 2.19 (brs, 6H), 2.28 (s, 6H), 6.66 (brs, 2H), 6.87 (t, *J* = 7.2 Hz, 4H), 7.06–7.11 (m, 4H), 7.12–7.21 (m, 6H), 7.23–7.31 (m, 2H), 8.08 (d, *J* = 8.5 Hz, 2H), 8.13 (d, *J* = 8.5 Hz, 2H). ^{13}C NMR (99 MHz, $CDCl_3$, δ): 21.3 (CH_3), 23.6 (br, CH_3), 120.0 (*C*H), 121.1 (*C*H), 124.9 (*C*H), 125.1 (*C*H), 126.2 (*C*H), 129.0 (*C*H), 129.2 (*C*H), 130.6 (*C*), 135.0 (*C*), 139.3 (*C*), 139.9 (*C*), 145.3 (br, *C*-B), 147.5 (*C*). HRMS-EI (*m*/*z*): [M]$^+$ calcd for $C_{44}H_{40}BN$, 592.3290; found, 592.3283.

***N*,*N*,9-Triphenyl-9H-carbazol-3-amine (3ac)**

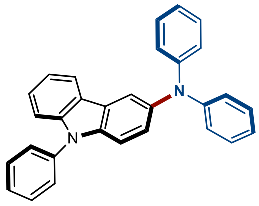

3ac

The reaction was carried out with 161.1 mg (0.50 mmol) of **1ac** and 84.6 mg (0.50 mmol) of **2a**. The product **3ac** was obtained as a yellow powder (168.3 mg, 0.41 mmol, 82% yield).

^{1}H NMR (392 MHz, $CDCl_3$, δ): 6.95 (t, *J* = 7.2 Hz, 2H), 7.12 (d, *J* = 8.1 Hz, 4H), 7.18–7.28 (m, 6H), 7.34 (d, *J* = 9.0 Hz, 1H), 7.37–7.42 (m, 2H), 7.43–7.49 (m, 1H), 7.55–7.64 (m, 4H), 7.93 (brs, 1H), 7.99 (d, *J* = 8.1 Hz, 1H). ^{13}C NMR (99 MHz, $CDCl_3$, δ): 109.9 (*C*H), 110.7 (*C*H), 118.5 (*C*H), 119.9 (*C*H), 120.5 (*C*H), 121.6 (*C*H), 122.7 (*C*H), 123.0 (*C*), 124.3 (*C*), 125.7 (*C*H), 126.1 (*C*H), 127.0 (*C*H), 127.4 (*C*H), 129.0 (*C*H), 129.9 (*C*H), 137.6 (*C*), 138.0 (*C*), 140.5 (*C*), 141.3 (*C*), 148.6 (*C*). HRMS-EI (*m*/*z*): [M]$^+$ calcd for $C_{30}H_{22}N_2$, 410.1783; found, 410.1783.

_N,N_,5-Triphenylthiophen-2-amine (3ad)

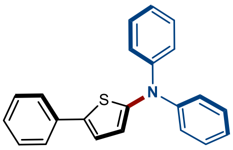

3ad

The reaction was carried out with 119.6 mg (0.50 mmol) of **1ad** and 84.6 mg (0.50 mmol) of **2a**. The product **3ad** was obtained as a white powder (90.0 mg, 0.275 mmol, 55% yield). ^{1}H and ^{13}C NMR were in agreement with the literature.[15]

^{1}H NMR (392 MHz, $CDCl_3$, δ): 6.67 (d, J = 4.0 Hz, 1H), 7.04 (t, J = 7.2 Hz, 2H), 7.11 (d, J = 3.6 Hz, 1H), 7.15–7.21 (m, 4H), 7.22–7.37 (m, 7H), 7.52 (d, J = 7.6 Hz, 2H). ^{13}C NMR (99 MHz, $CDCl_3$, δ): 121.6 (*C*H), 121.8 (*C*H_3), 122.6 (*C*H), 123.0 (*C*H), 125.1 (*C*H), 127.0 (*C*H), 128.8 (*C*H), 129.2 (*C*H), 134.4 (*C*), 138.1 (*C*), 147.7 (*C*), 150.8 (*C*). HRMS-EI (*m*/*z*): $[M]^+$ calcd for $C_{22}H_{17}NS$, 327.1082; found, 327.1078.

2-(Diphenylamino)anthracene-9,10-dione (3ae)

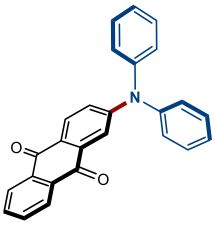

3ae

The reaction was carried out with 143.6 mg (0.50 mmol) of **1ae** and 84.6 mg (0.50 mmol) of **2a**. The product **3ae** was obtained as an organe powder (107.0 mg, 0.285 mmol, 57% yield).

^{1}H NMR (392 MHz, $CDCl_3$, δ): 7.16–7.23 (m, 6H), 7.26 (dd, J = 2.2, 8.5 Hz, 1H), 7.33–7.41 (m, 4H), 7.70–7.80 (m, 3H), 8.12 (d, J = 8.5 Hz, 1H), 8.22 (dd, J = 1.8, 7.2 Hz, 1H), 8.27–8.31 (m, 1H). ^{13}C NMR (99 MHz, $CDCl_3$, δ): 116.5 (*C*H), 123.8 (*C*H), 125.5 (*C*H), 125.8 (*C*), 126.3 (*C*H), 127.0 (*C*H), 129.2 (*C*H), 129.9 (*C*H), 133.4 (*C*H), 134.6 (*C*), 134.0 (*C*H), 134.9 (*C*), 145.8 (*C*), 153.1 (*C*), 181.7 (*C*), 183.5 (*C*). HRMS-EI (*m*/*z*): $[M]^+$ calcd for $C_{26}H_{17}NO_2$, 375.1259; found, 375.1256.

N1,N1,N3,N3,N6,N6,N8,N8-octakis(4-methoxyphenyl)pyrene-1,3,6,8-tetraamine (3af)

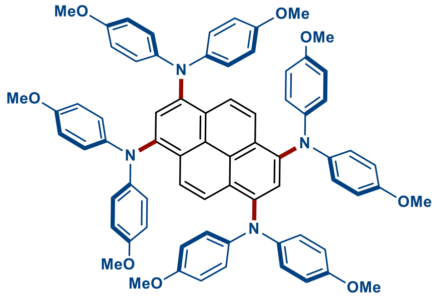

3af

The reaction was carried out with 258.9 mg (0.50 mmol) of **1af** and 596.2 mg (2.6 mmol) of **2d**. The product **3af** was obtained as an organge powder (494.5 mg, 0.445 mmol, 89% yield). ^{1}H and ^{13}C NMR were in agreement with the literature.[16]

^{1}H NMR (392 MHz, $CDCl_3$, δ): 3.737 (s, 12H), 3.740 (s, 12H), 6.66–6.75 (m, 16H), 6.84–6.94 (m, 16H), 7.45 (s, 1H), 7.46 (s, 1H), 7.90 (s, 2H), 7.91 (s, 2H). ^{13}C NMR (99 MHz, $CDCl_3$, δ): 55.4 (CH_3), 114.4 (*C*H), 122.8 (*C*H), 123.1 (*C*H), 126.1 (*C*H), 128.4 (*C*), 128.9 (*C*), 142.4 (*C*), 142.5 (*C*), 154.4 (*C*). HRMS-ESI (*m*/*z*): $[M+Na]^+$ calcd for $C_{72}H_{62}O_8N_4Na$, 1133.4460; found, 1133.4471.

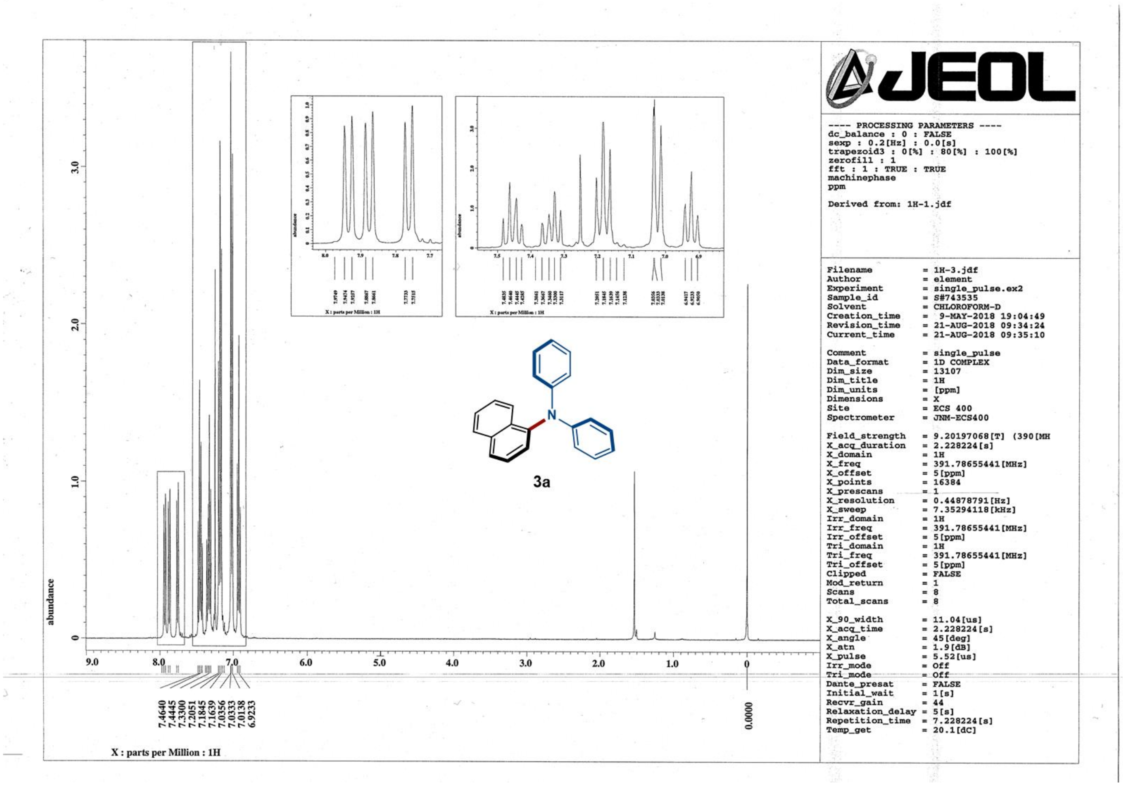

Supplementary Figure 12. ^{1}H NMR spectrum of **3a**.

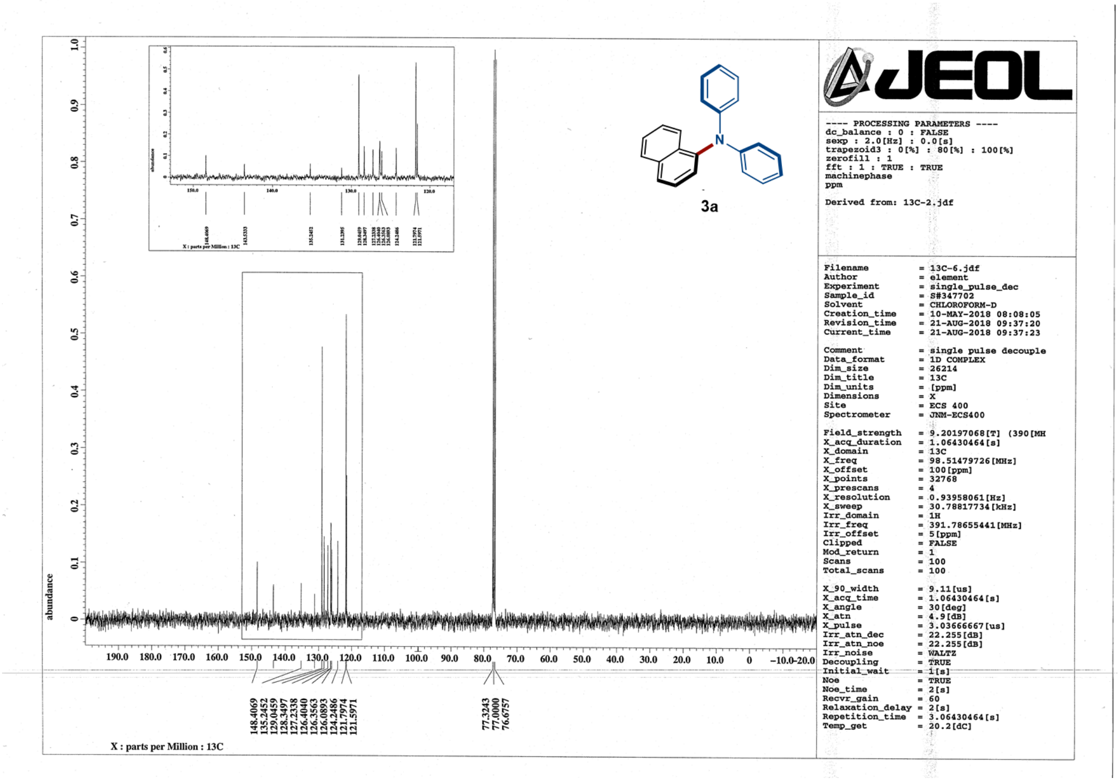

Supplementary Figure 13. ^{13}C NMR spectrum of **3a**.

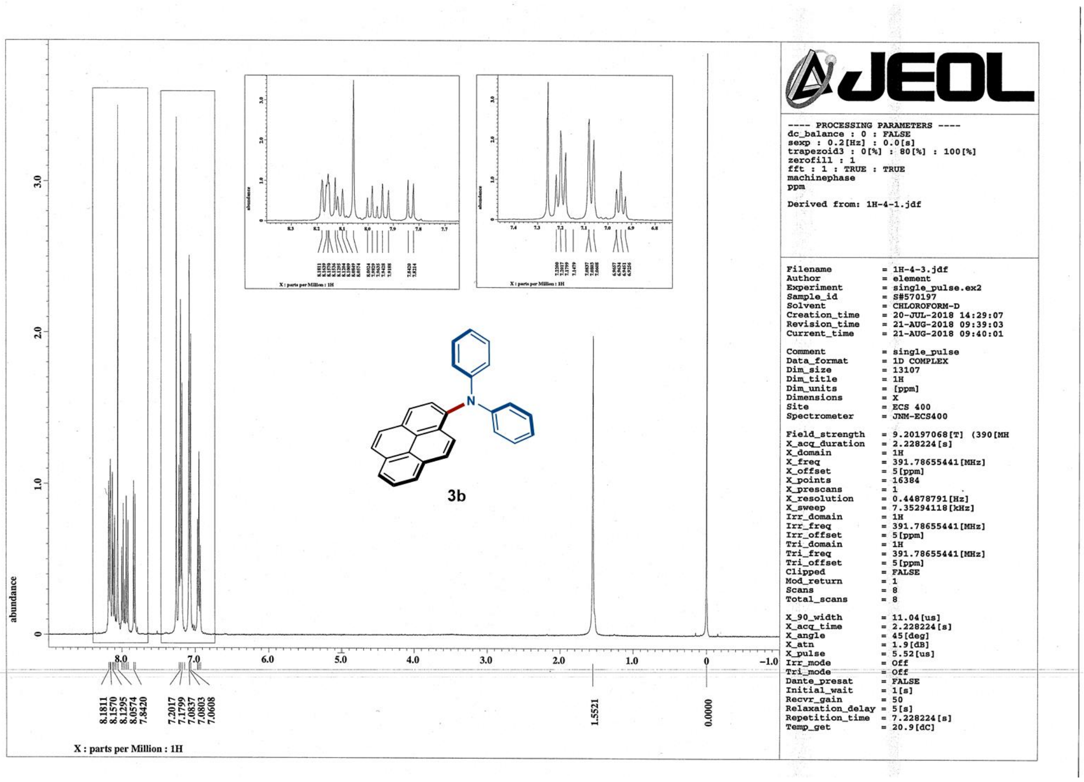

Supplementary Figure 14. ^{1}H NMR spectrum of **3b**.

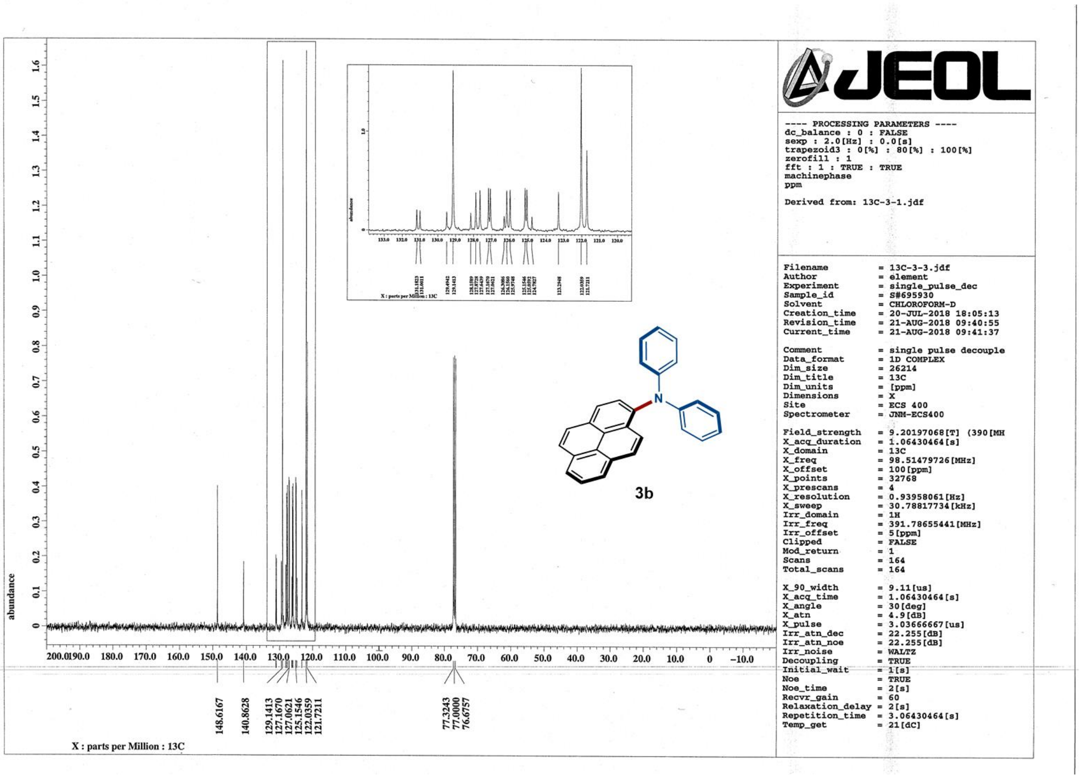

Supplementary Figure 15. ^{13}C NMR spectrum of **3b**.

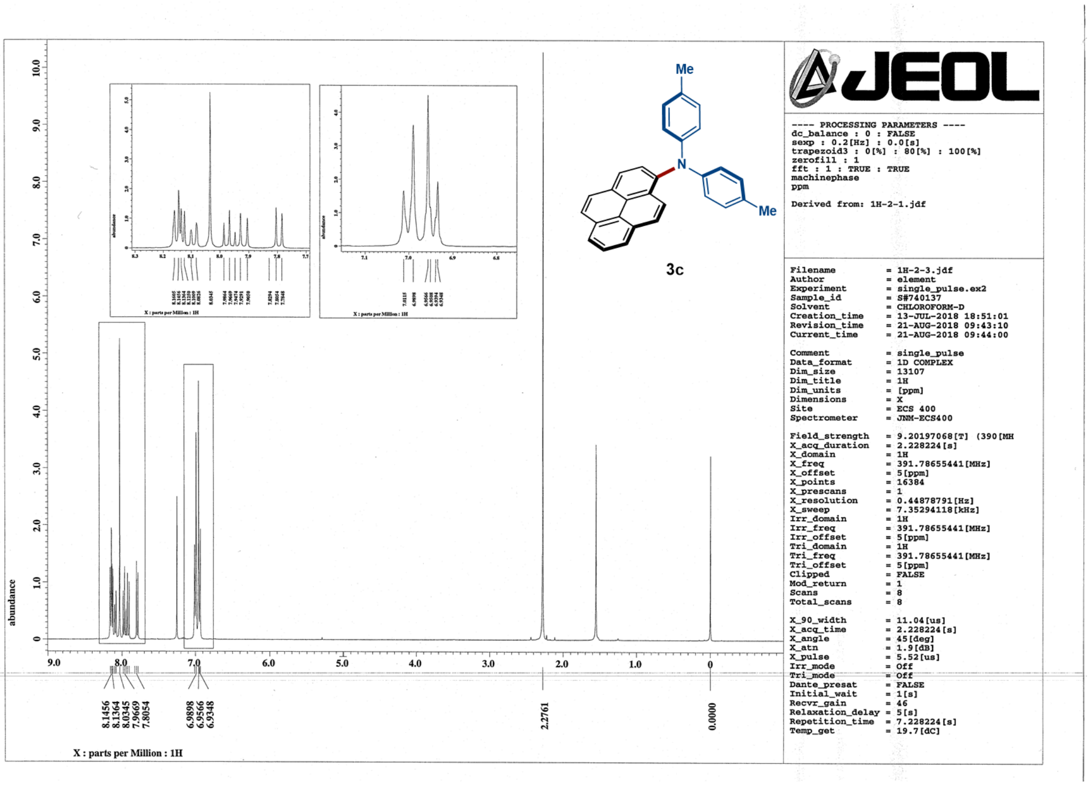

Supplementary Figure 16. ^{1}H NMR spectrum of **3c**.

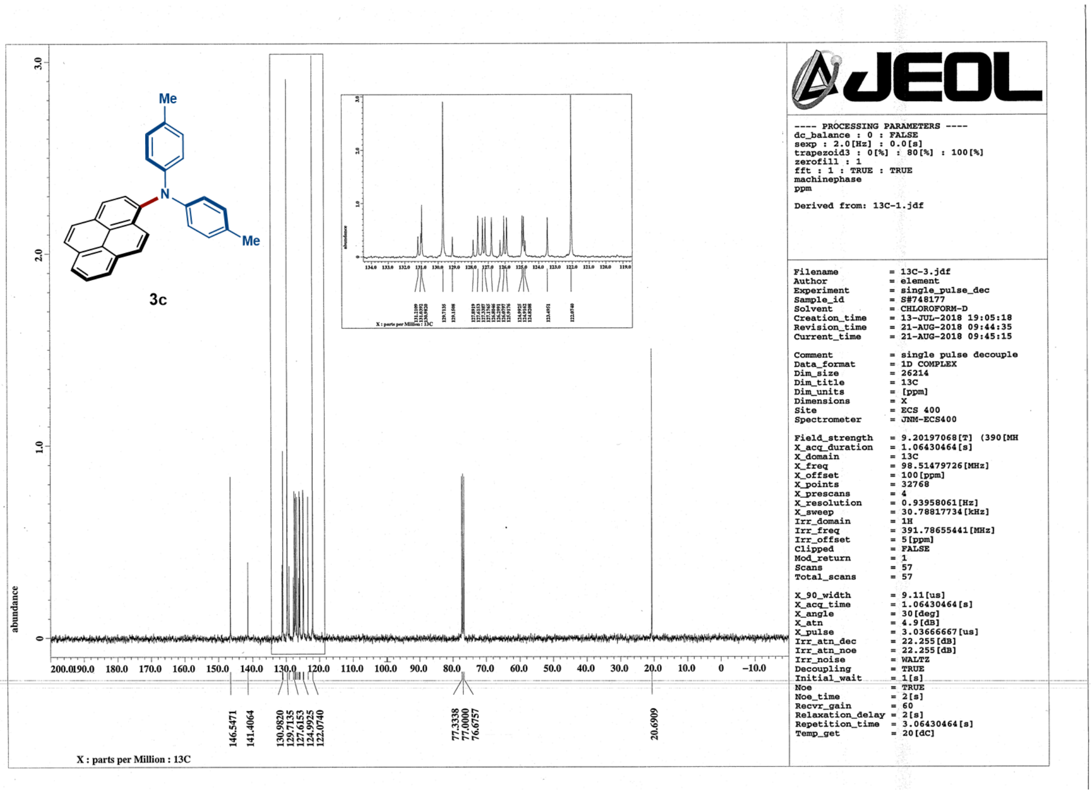

Supplementary Figure 17. ^{13}C NMR spectrum of **3c**.

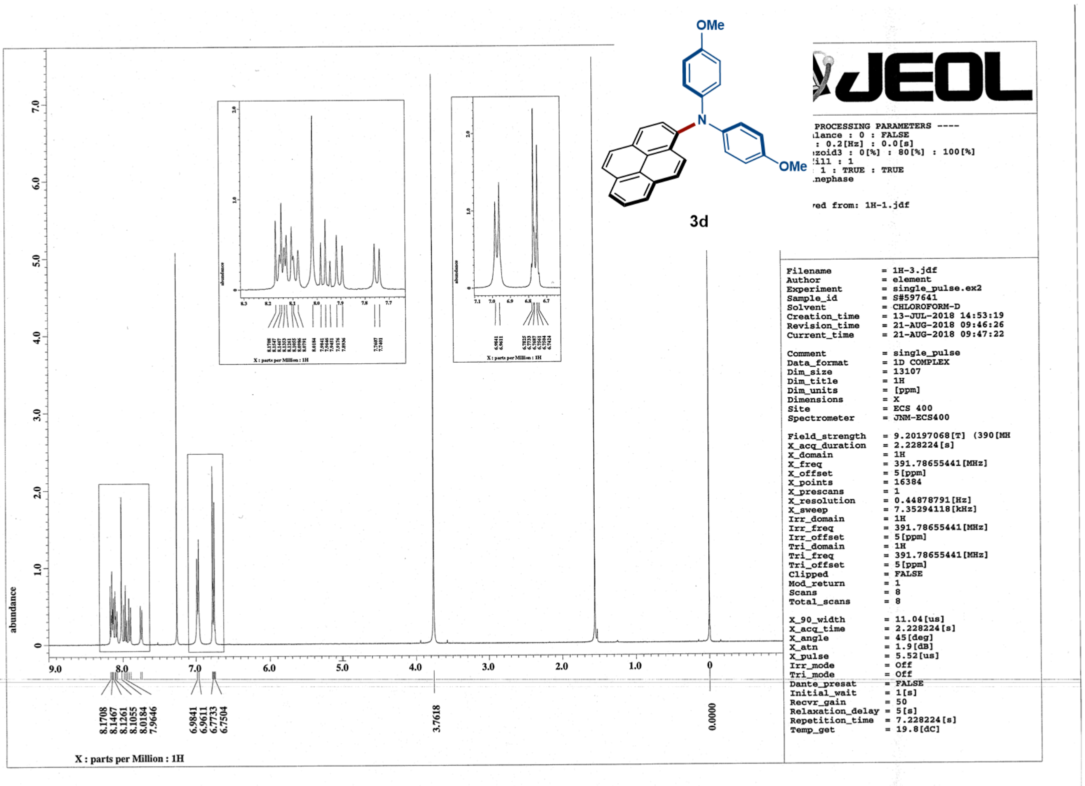

Supplementary Figure 18. ^{1}H NMR spectrum of **3d**.

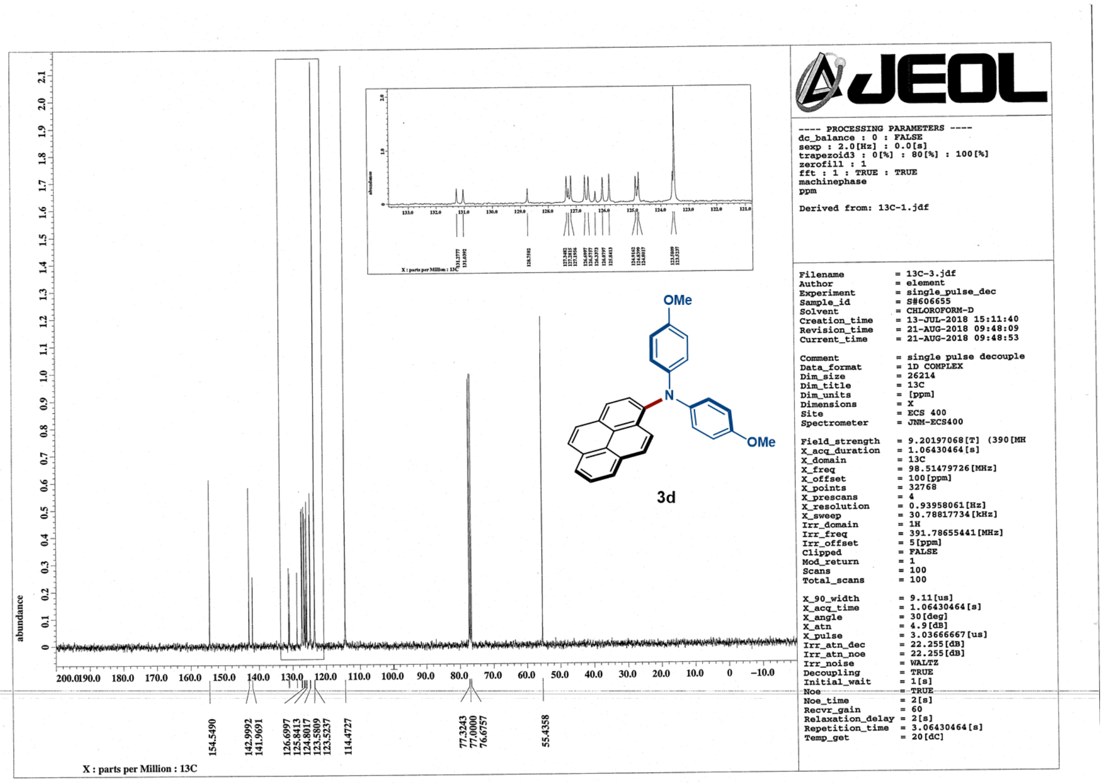

Supplementary Figure 19. ^{13}C NMR spectrum of **3d**.

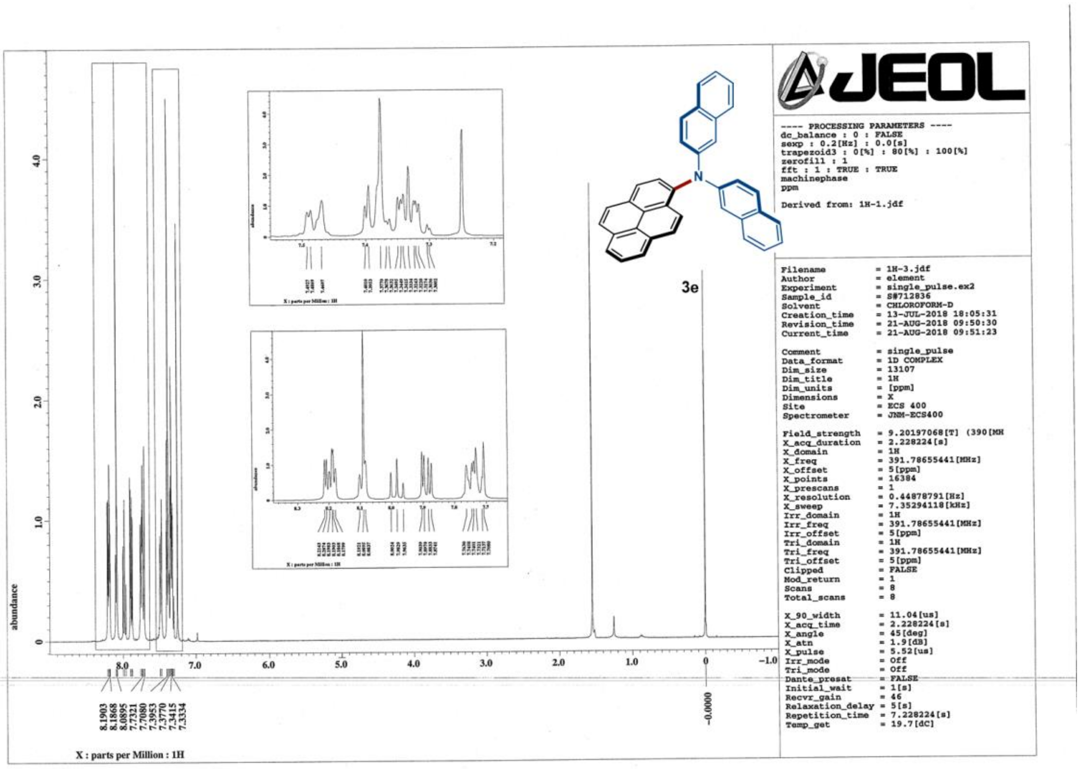

Supplementary Figure 20. ^{1}H NMR spectrum of **3e**.

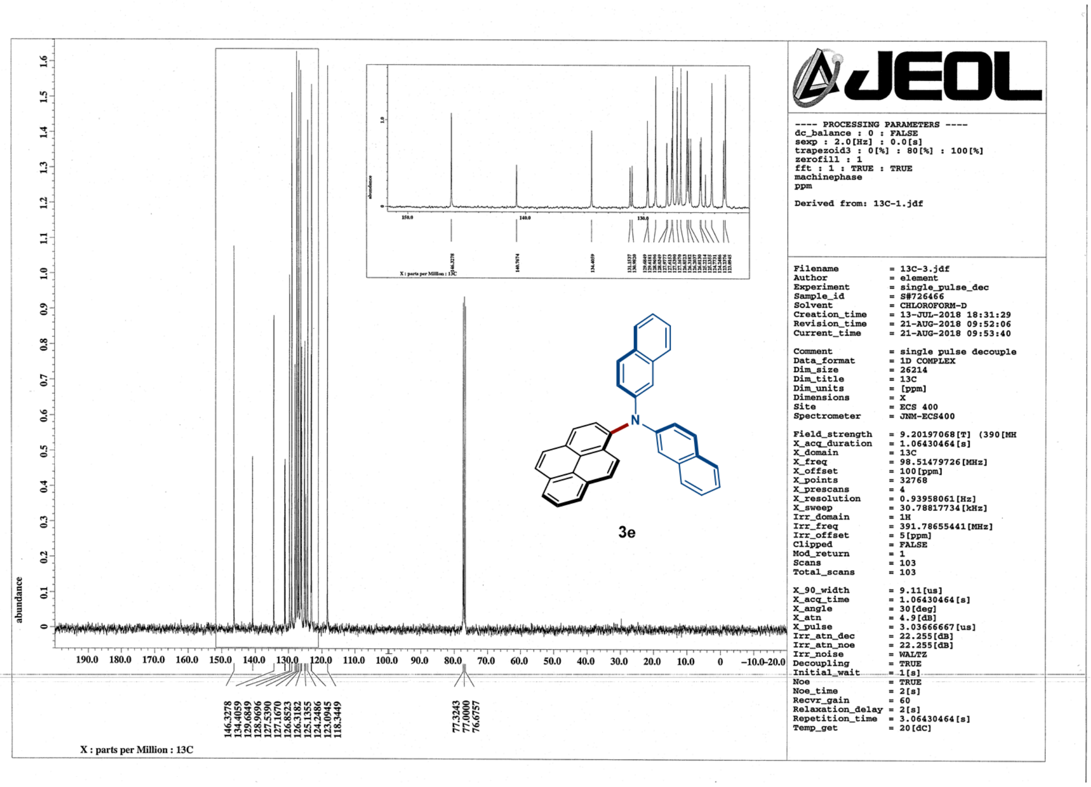

Supplementary Figure 21. ^{13}C NMR spectrum of **3e**.

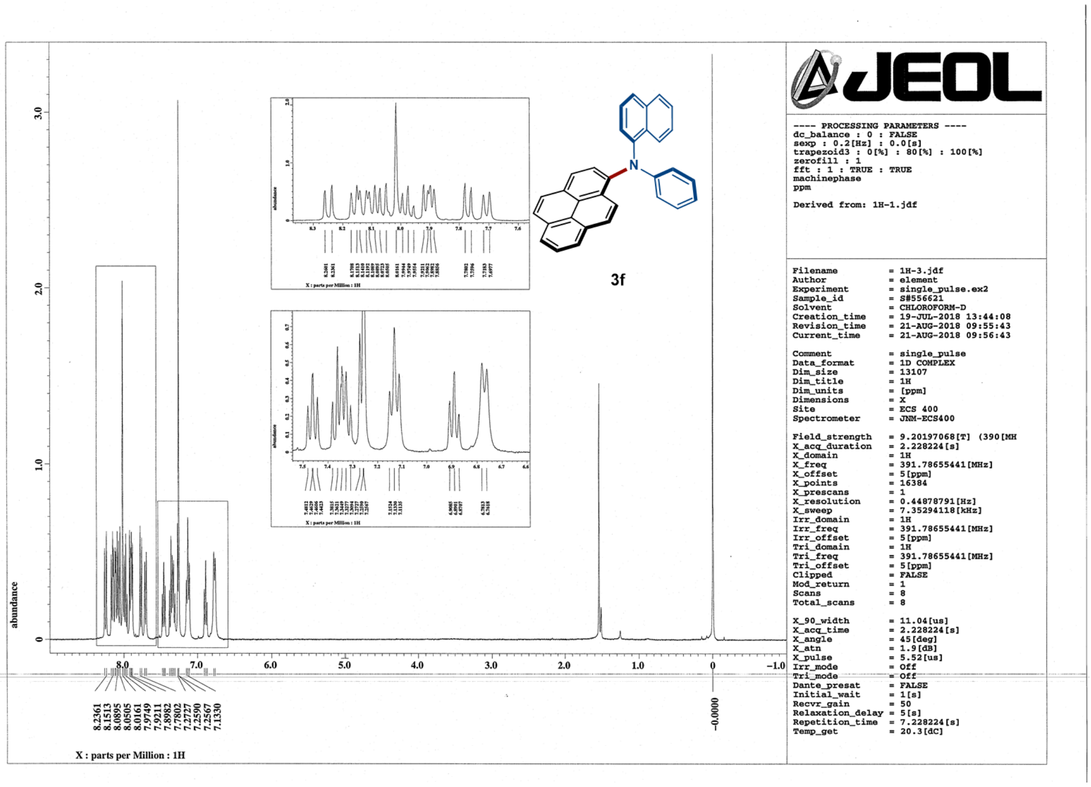

Supplementary Figure 22. ^{1}H NMR spectrum of **3f**.

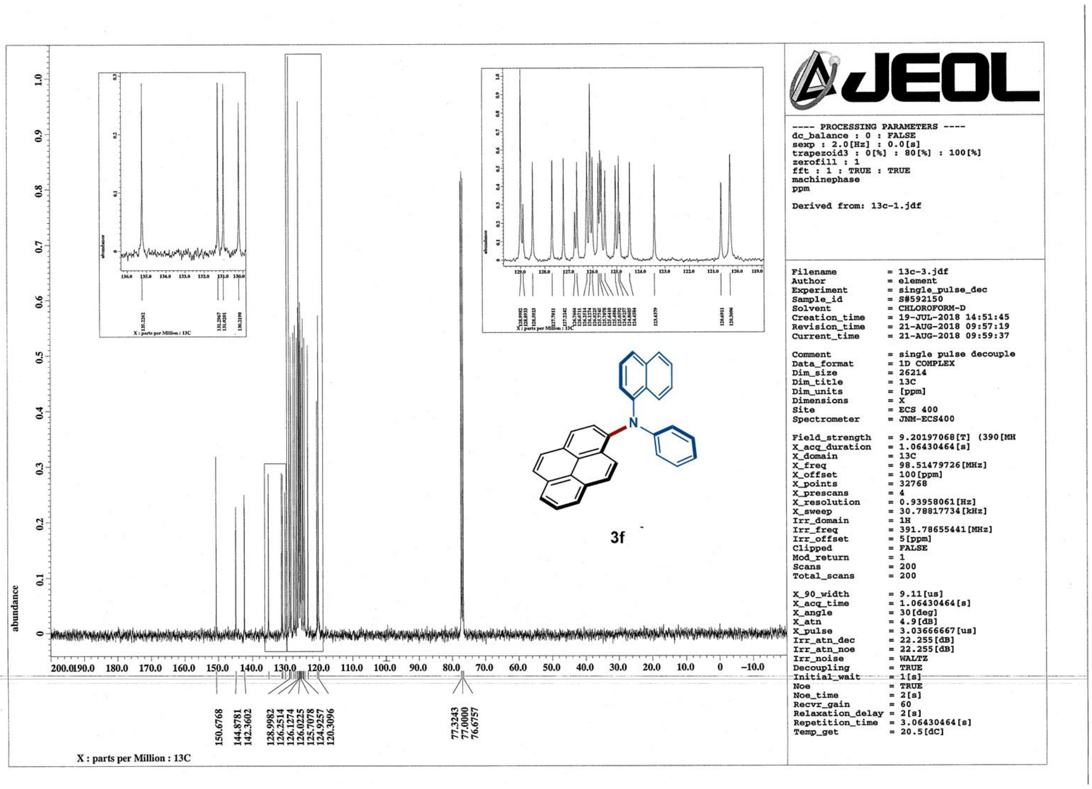

Supplementary Figure 23. ^{13}C NMR spectrum of **3f**.

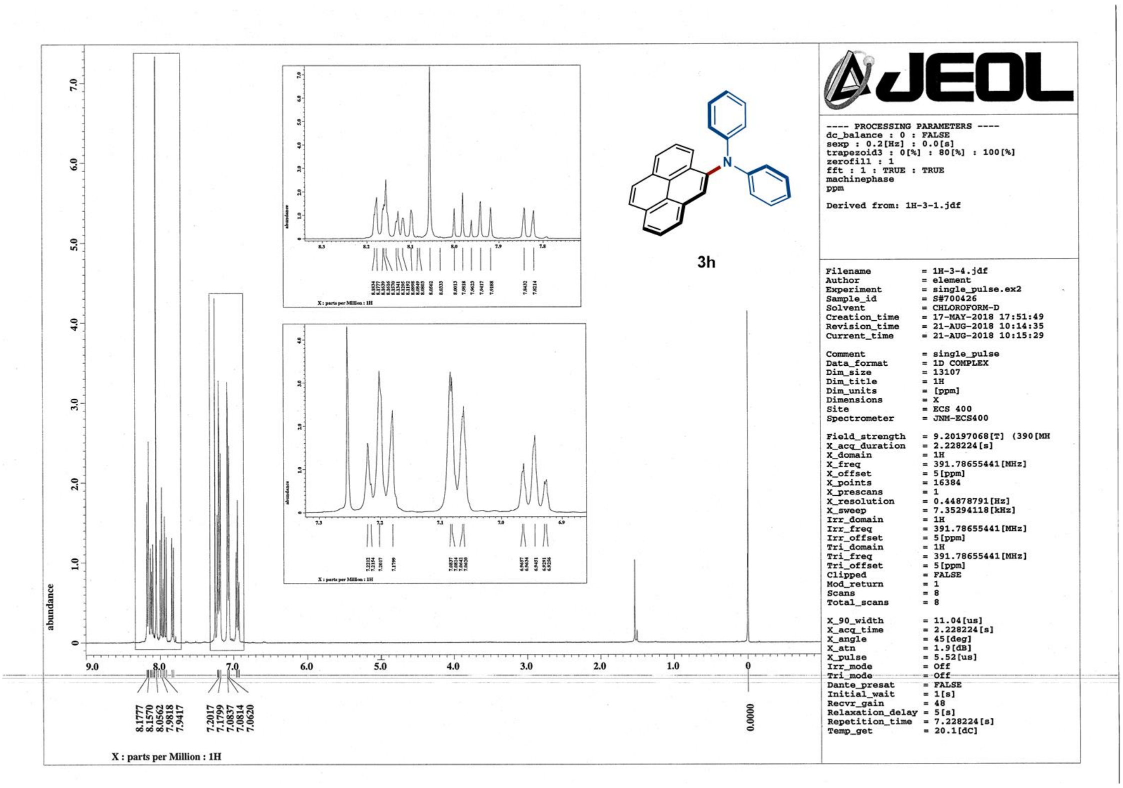

Supplementary Figure 24. ^{1}H NMR spectrum of **3h**.

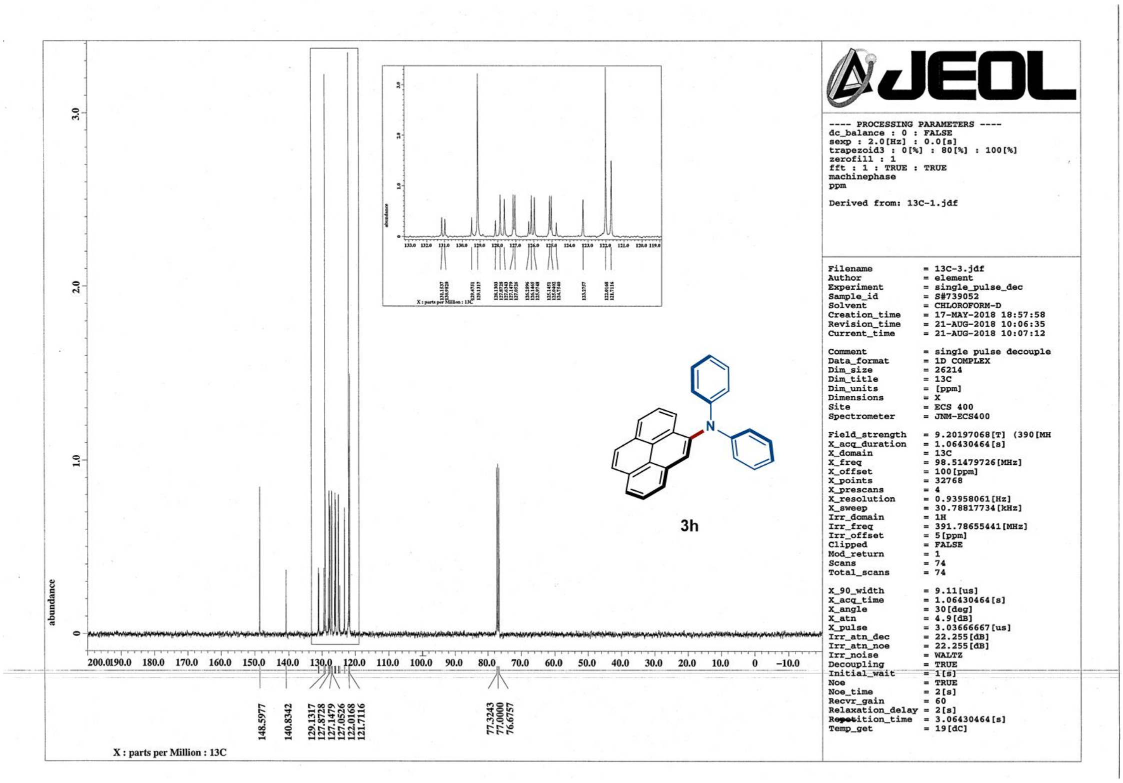

Supplementary Figure 25. ^{13}C NMR spectrum of **3h**.

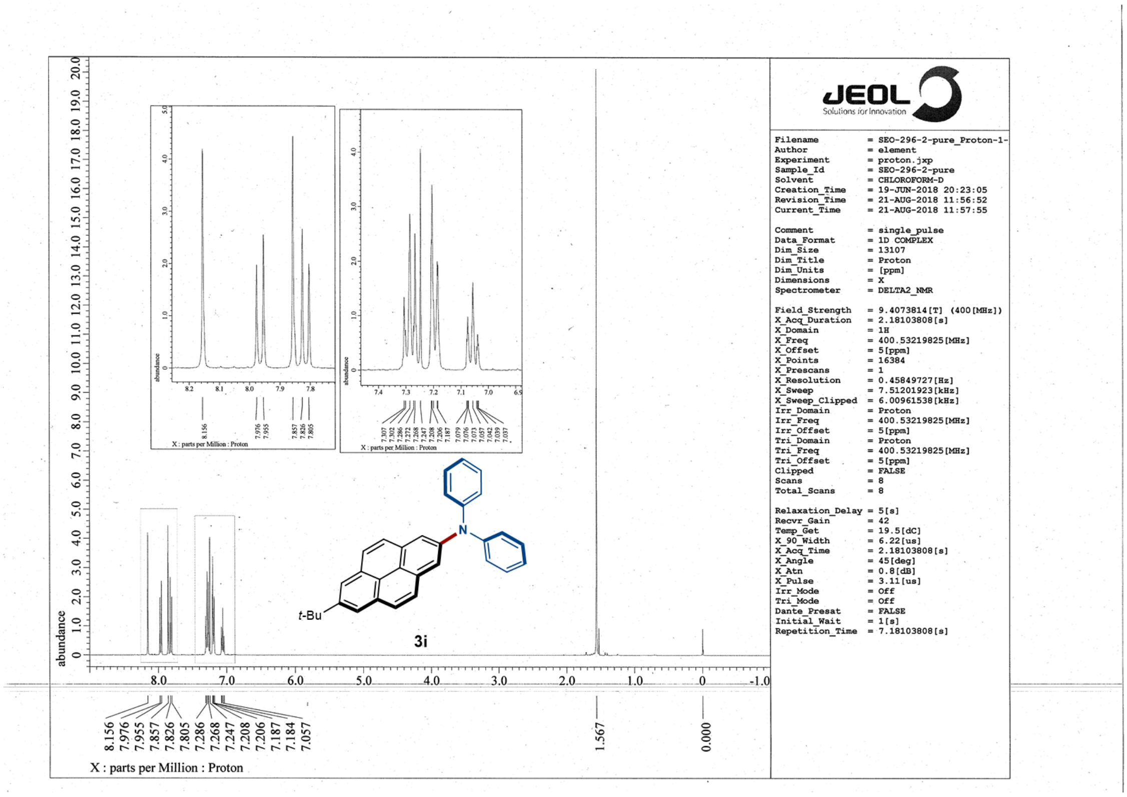

Supplementary Figure 26. ^{1}H NMR spectrum of **3i**.

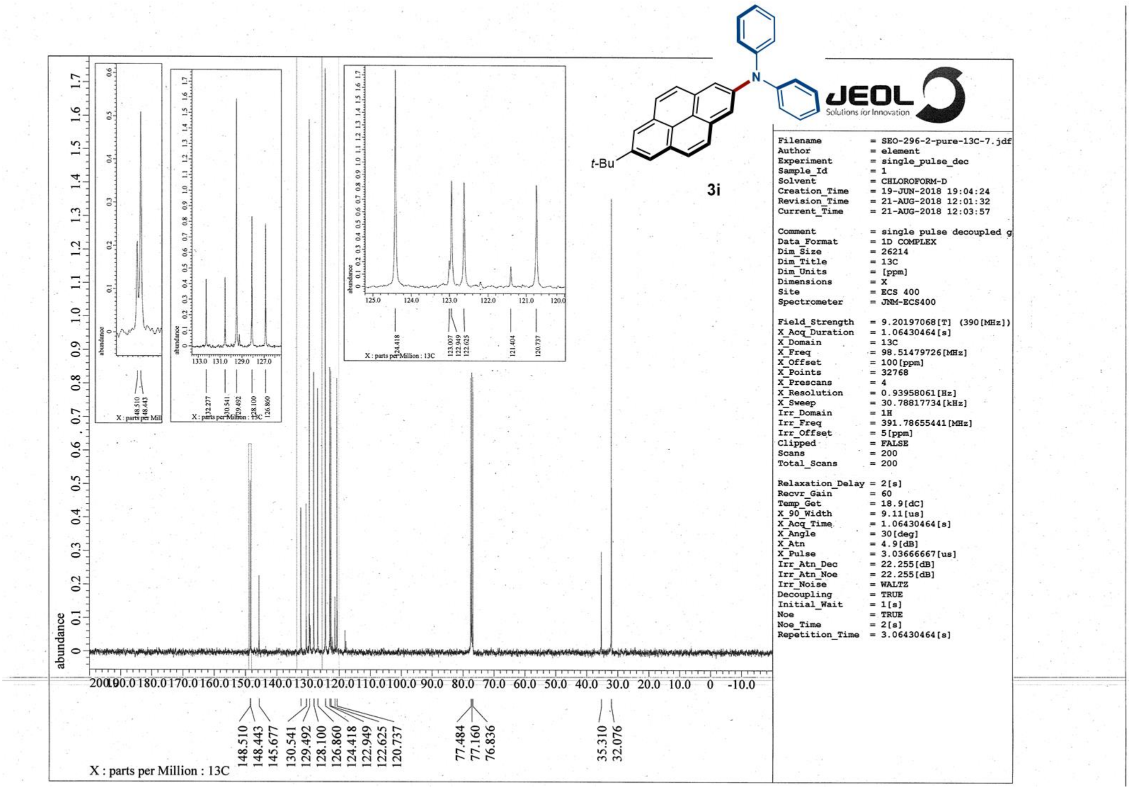

Supplementary Figure 27. ^{13}C NMR spectrum of **3i**.

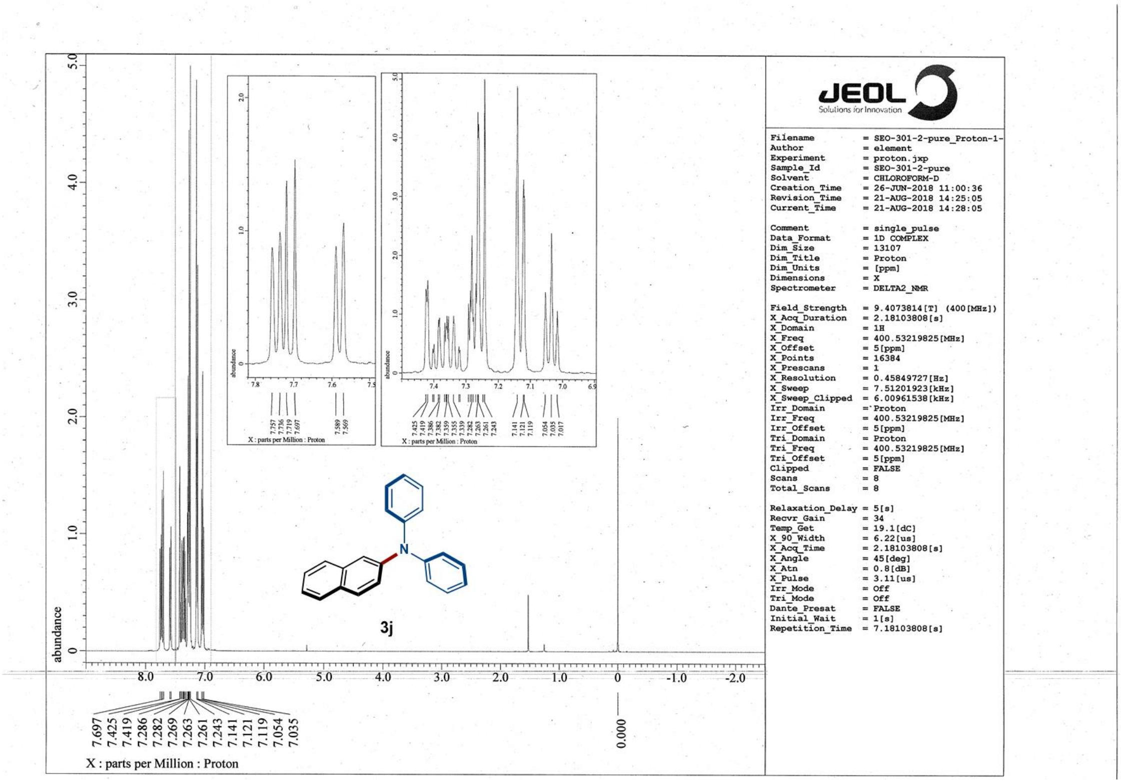

Supplementary Figure 28. ^{1}H NMR spectrum of **3j**.

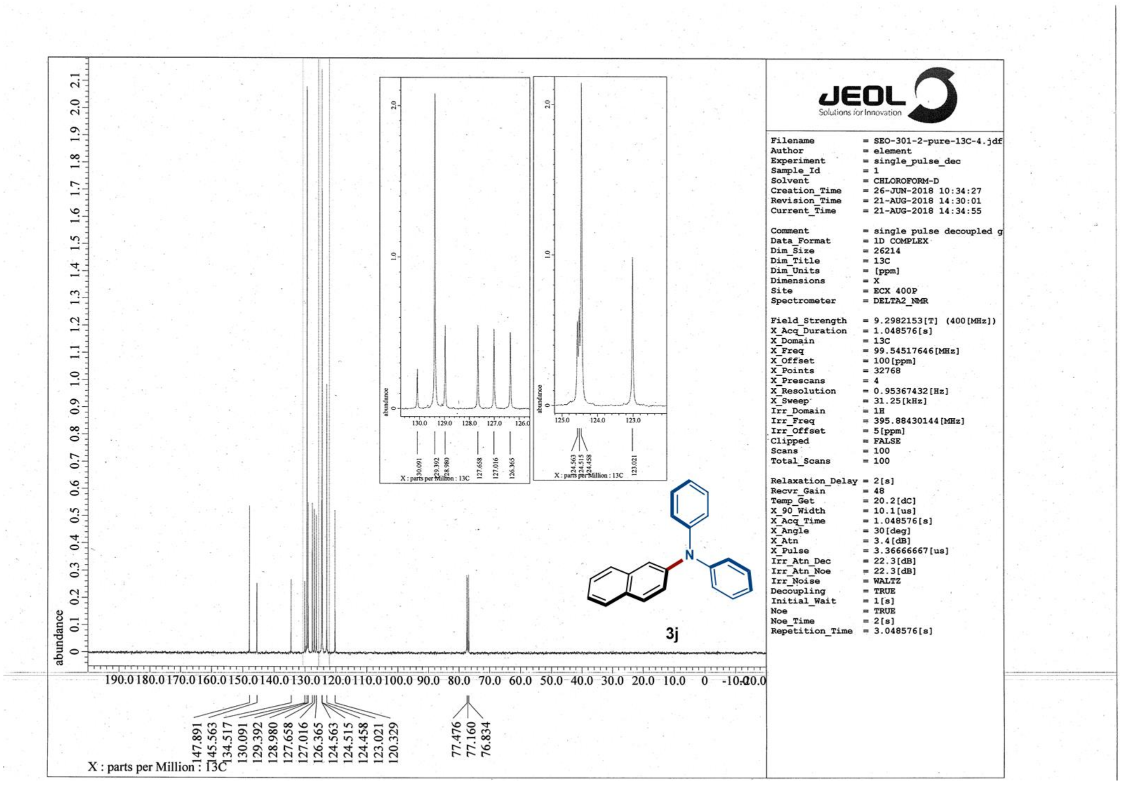

Supplementary Figure 29. ^{13}C NMR spectrum of **3j**.

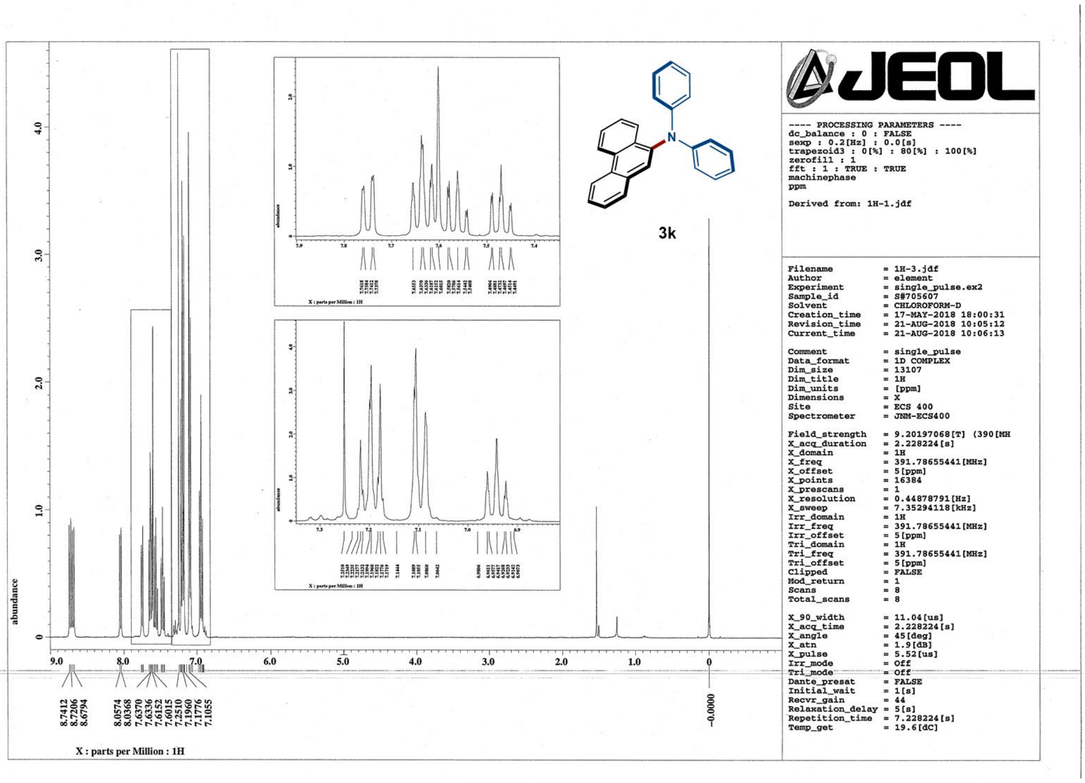

Supplementary Figure 30. ^{1}H NMR spectrum of **3k**.

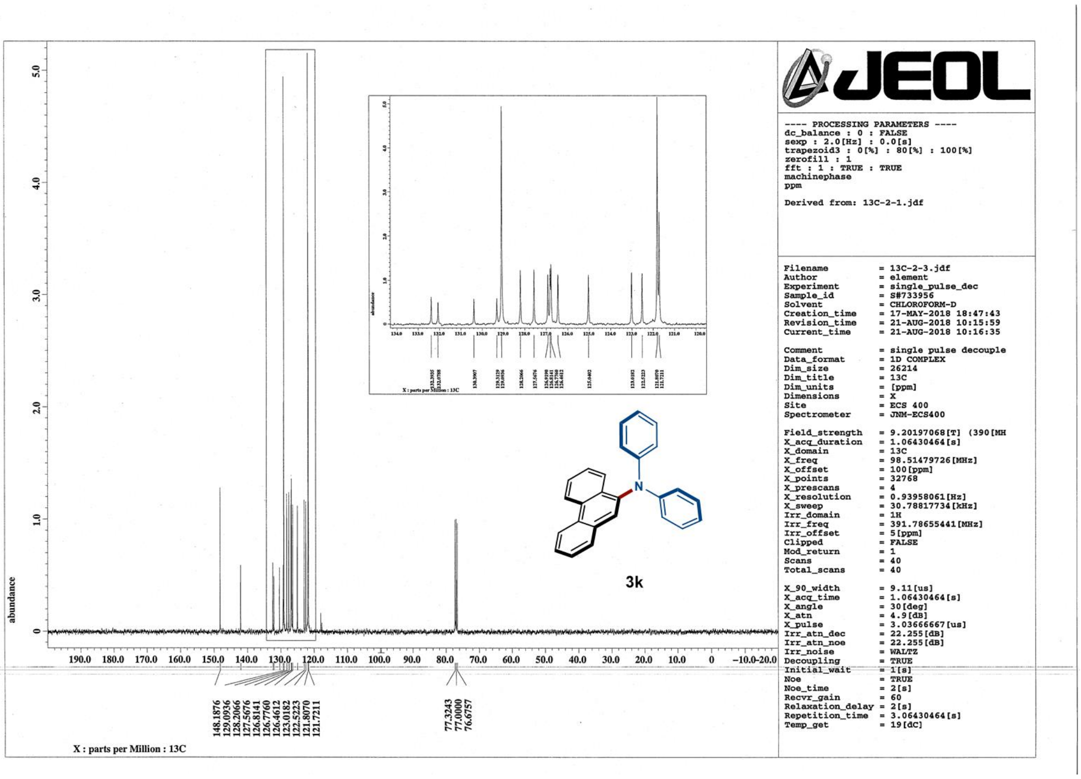

Supplementary Figure 31. ^{13}C NMR spectrum of **3k**.

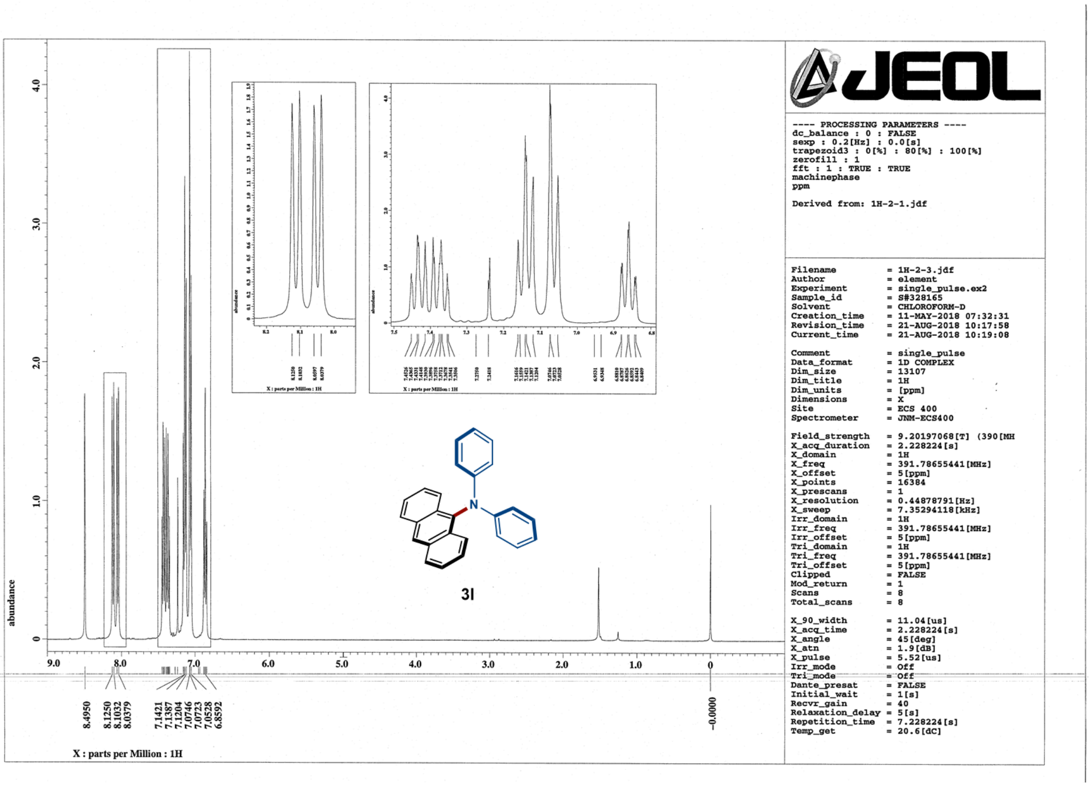

Supplementary Figure 32. ^{1}H NMR spectrum of **3l**.

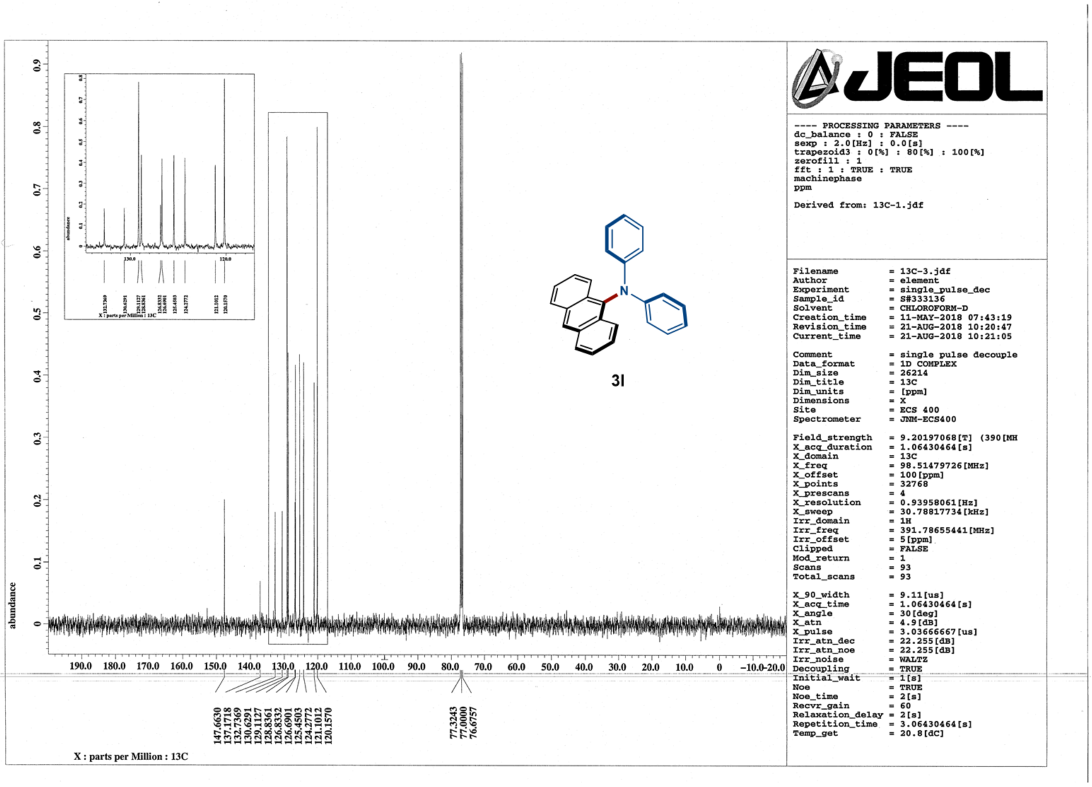

Supplementary Figure 33. ^{13}C NMR spectrum of **3l**.

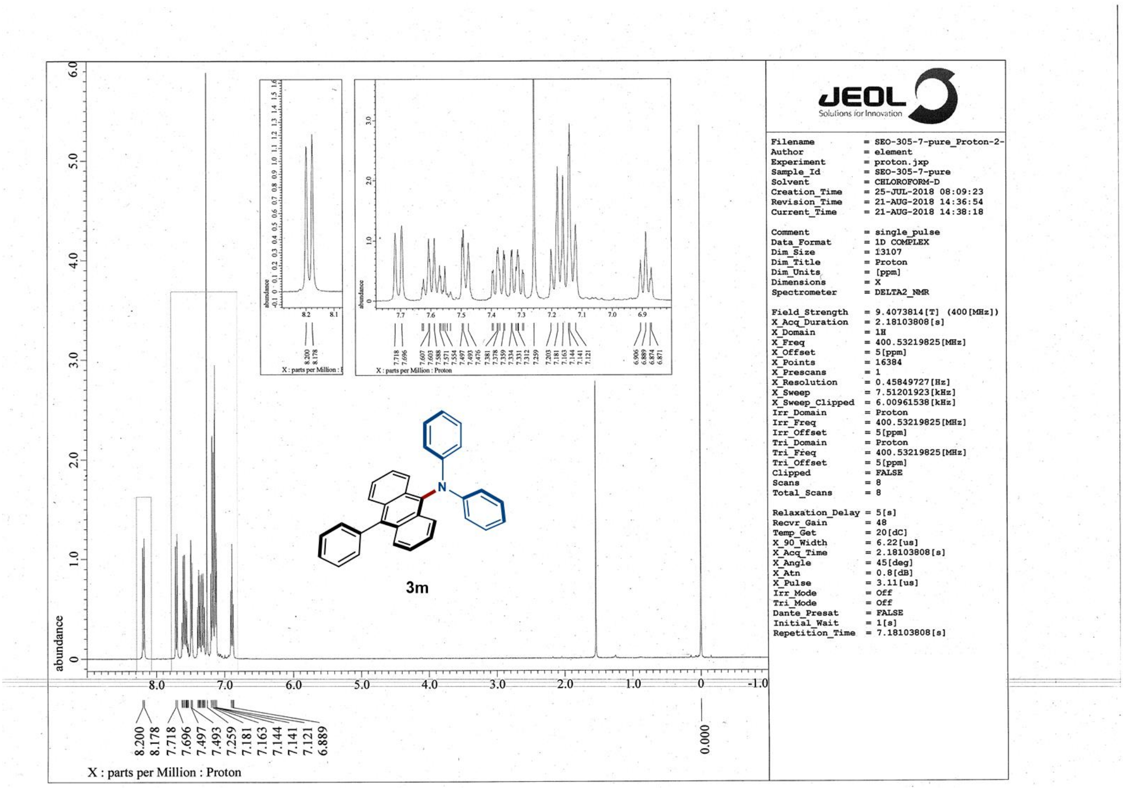

Supplementary Figure 34. ^{1}H NMR spectrum of **3m**.

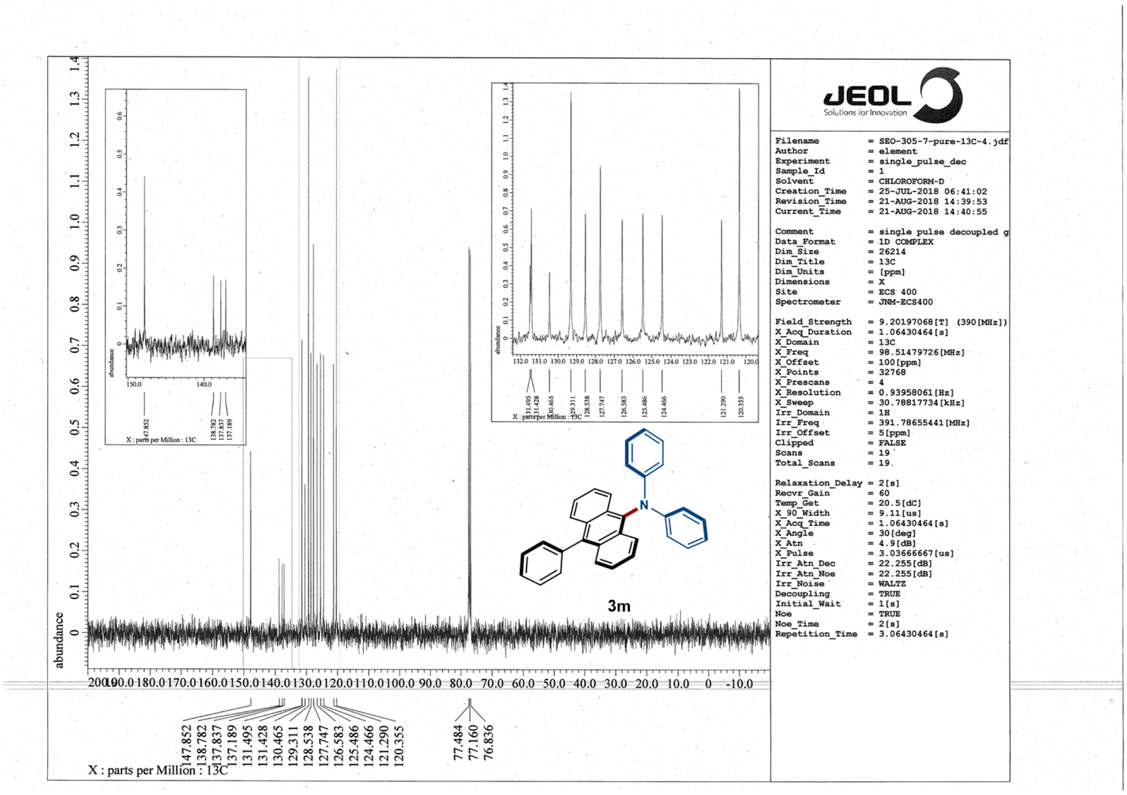

Supplementary Figure 35. ^{13}C NMR spectrum of **3m**.

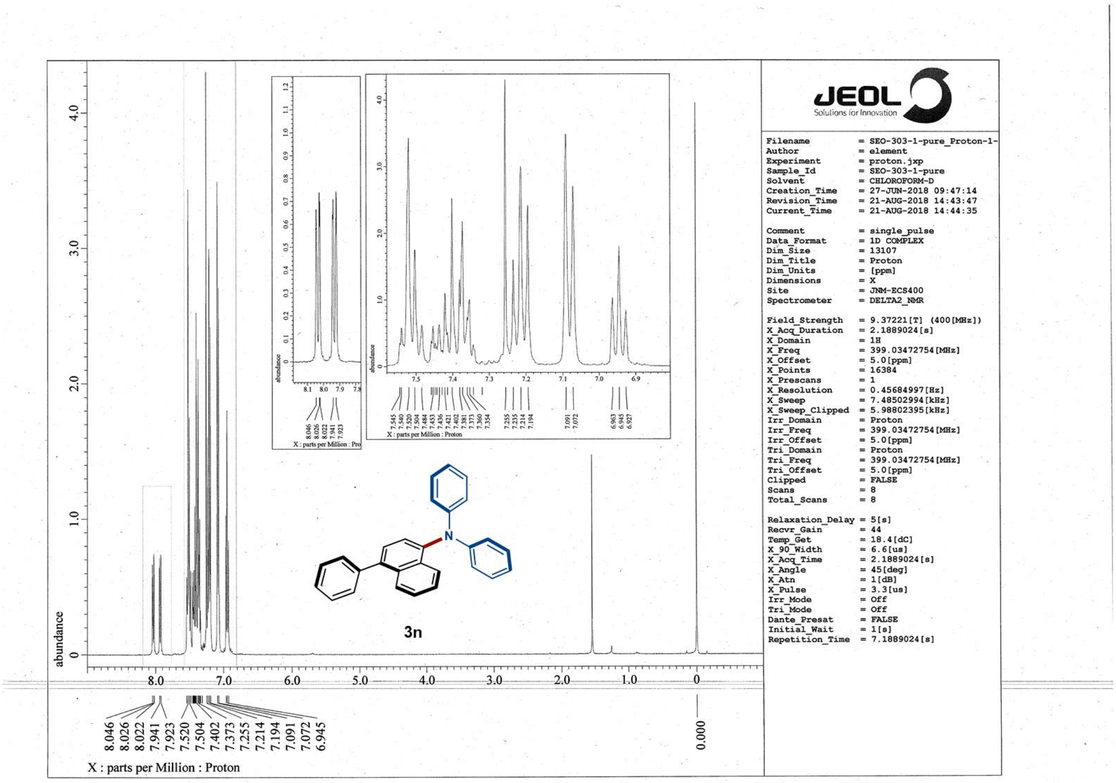

Supplementary Figure 36. ^{1}H NMR spectrum of **3n**.

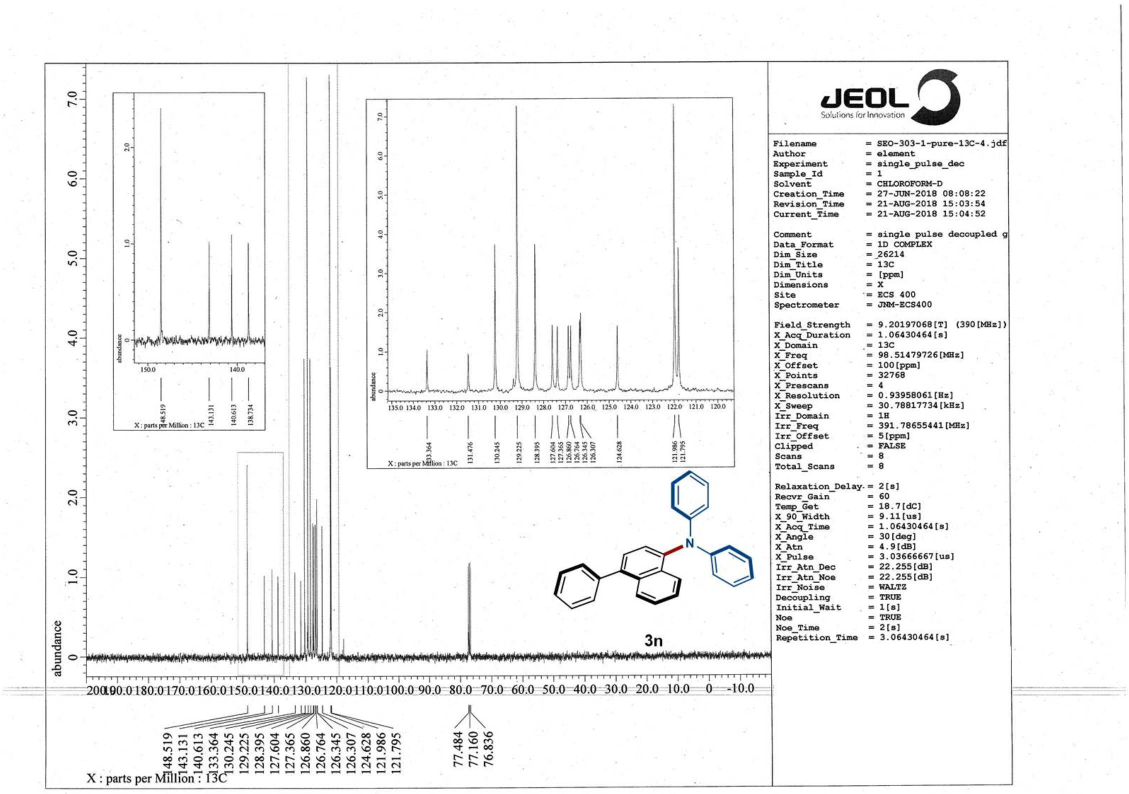

Supplementary Figure 37. ^{13}C NMR spectrum of **3n**.

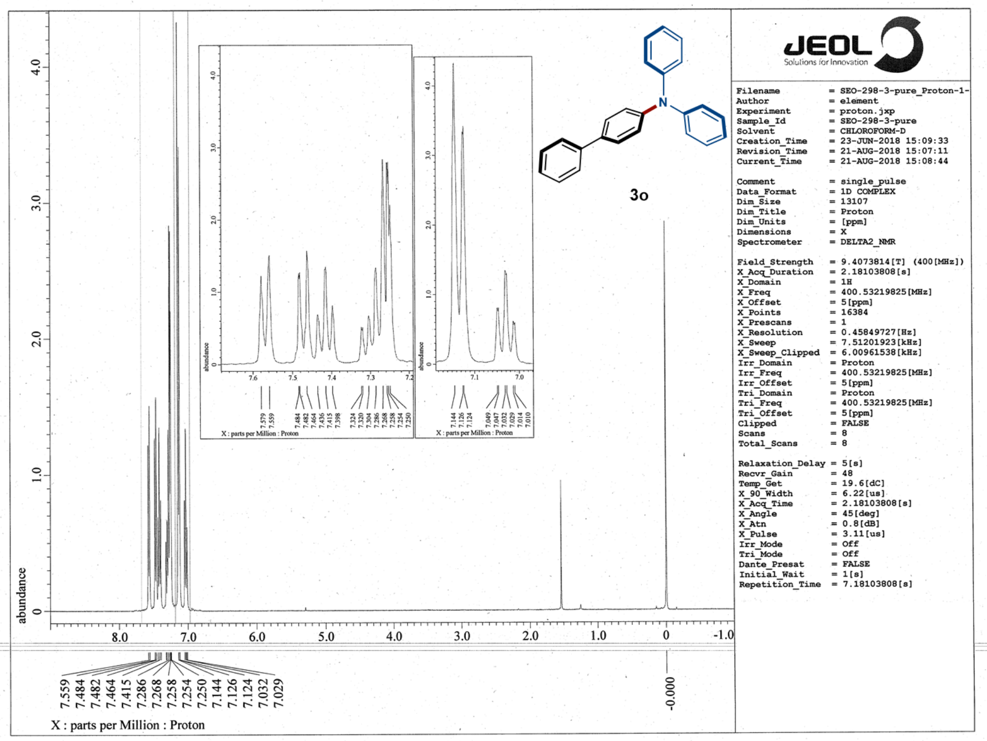

Supplementary Figure 38. ^{1}H NMR spectrum of **3o**.

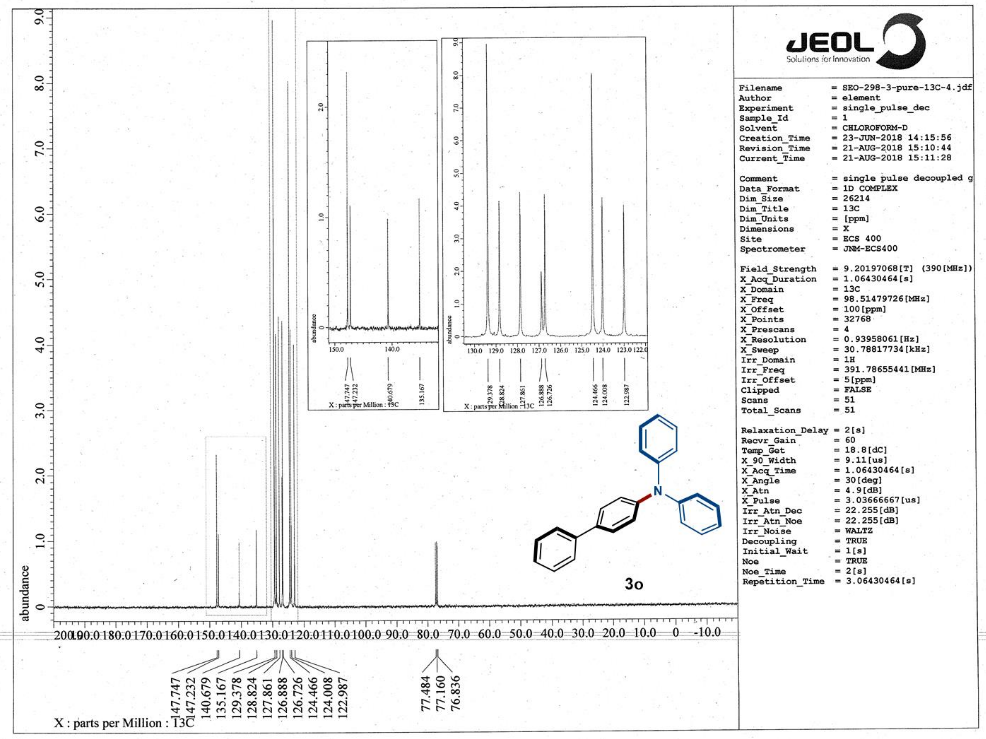

Supplementary Figure 39. ^{13}C NMR spectrum of **3o**.

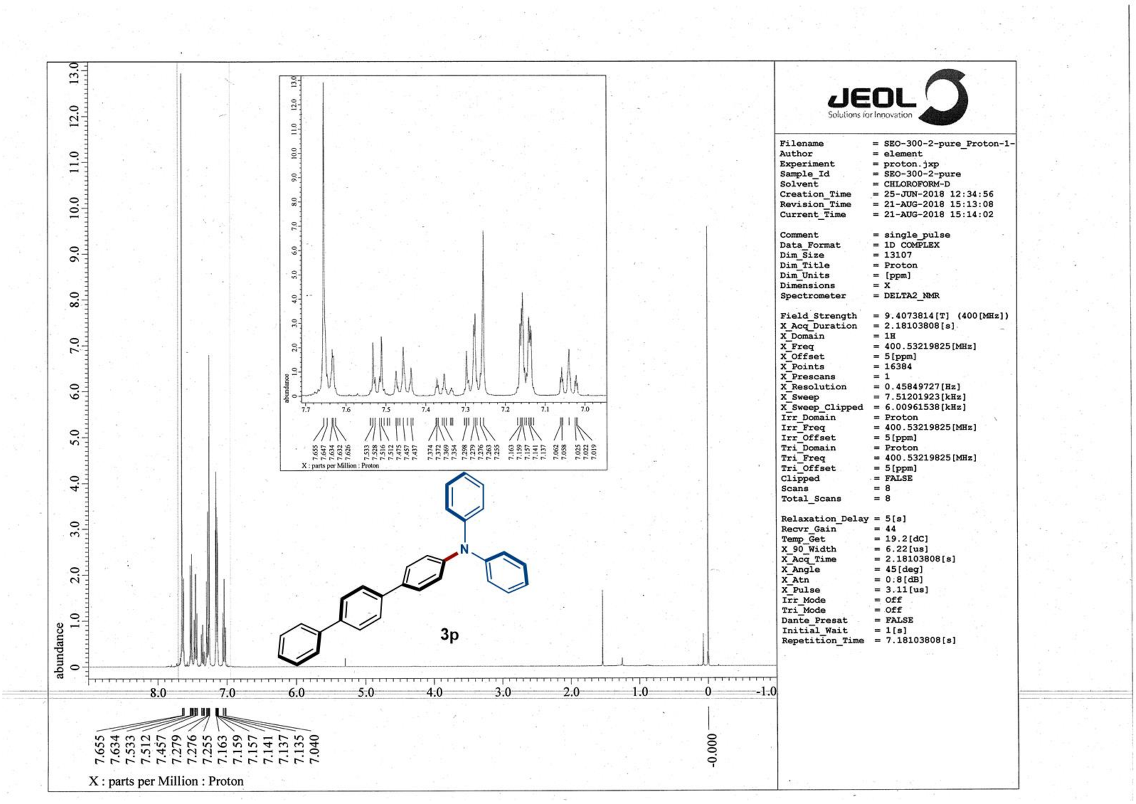

Supplementary Figure 40. ^{1}H NMR spectrum of **3p**.

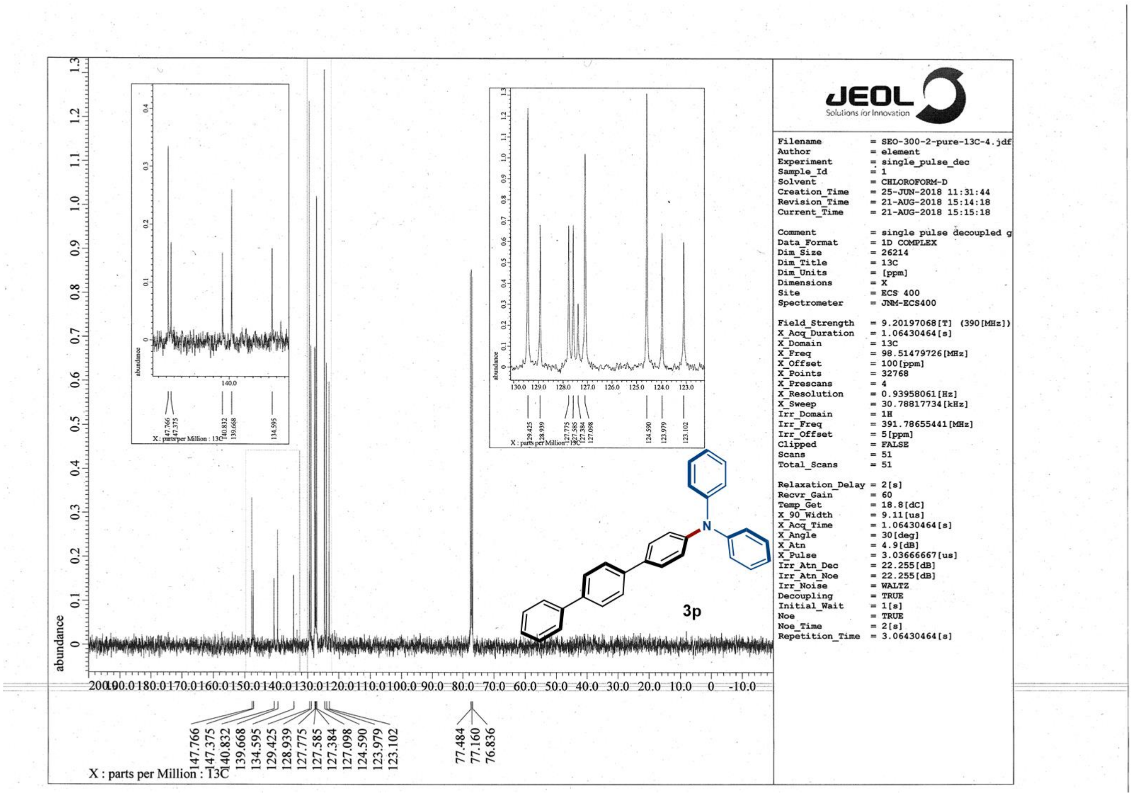

Supplementary Figure 41. ^{13}C NMR spectrum of **3p**.

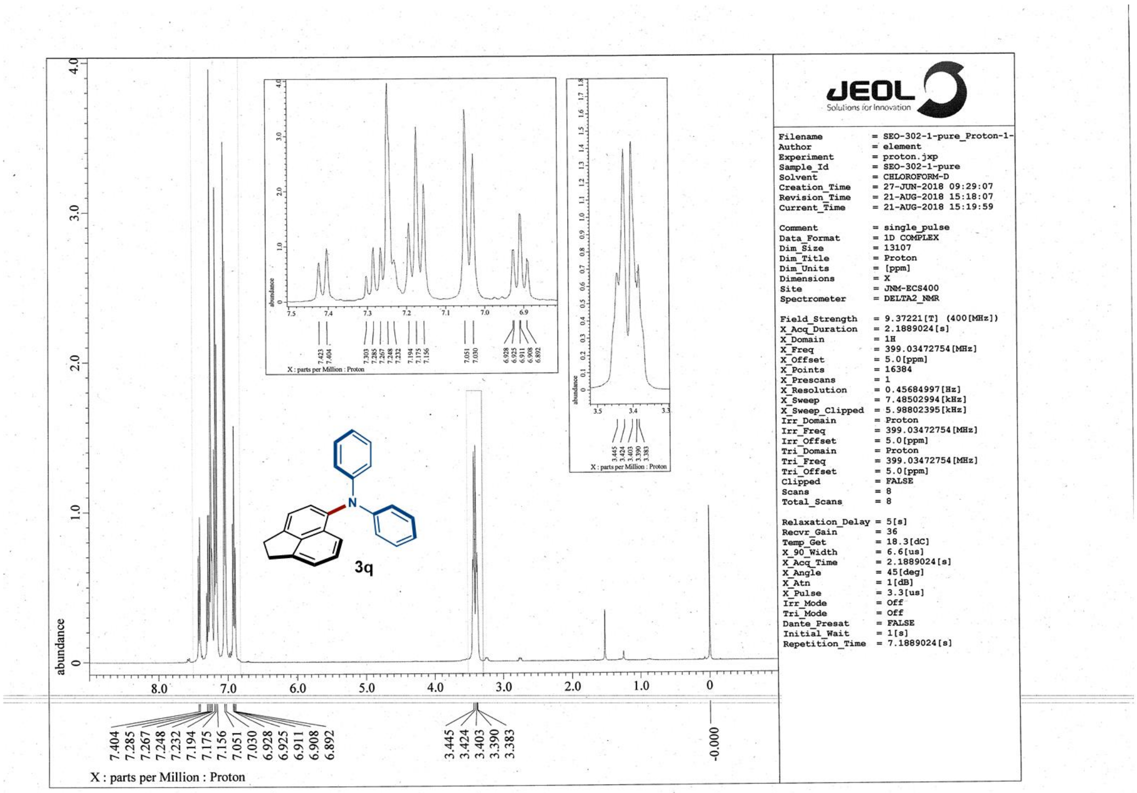

Supplementary Figure 42. ^{1}H NMR spectrum of **3q**.

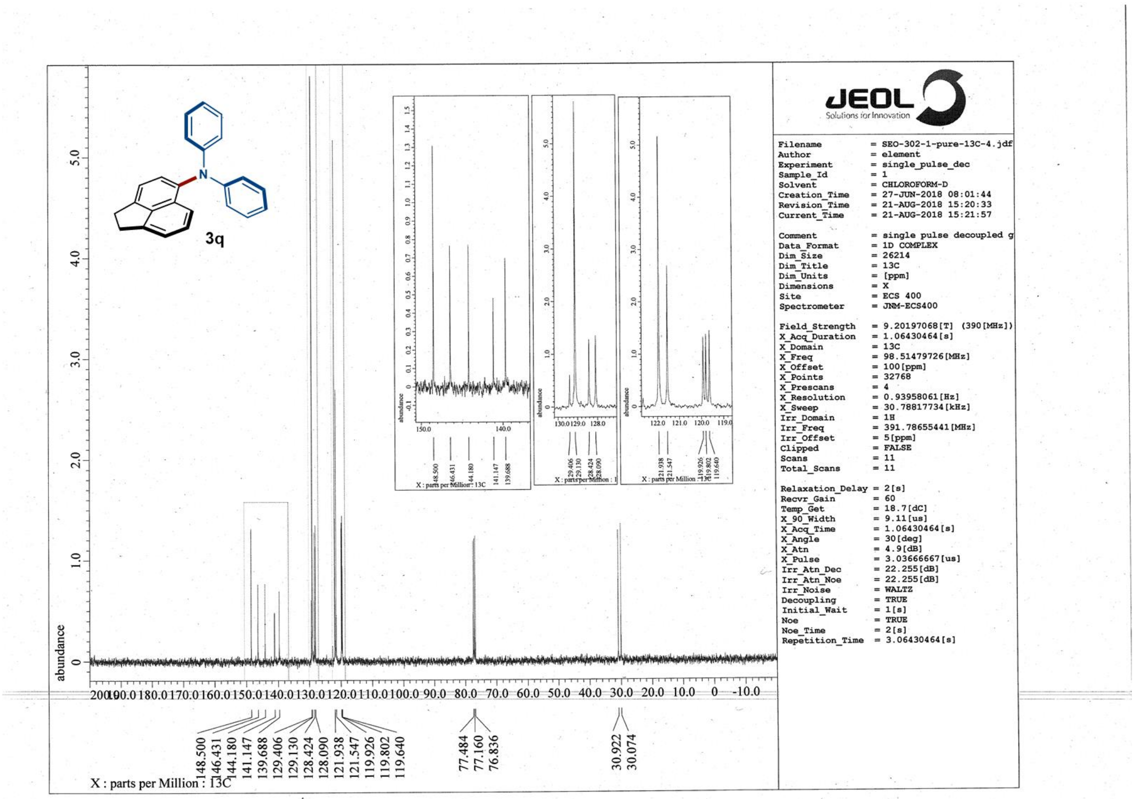

Supplementary Figure 43. ^{13}C NMR spectrum of **3q**.

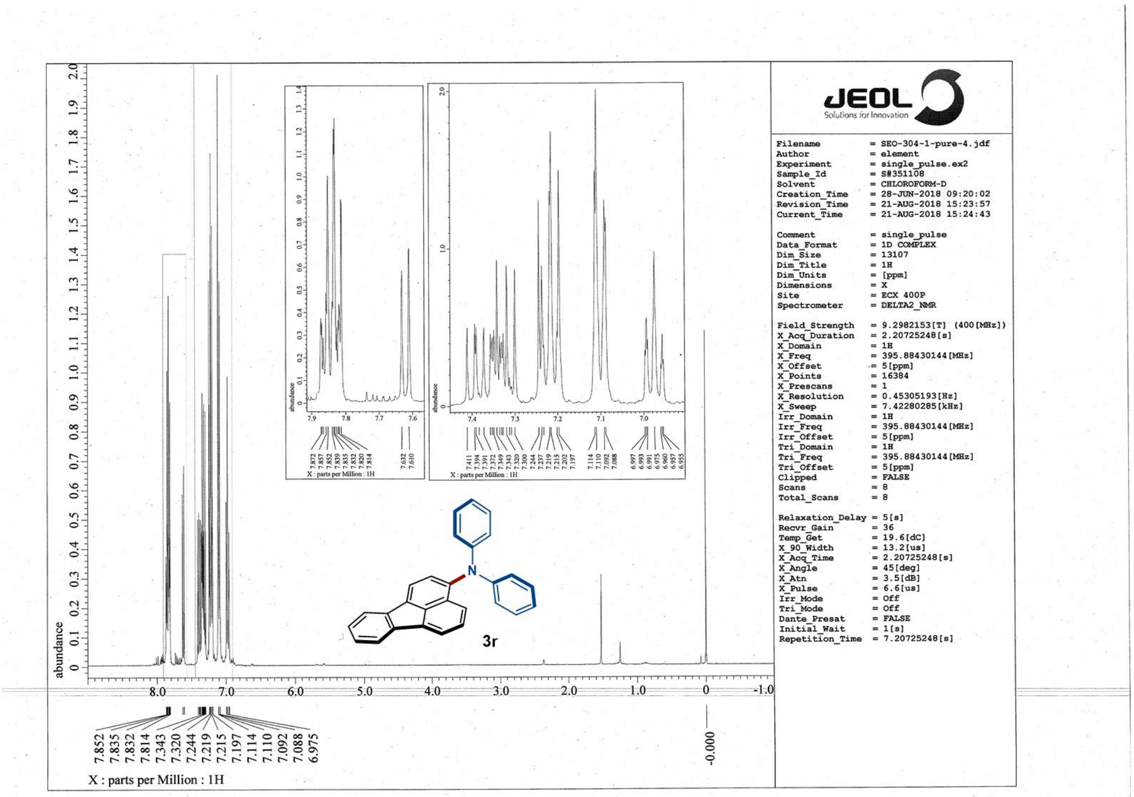

Supplementary Figure 44. ^{1}H NMR spectrum of **3r**.

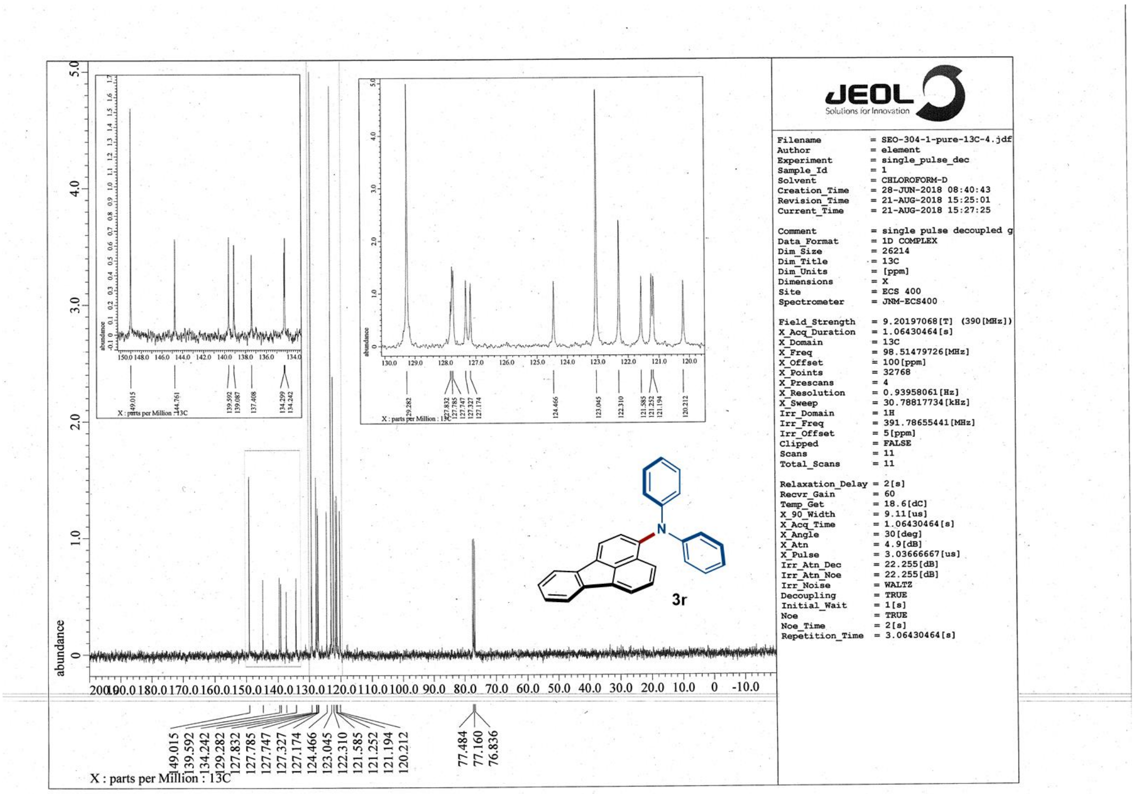

Supplementary Figure 45. ^{13}C NMR spectrum of **3r**.

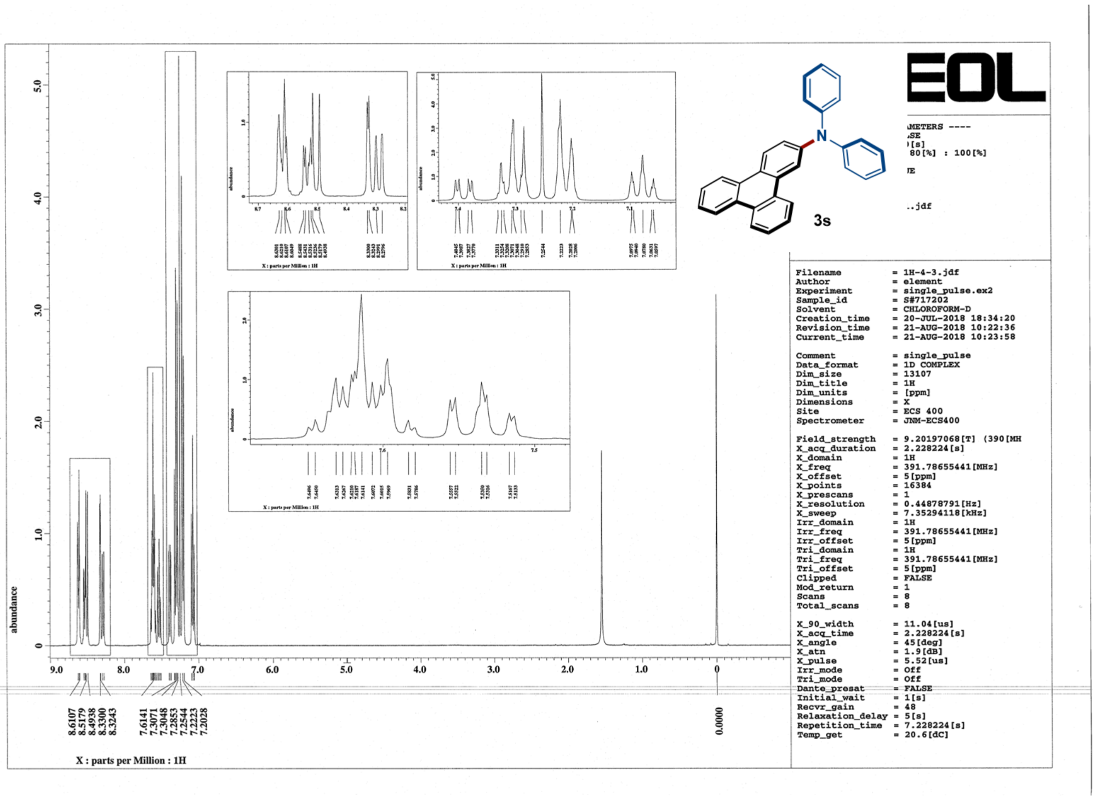

Supplementary Figure 46. ^{1}H NMR spectrum of **3s**.

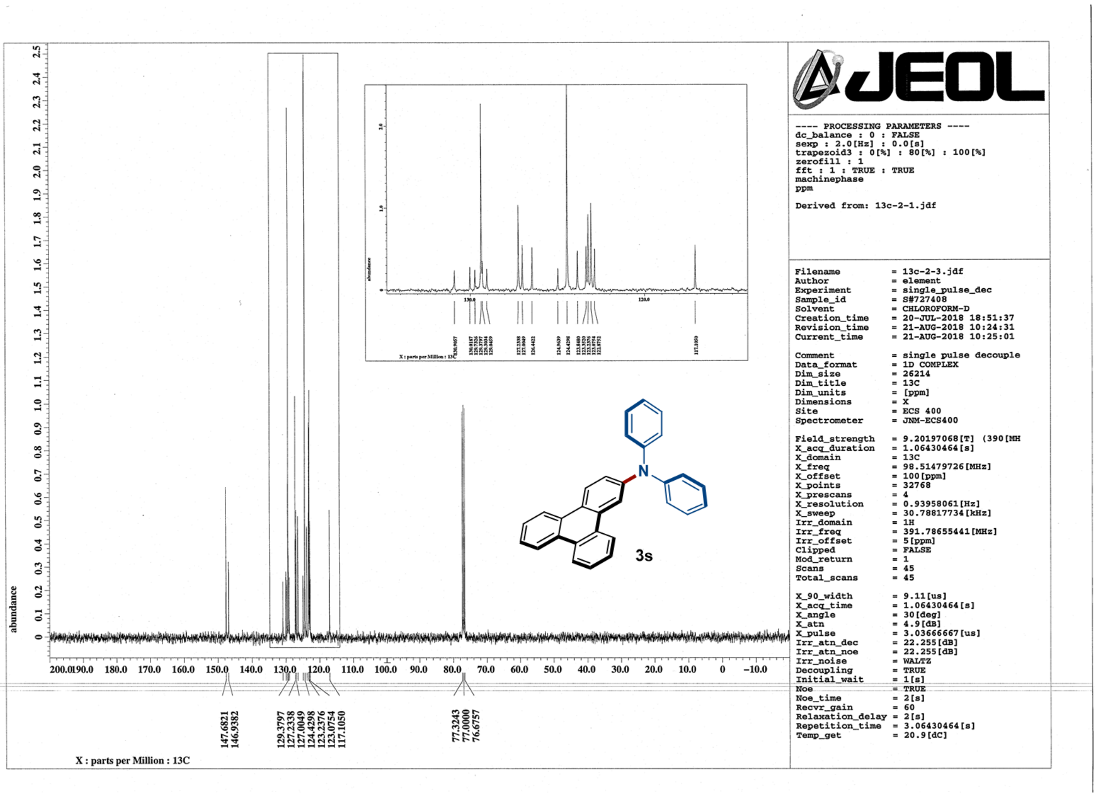

Supplementary Figure 47. ^{13}C NMR spectrum of **3s**.

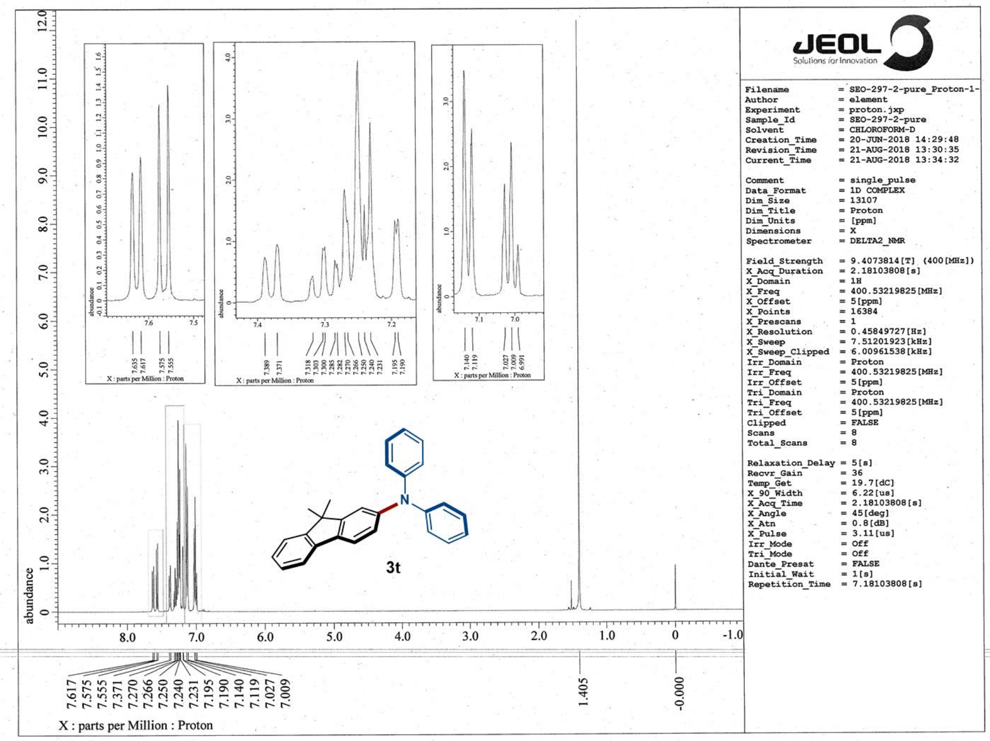

Supplementary Figure 48. ^{1}H NMR spectrum of **3t**.

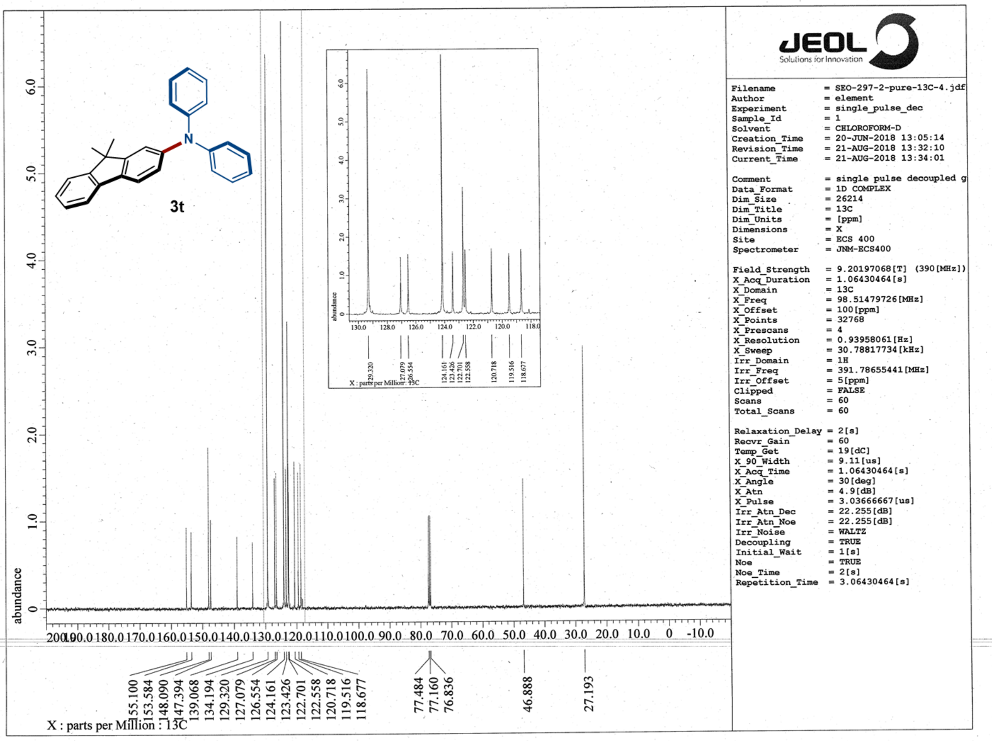

Supplementary Figure 49. ^{13}C NMR spectrum of **3t**.

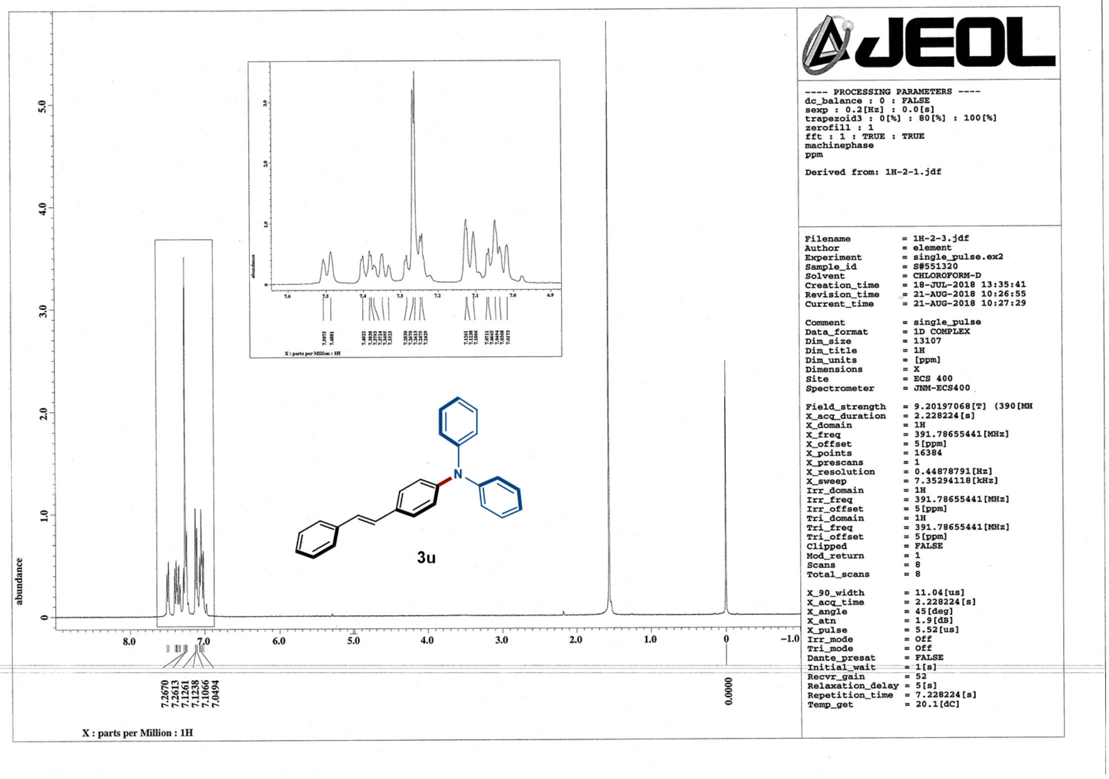

Supplementary Figure 50. ^{1}H NMR spectrum of **3u**.

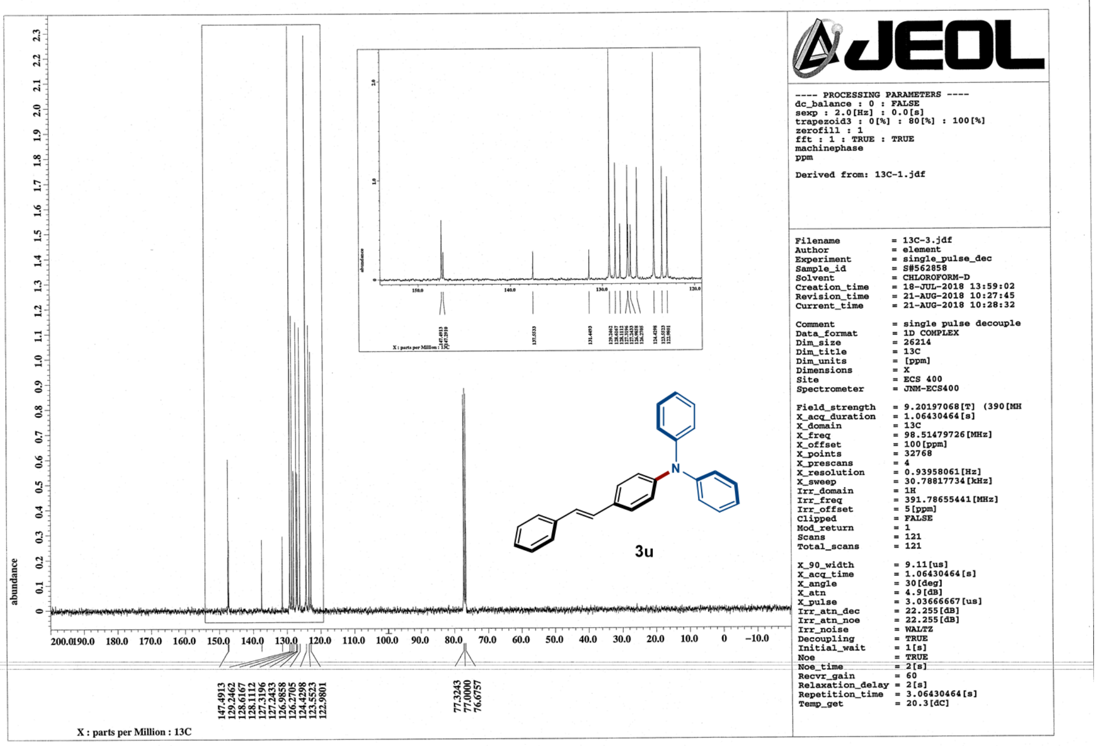

Supplementary Figure 51. ^{13}C NMR spectrum of **3u**.

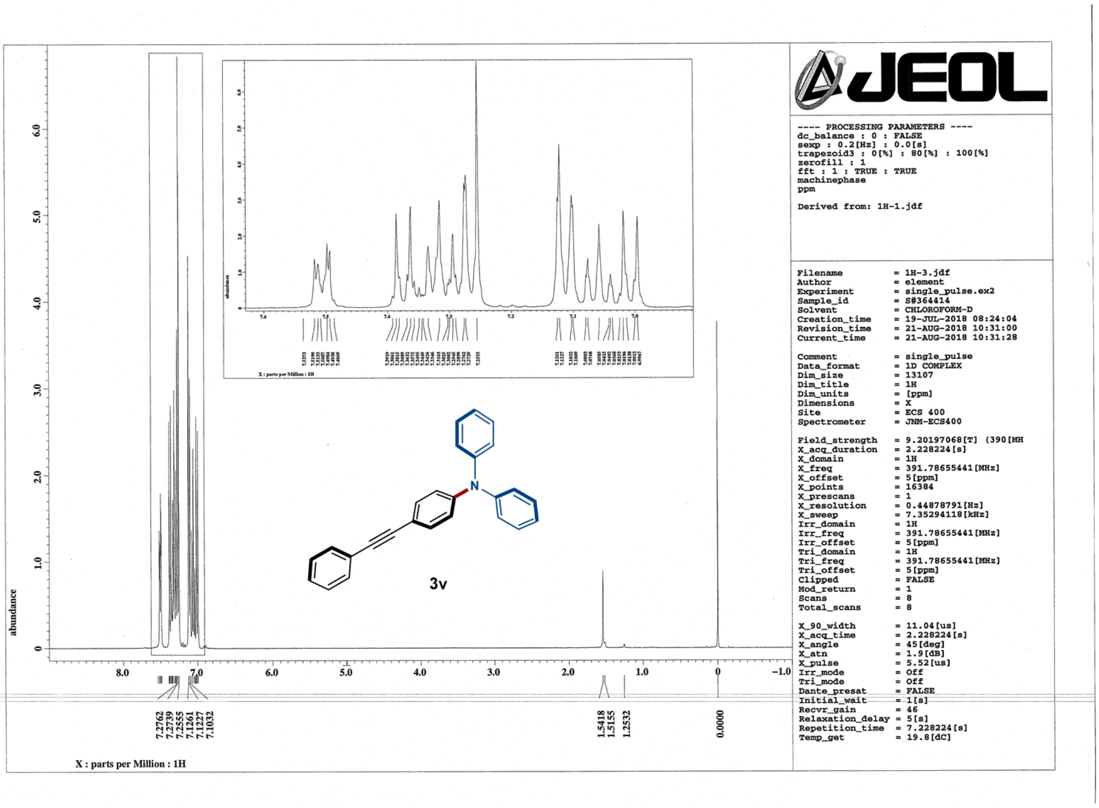

Supplementary Figure 52. ^{1}H NMR spectrum of **3v**.

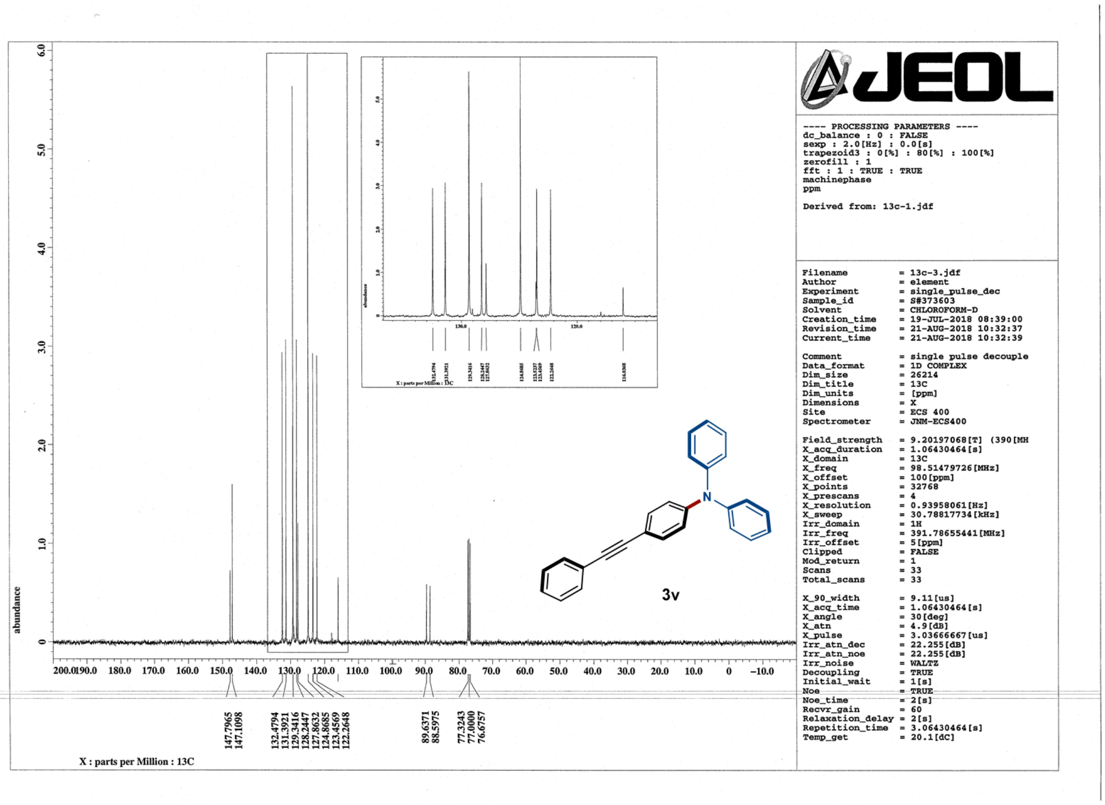

Supplementary Figure 53. ^{13}C NMR spectrum of **3v**.

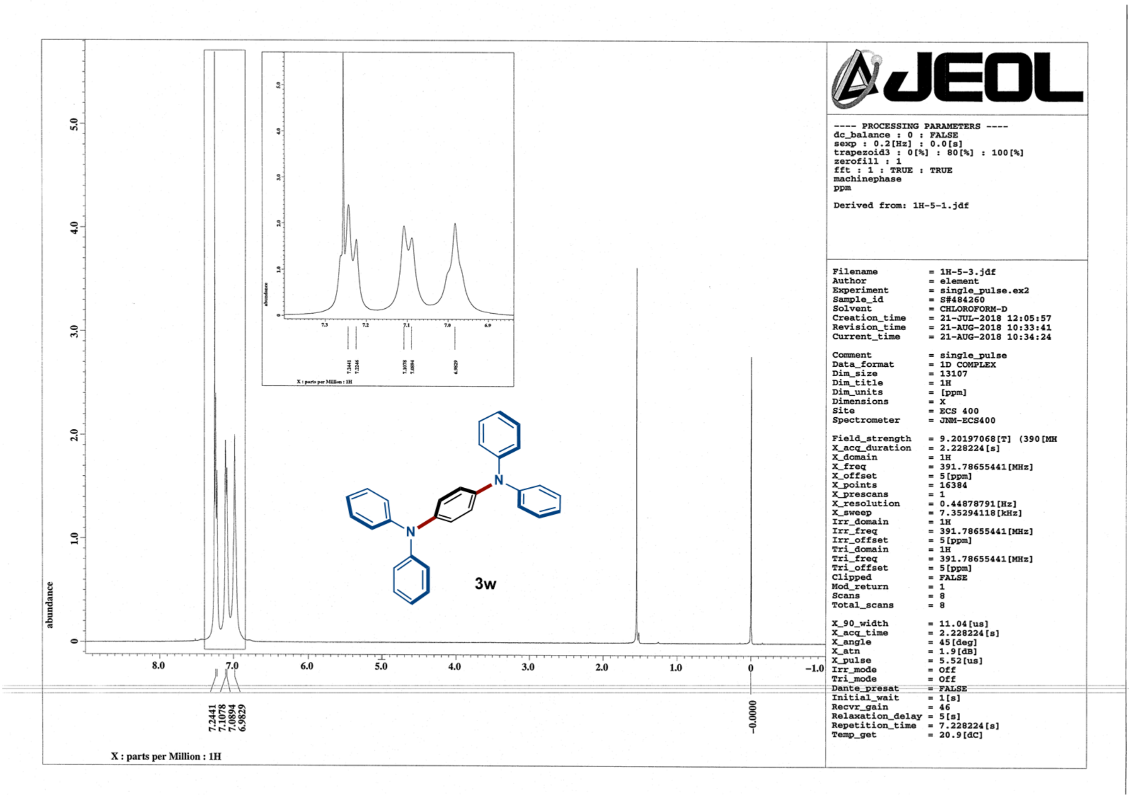

Supplementary Figure 54. ^{1}H NMR spectrum of **3w**.

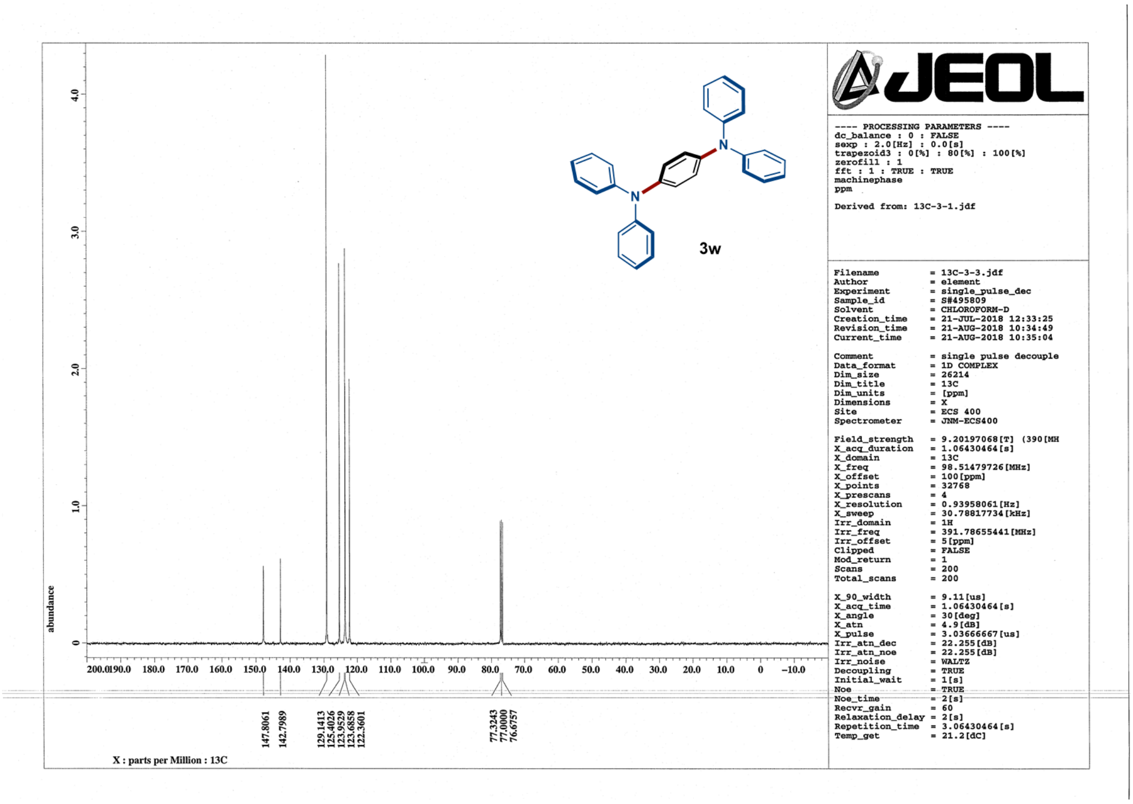

Supplementary Figure 55. ^{13}C NMR spectrum of **3w**.

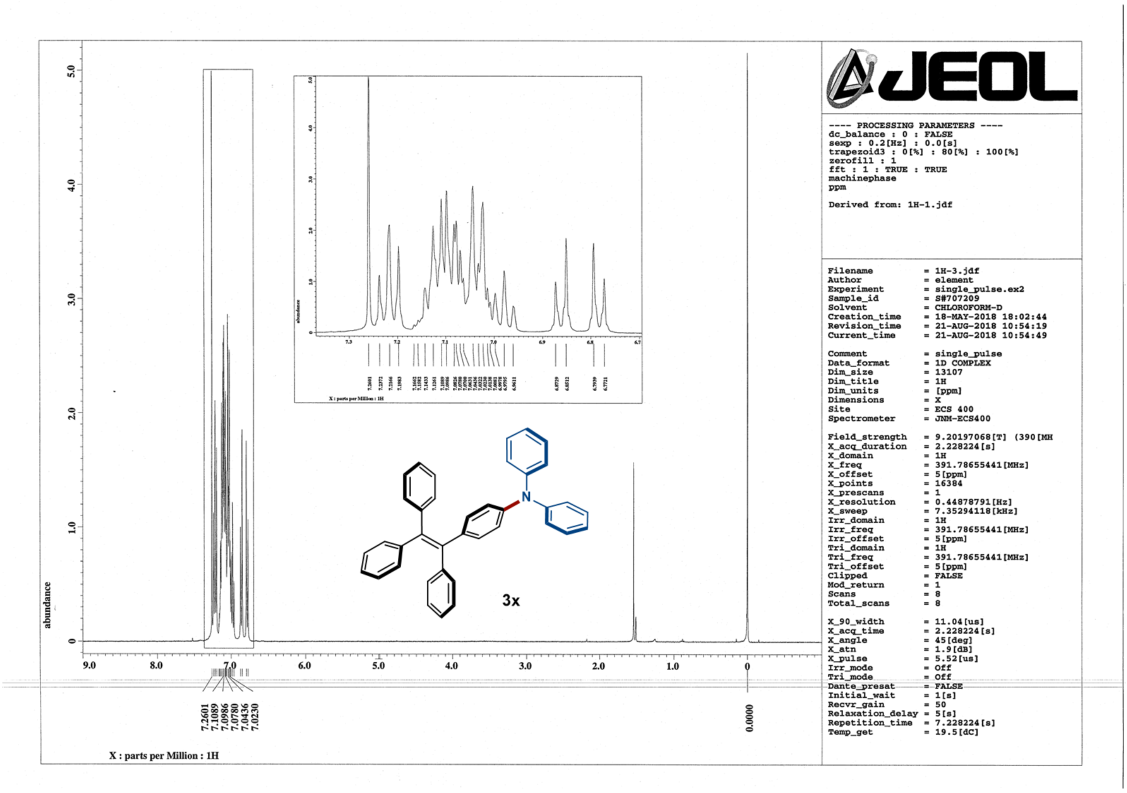

Supplementary Figure 56. ^{1}H NMR spectrum of **3x**.

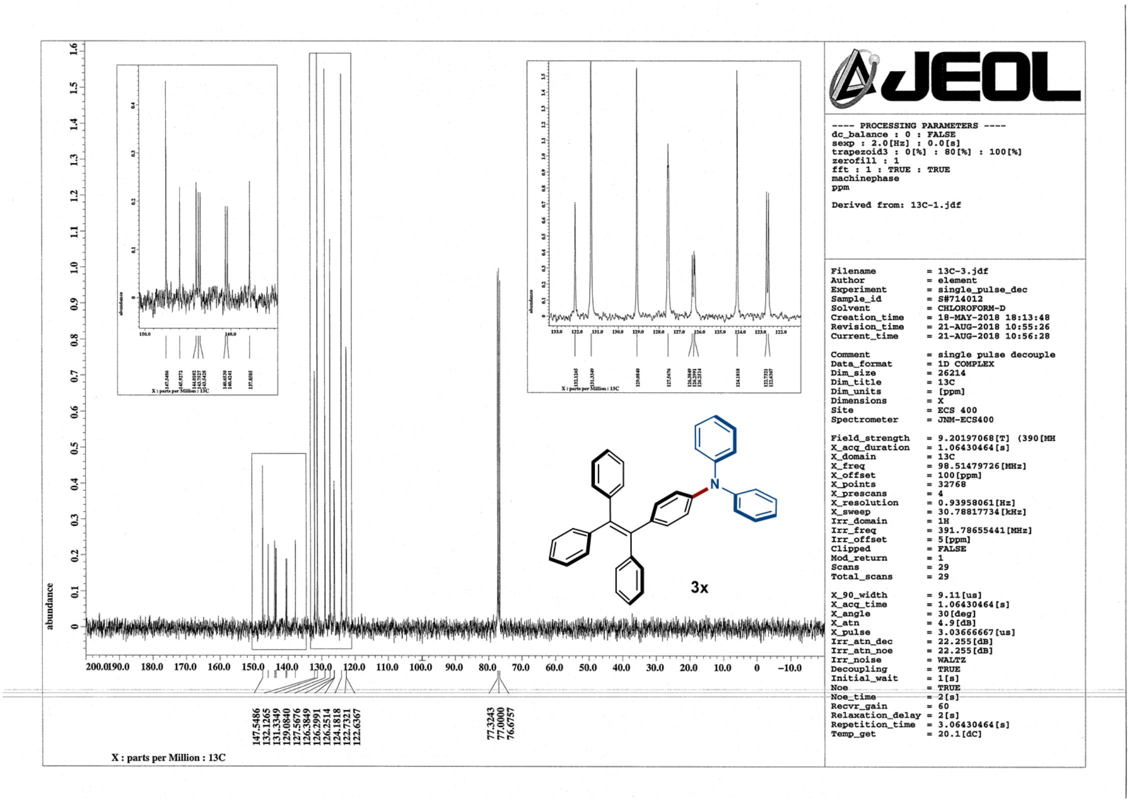

Supplementary Figure 57. ^{13}C NMR spectrum of **3x**.

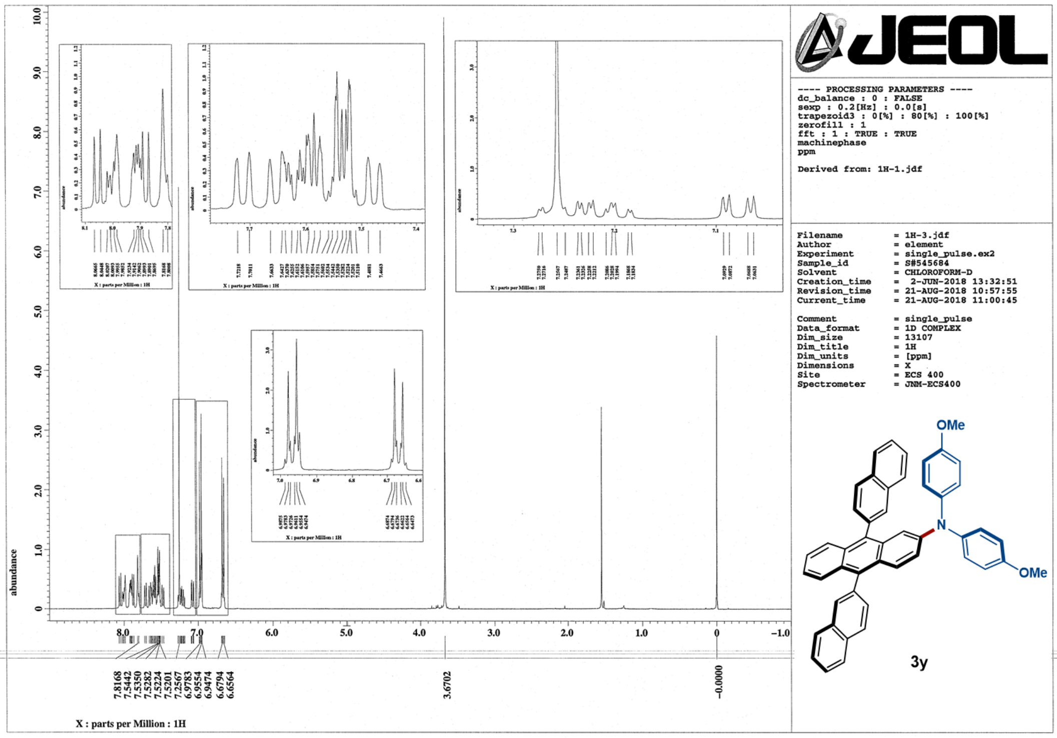

Supplementary Figure 58. ^{1}H NMR spectrum of **3y**.

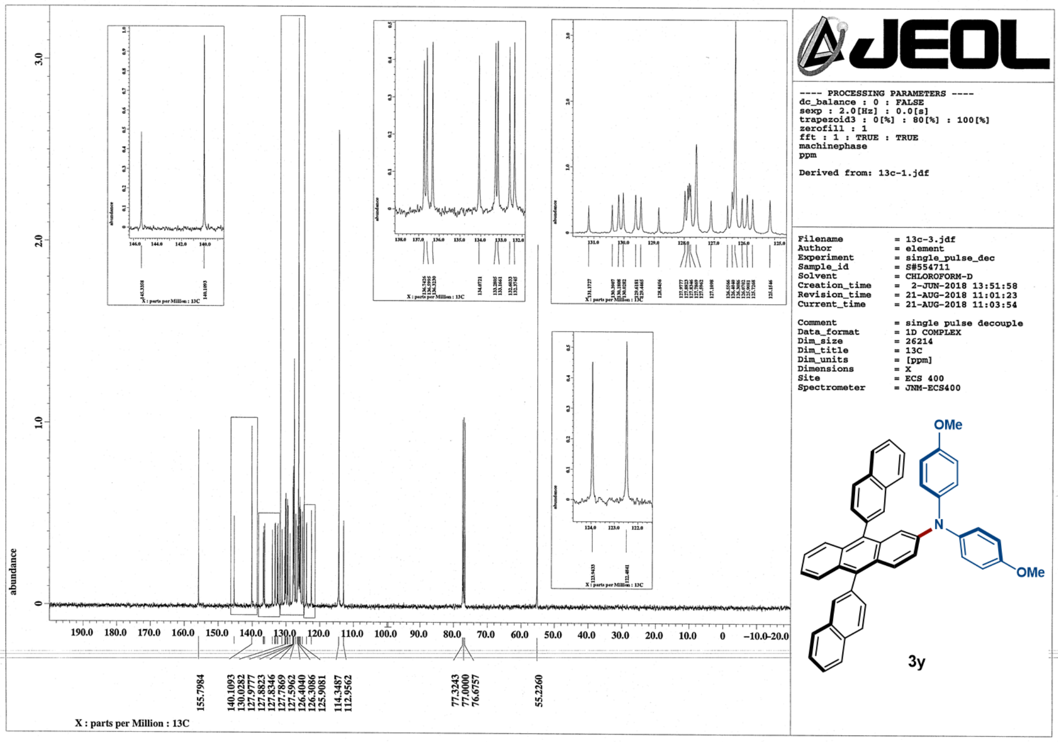

Supplementary Figure 59. ^{13}C NMR spectrum of **3y**.

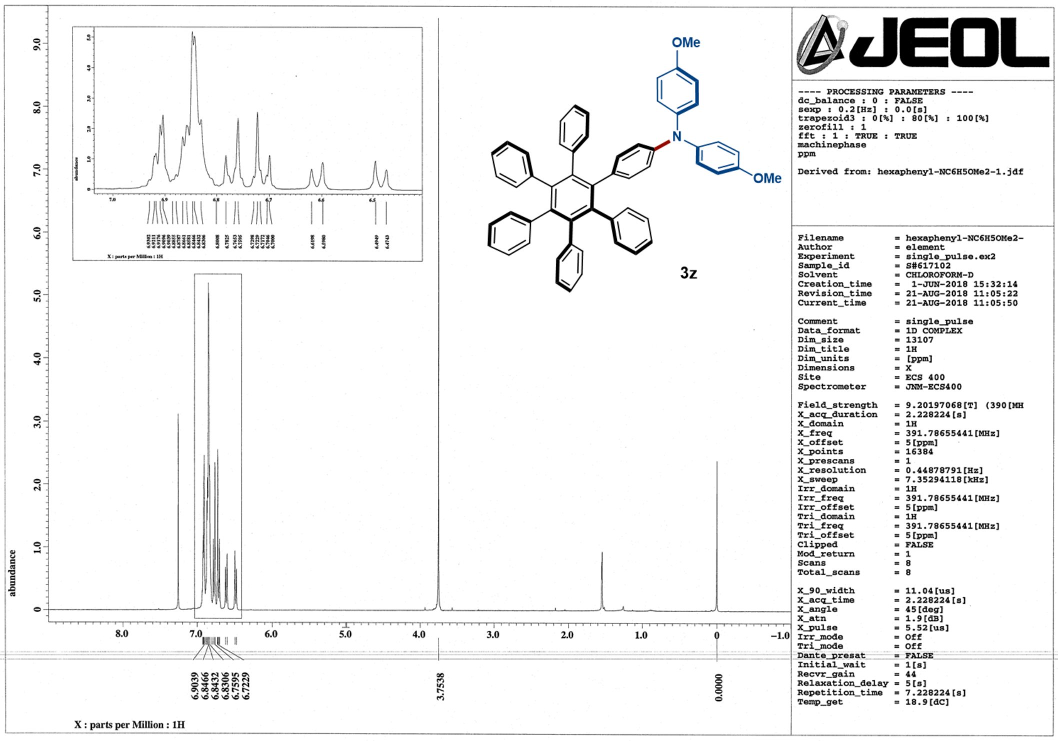

Supplementary Figure 60. ^{1}H NMR spectrum of **3z**.

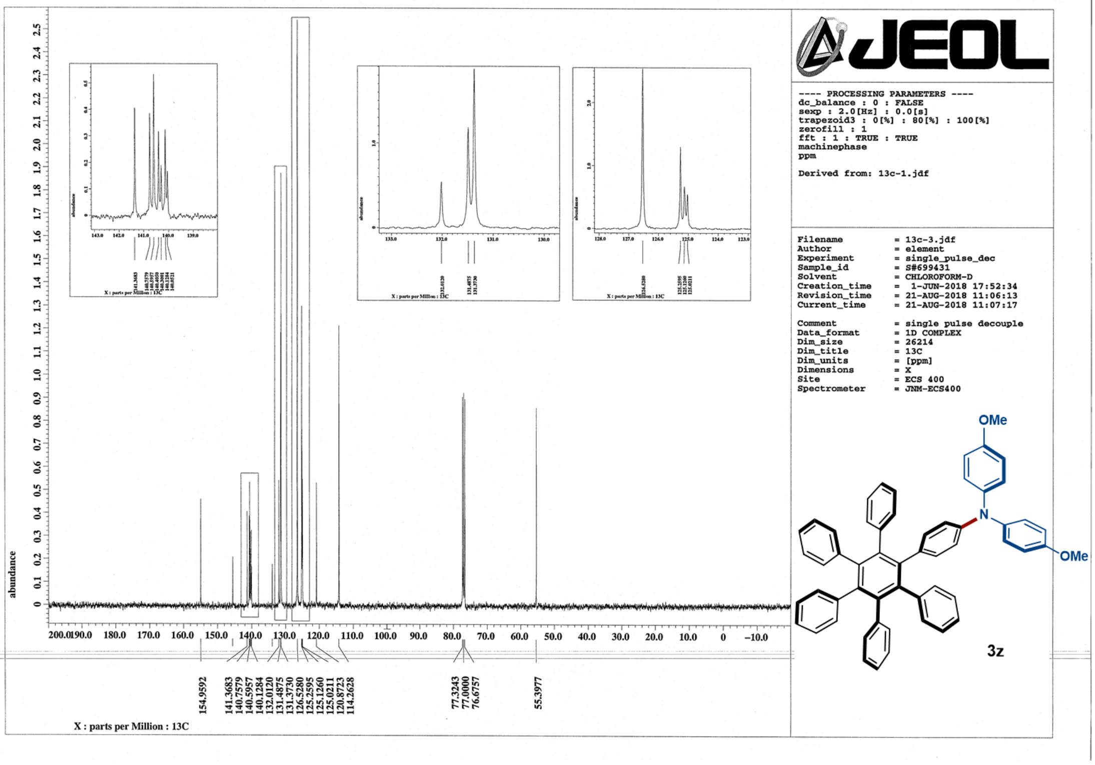

Supplementary Figure 61. ^{13}C NMR spectrum of **3z**.

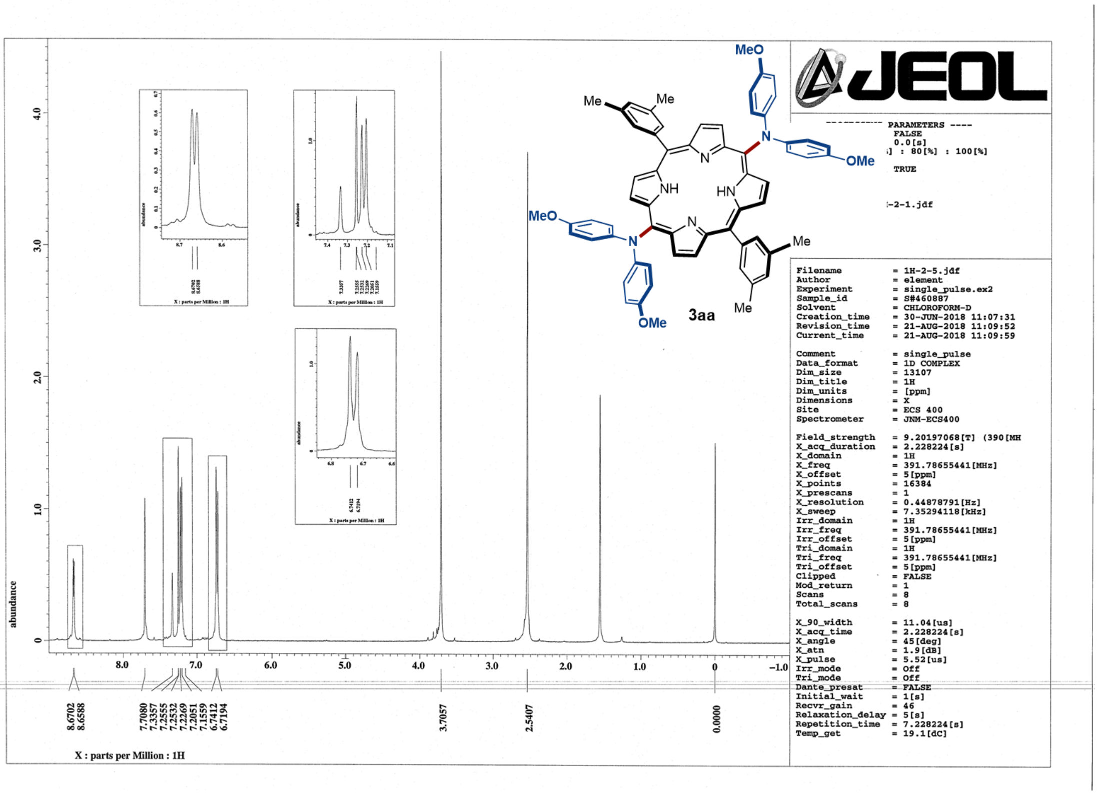

Supplementary Figure 62. ^{1}H NMR spectrum of **3aa**.

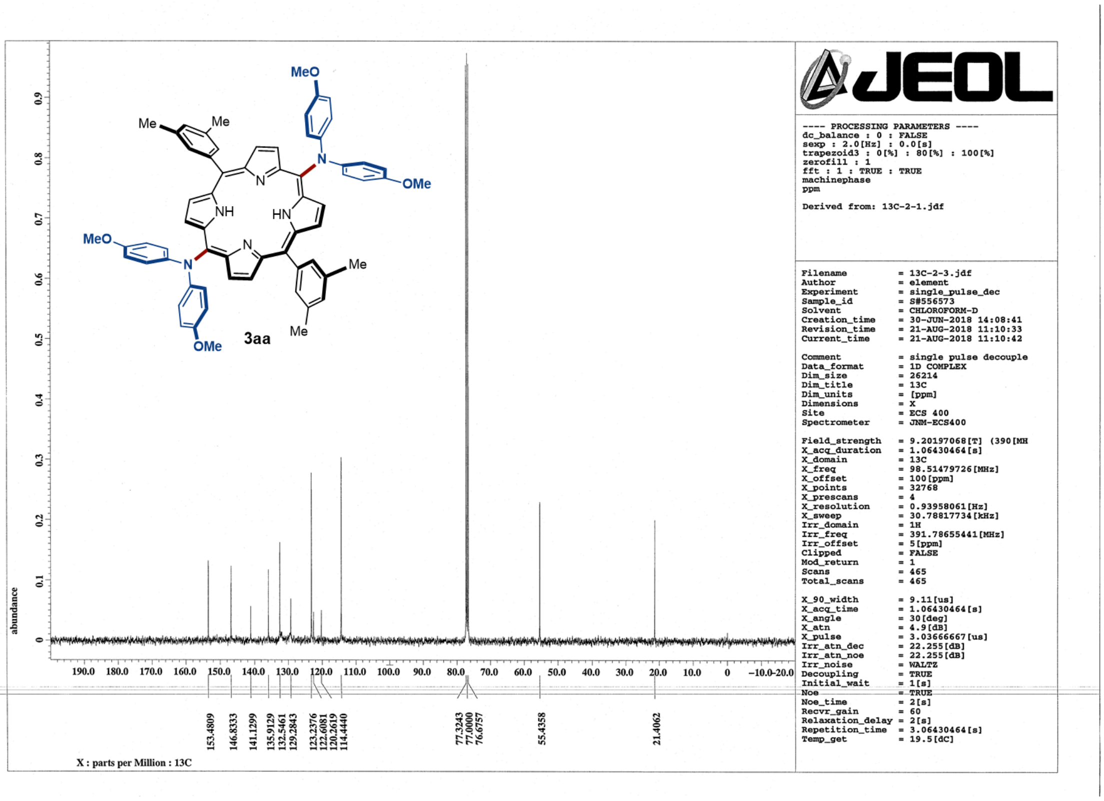

Supplementary Figure 63. ^{13}C NMR spectrum of **3aa**.

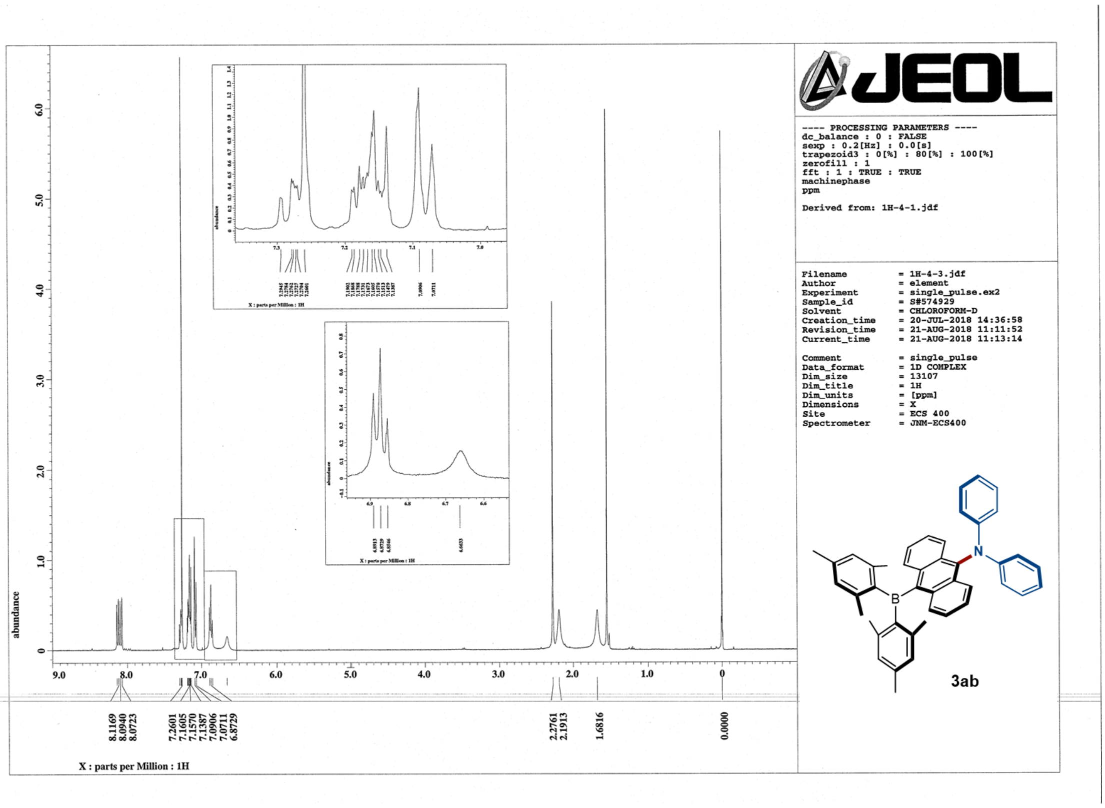

Supplementary Figure 64. ^{1}H NMR spectrum of **3ab**.

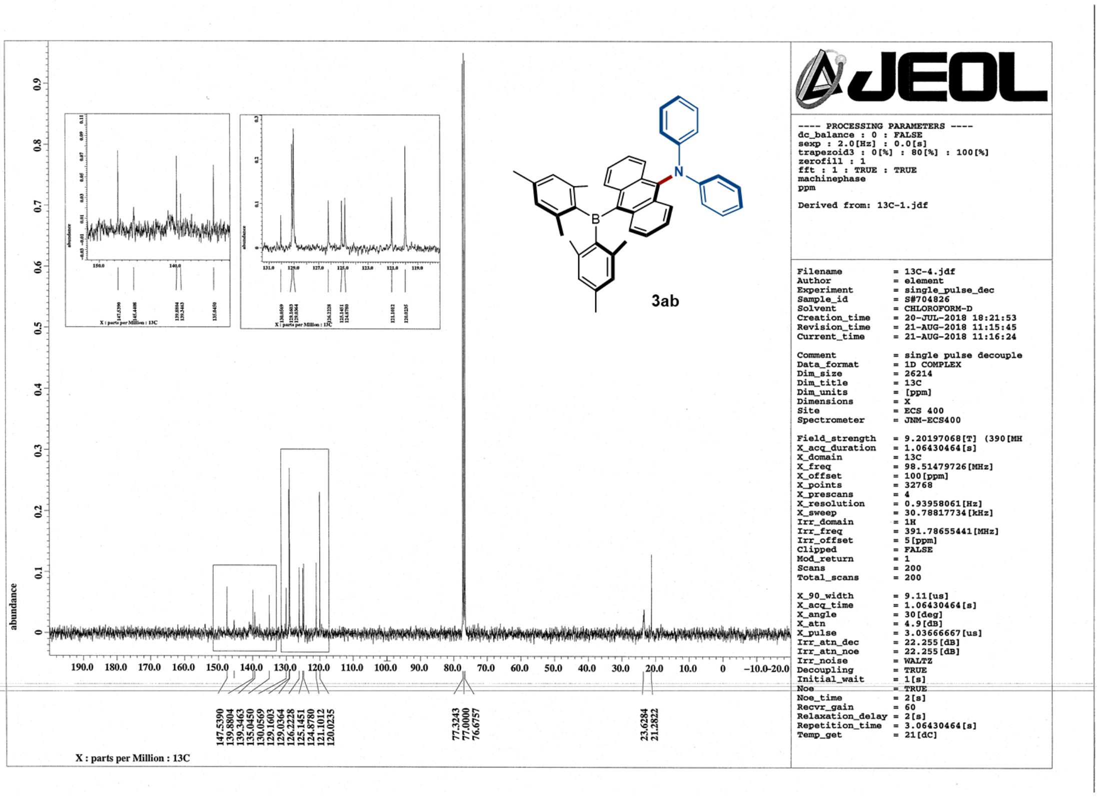

Supplementary Figure 65. ^{13}C NMR spectrum of **3ab**.

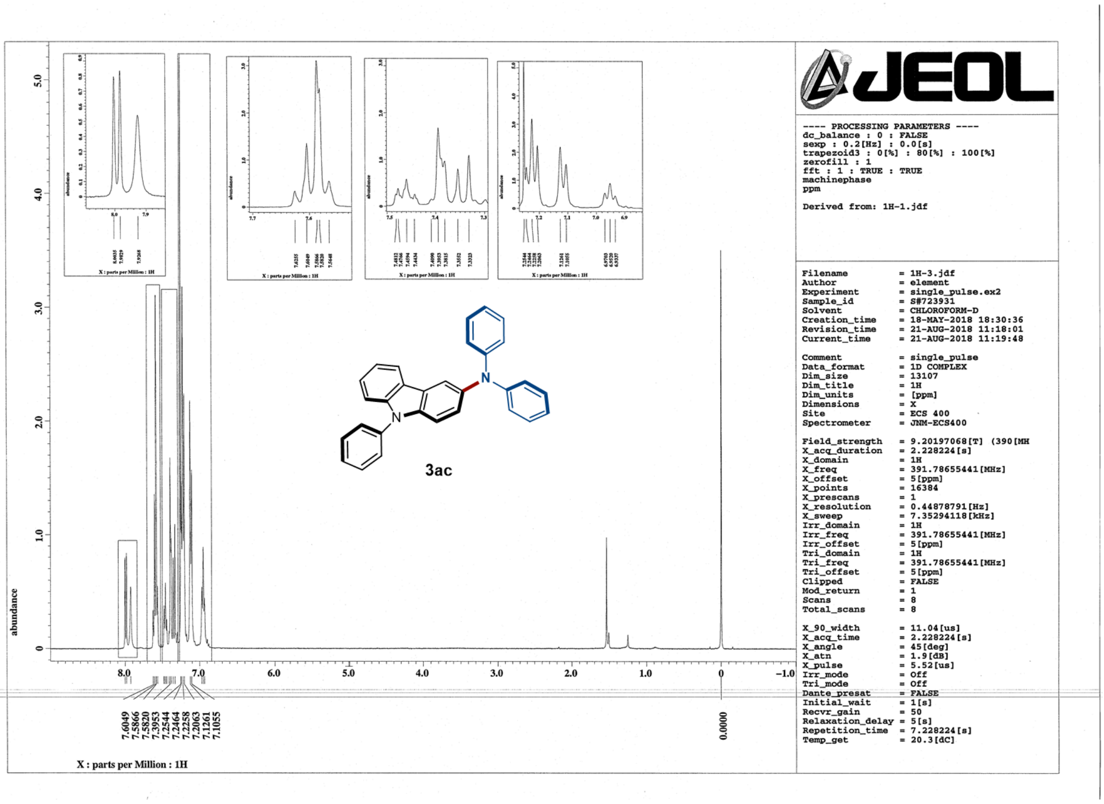

Supplementary Figure 66. ^{1}H NMR spectrum of **3ac**.

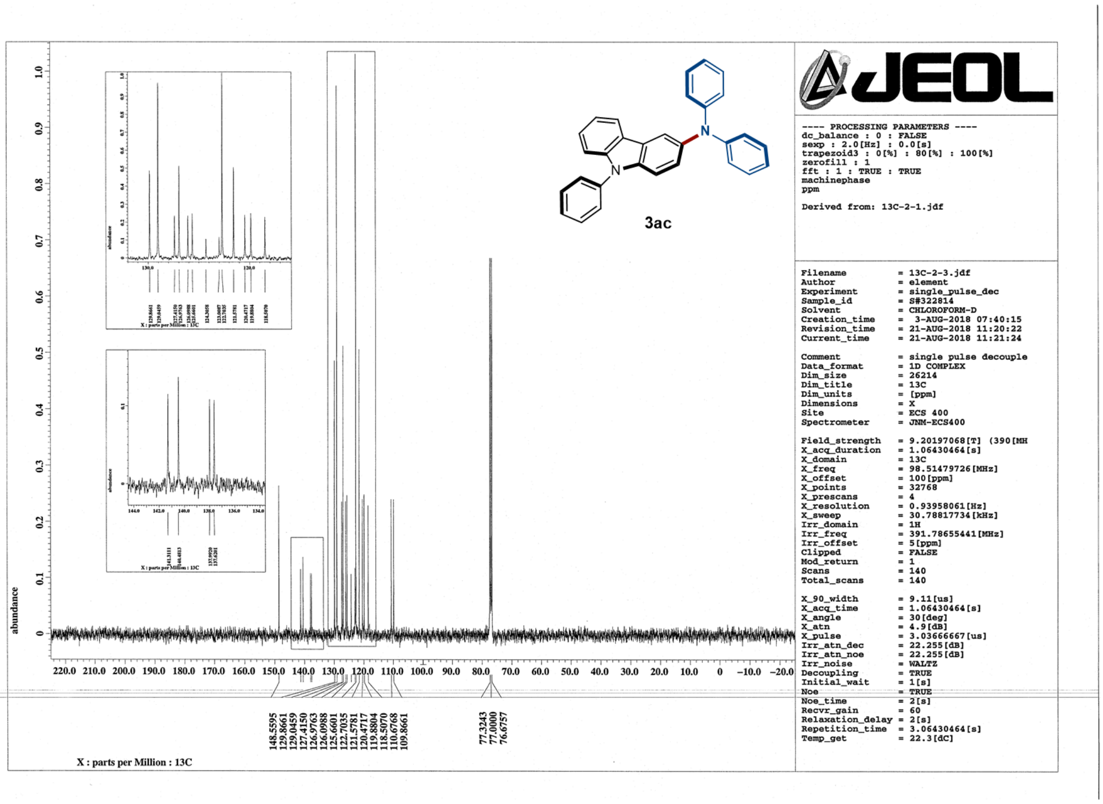

Supplementary Figure 67. ^{13}C NMR spectrum of **3ac**.

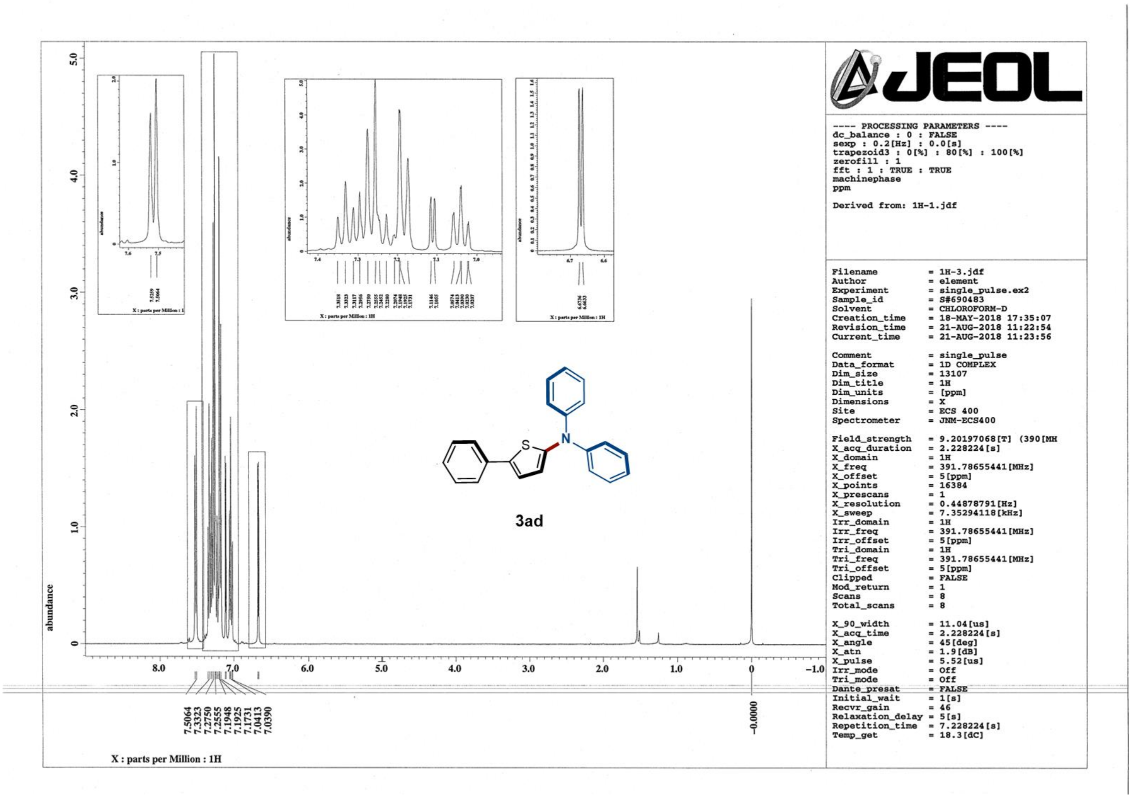

Supplementary Figure 68. ^{1}H NMR spectrum of **3ad**.

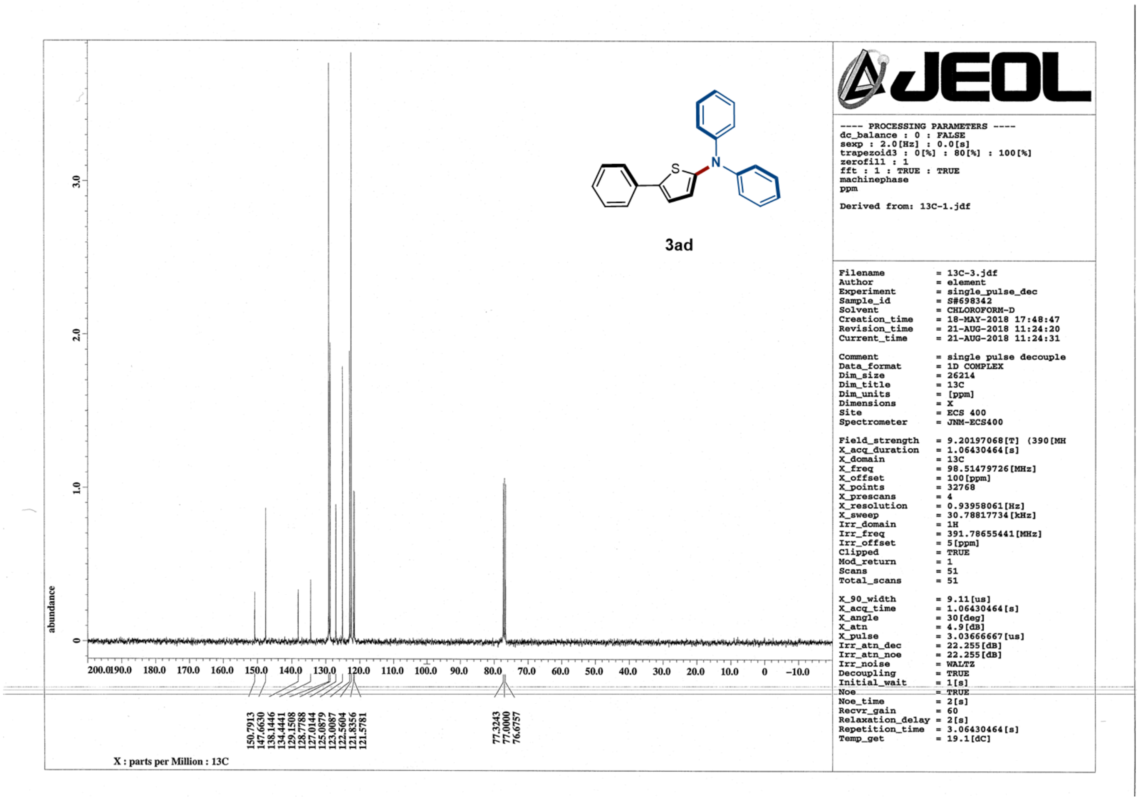

Supplementary Figure 69. ^{13}C NMR spectrum of **3ad**.

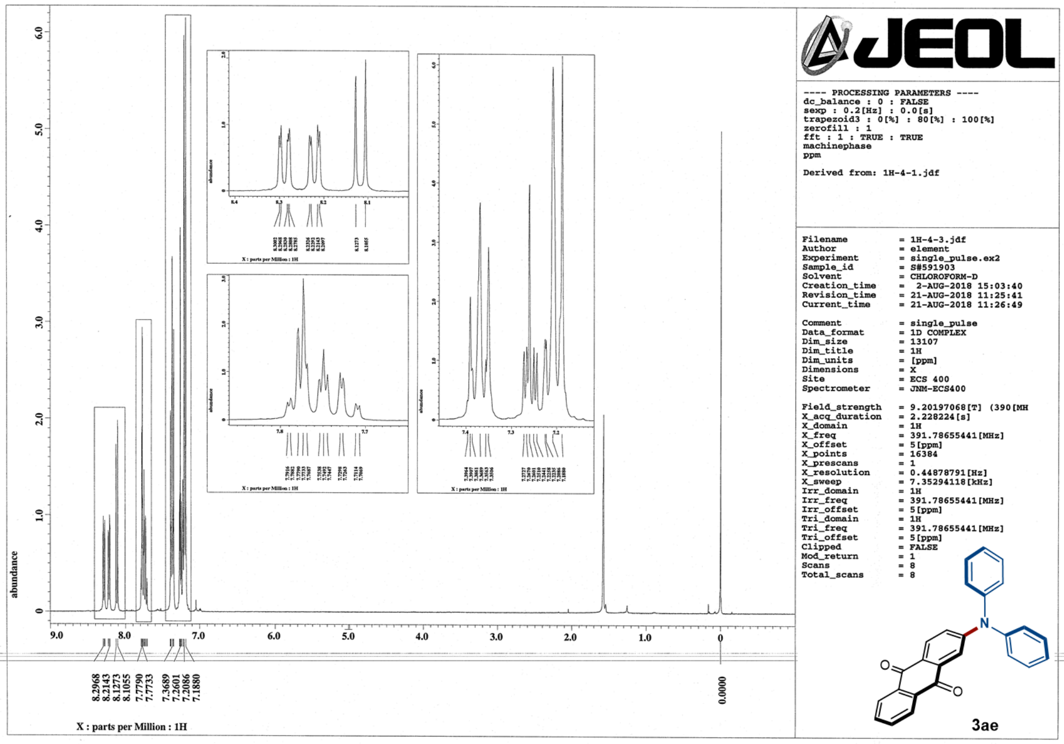

Supplementary Figure 70. ^{1}H NMR spectrum of **3ae**.

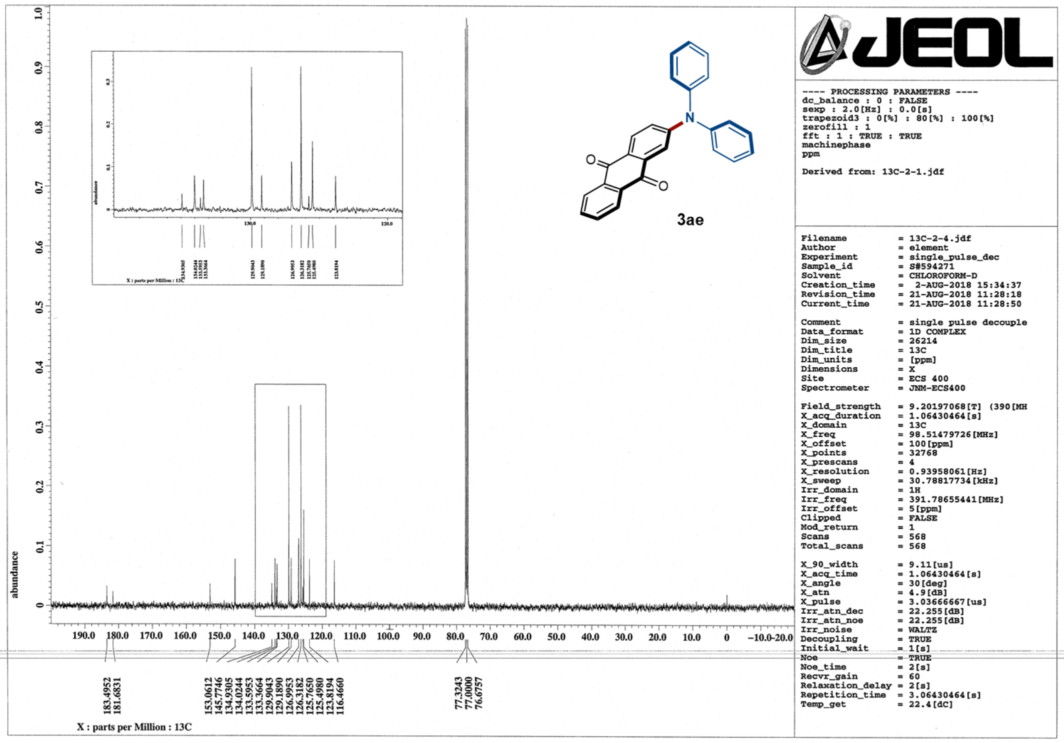

Supplementary Figure 71. ^{13}C NMR spectrum of **3ae**.

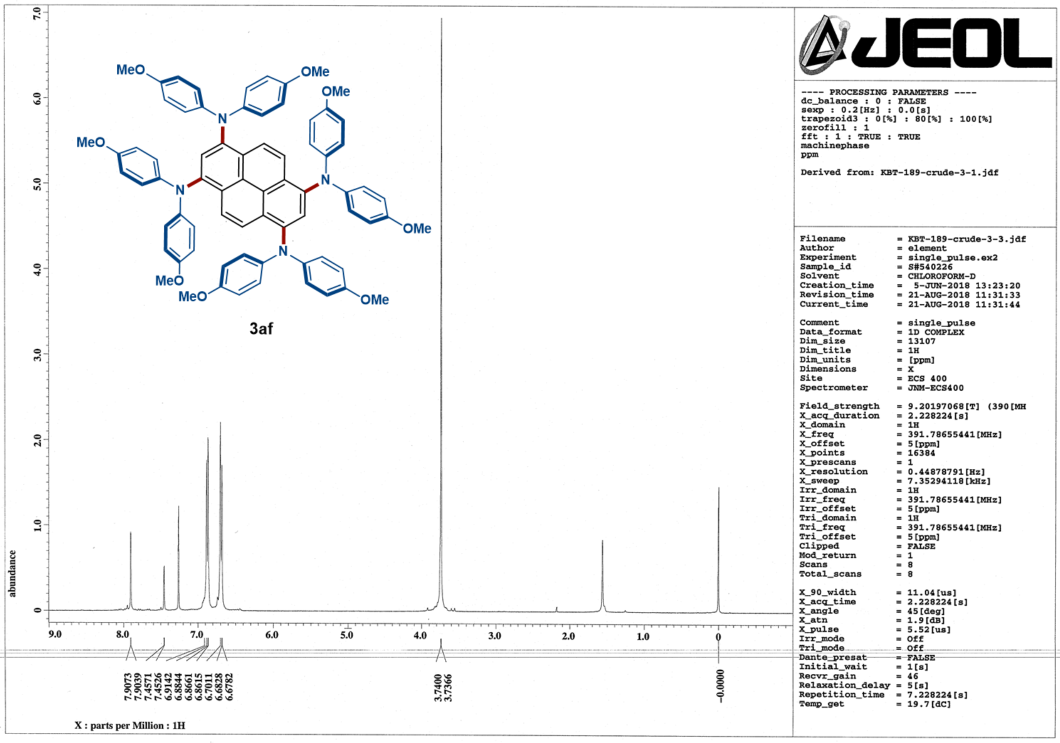

Supplementary Figure 72. ^{1}H NMR spectrum of **3af**.

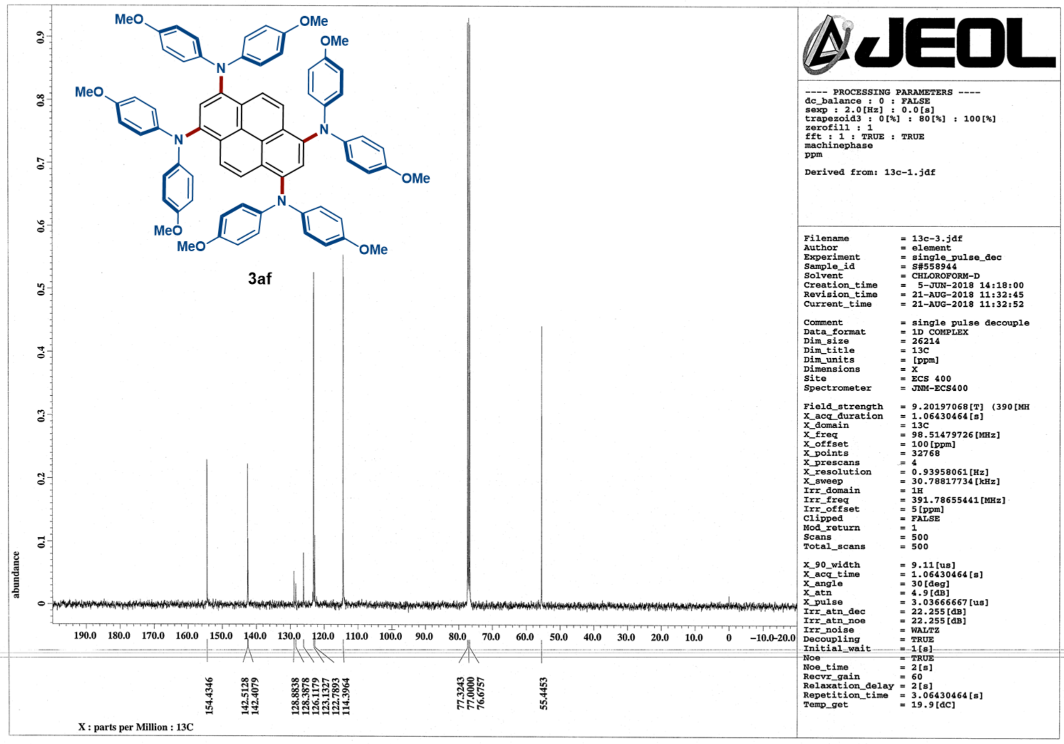

Supplementary Figure 73. ^{13}C NMR spectrum of **3af**.

Supplementary References

1. Kübel, C., Chen, S. –L., Müllen, K. *Macromolecules*, **31**, 6014–6021. (1998)
2. Abada, Z., Ferrié, L., Akagah, B., Lormier, A. T., Figadère, B. *Tetrahedron Lett.* **52**, 3175–3178. (2011).
3. Abada, Z., Ferrié, L., Akagah, B., Lormier, A. T., Figadère, B. *Tetrahedron Lett.* **53**, 6961–6964. (2012).
4. Jia, W. –L., Bai, D. –R., McCormick, T., Liu, Q. –D., Motala, M., Wang, R. –Y., Seward, C., Tao, Y., Wang, S. *Chem. Eur. J.* **10**, 994–1006. (2004).
5. Topchiy, M. A., Asachenko, A. F., Nechaev, M. S. *Eur. J. Org. Chem*. **16**, 994–1006. (2004).
6. Hu, J. –Y., Feng, X., Seto, N., Do, J. –H., Zeng, X., Tao, Z., Yamato, T. *J. Mol. Struct.* **1035**, 19–26. (2013).
7. Nie, H. –J., Yao, C. –J., Shao, J. –Y., Yao, J., Zhong, Y. –W. *Chem. Eur. J.* **20**, 17454–17465. (2014).
8. Tadaoka, H., Yamakawa, T. *Tetrahedron Lett.* **53**, 5531–5534. (2012).
9. Xie, C., Zhang, Y. *Org. Lett.* **9**, 781–784. (2007).
10. Monguchi, Y., Kitamoto, K., Ikawa, T., Maegawa, T., Sajiki, H. *Adv. Synth. Catal.* **350**, 2767–2777. (2008).
11. Yang, J. –S., Chiou, S. –Y., Liau, K. –L. *J. Am. Chem. Soc.* **124**, 2518–2527. (2002).
12. Deol, H., Pramanik, S., Kumar, M., Khan, I., Bhalla, V. *ACS Catal*. **6**, 3771–3783. (2016).
13. Hartwig, J. F., Kawatsura, M., Hauck, S., Sheila, I., Shaughnessy, K. H., Alcazar-Roman, L. M. *J. Org. Chem.* **64**, 5575–5580. (1999).
14. Chan, C. Y. K., Lam, J. W. Y., Zhao, Z., Chen, S., Lu, P., Sung, H. H. Y., Kwok, H. S., Ma, Y., Williams, I. D., Tang, B. Z. *J. Mater. Chem.* **2**, 4320–4327. (2014).
15. Tabet, A., Hartmann, H. *Synthesis* **4**, 610–616. (2005).
16. Jeon, N. J., Lee, J., Noh, J. H., Nazeeruddin, M. K., Grätzel, M., Seok, S. *J. Am. Chem. Soc.* **135**, 19087–19090. (2013).
